# Supplementary material for: Menadione reduces the expression of virulence- and colonization-associated genes in Helicobacter pylori
Source: Microbiology (Reading). 2025 Mar 12;171(3):001539. doi: 10.1099/mic.0.001539 (PMC12282218; doi:10.1099/mic.0.001539)
Supplement: Uncited Supplementary Material 1. [file mic-171-01539-s001.pdf]

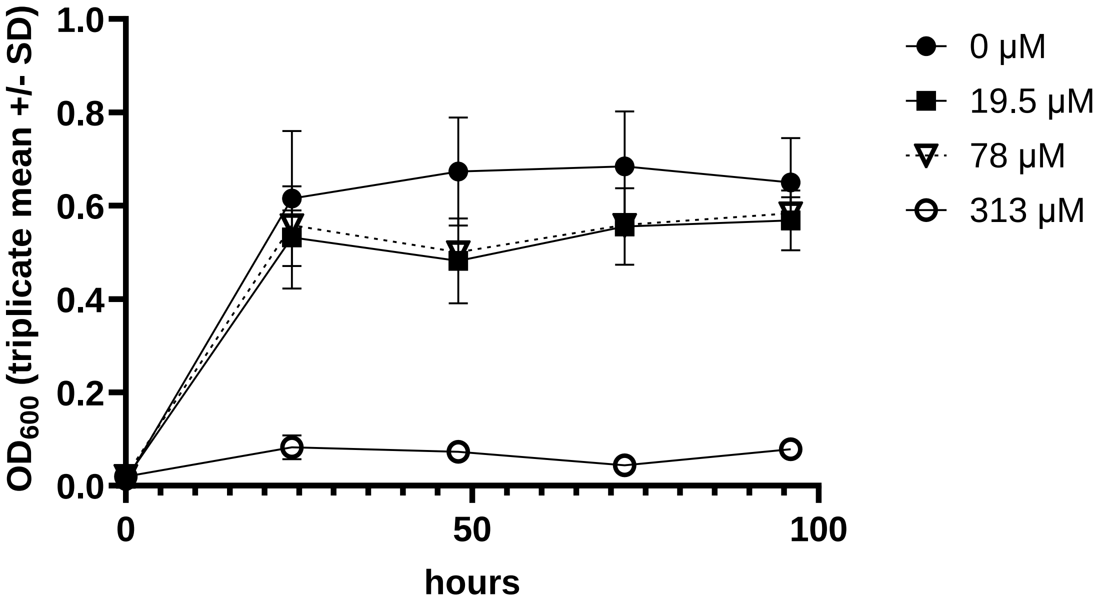


Supplementary Figure 1. Effects of menadione on the growth of *H. pylori* strain 322A. *H. pylori* strain 322A was incubated in Brucella broth + 5 % heat-inactivated foetal calf serum, with the indicated concentrations of menadione, final volume 100 µl/well in 96-well plates at 37 °C under microaerobic conditions. Bacterial growth was monitored by measuring the $OD_{600}$ at 0, 24, 48, 72 and 96 h. Data shown are blank corrected means +/- SD for triplicate wells. Menadione at 313 µM significantly inhibited *H. pylori* growth, but the modest reductions in $OD_{600}$ with 78 µM and 19.5 µM menadione did not reach statistical significance ($p>0.05$, 2-way ANOVA with Dunnett's post-hoc test).

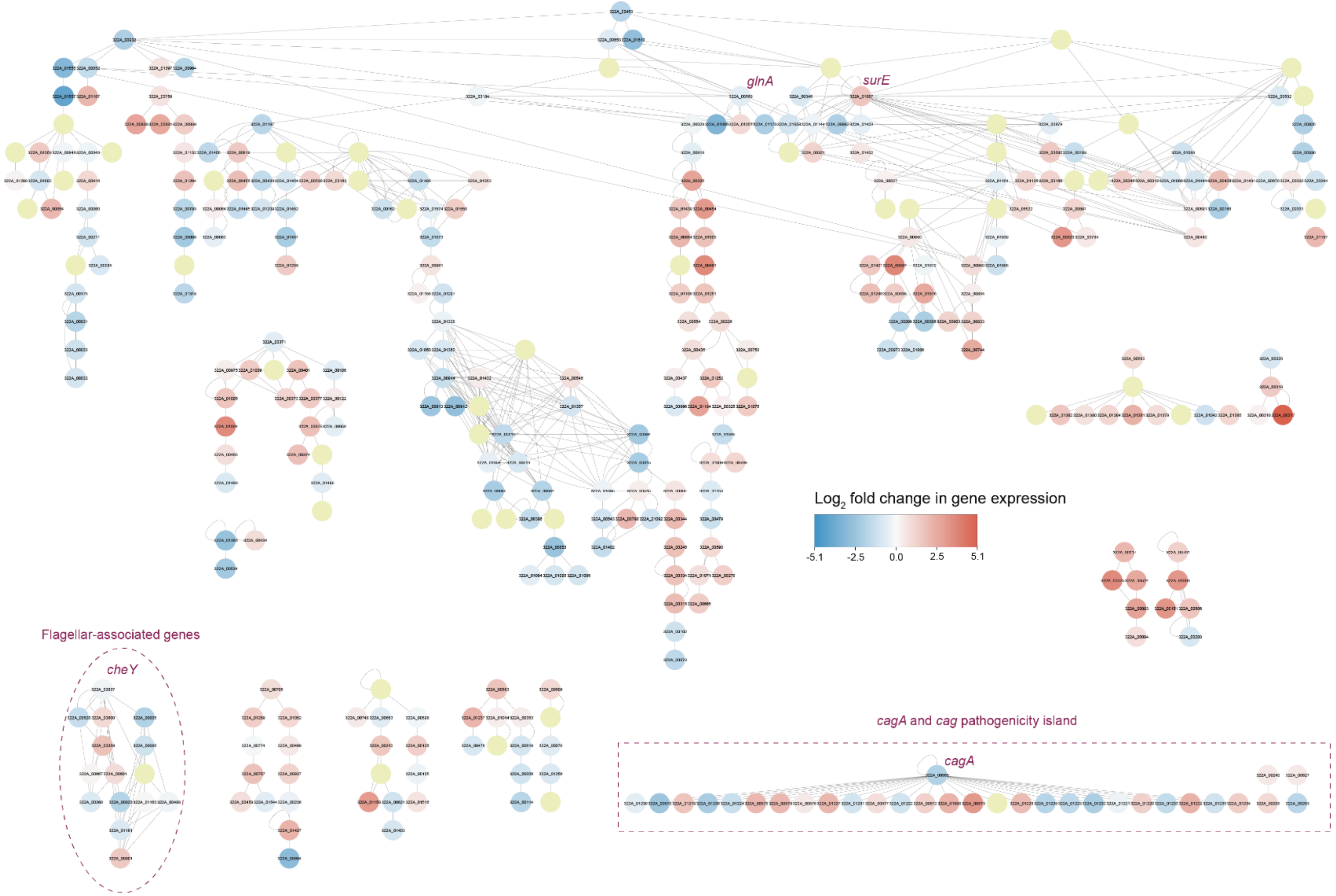


Supplementary Figure 2. Network-based analysis of strain 322A genes that could be mapped to KEGG metabolic pathways. Significantly differentially expressed genes (adjusted $P < 0.05$; Benjamini-Hochberg procedure) are shown by blue (downregulated by menadione) and red (upregulated by menadione) dots; non-significant data are shown by yellow circles. Singletons have been removed from the network to reduce complexity.

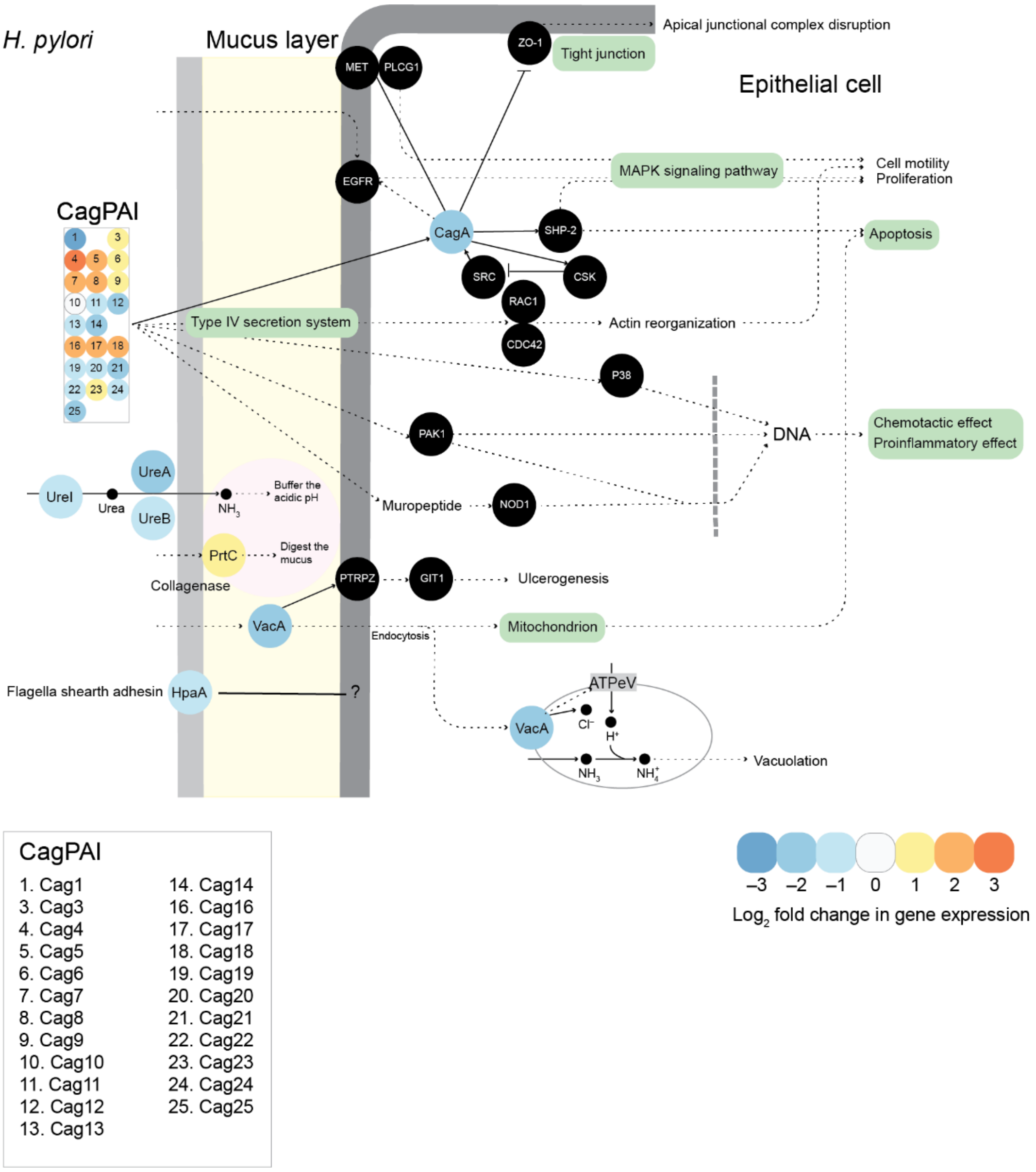


Supplementary Figure 3. Differential gene expression in the KEGG pathway "Epithelial cell signalling in *Helicobacter pylori* infection" in response to menadione (16 µM) treatment. This pathway, including the major virulence factor-encoding genes *vacA, cagA* and the genes of the *cag*PAI, was one of the most over-represented metabolic pathways associated with the genes that were significantly affected in menadione-treated *H. pylori* cells. Proposed mechanism of action is as follows. Down-regulation of *cagA* prevents inhibition of ZO-1, maintaining tight junction integrity in host epithelial cells, along with prevention activation of MAPK signalling. Down-regulation of *vacA* prevents or reduces ulcerogenesis, vacuolation, and apoptotic effects associated with *H. pylori* infection. Down-regulation of urease-associated genes limits *H. pylori's* ability to protect itself from host stomach acid. *H. pylori* gene products in the epithelial cell signalling pathway are shown as circles coloured by the $log_2$-fold change in expression levels of their genes in this study (refer to colour legend in figure). Black circles with white text represent host-associated gene products not assayed in this study but with which *H. pylori* gene products interact (note: not all host-associated gene products included in the pathway are shown to aid clarity). The pathway was created based on *H. pylori*-specific information available from KEGG on 3 September 2024.

Supplementary Table 1. Minimum inhibitory concentrations (MIC) and minimum bactericidal concentrations (MBC) of menadione against *H. pylori* strains

| *H. pylori* strain | MIC (mM) | MBC (mM) |
|---|---|---|
| 194A | 0.16 | 1.3 |
| 194C | 0.31 | 2.5 |
| 265A | 0.31 | 2.5 |
| 265C | 0.31 | 2.5 |
| 295A | 0.31 | 2.5 |
| 295C | 0.31 | 2.5 |
| 322A | 0.31 | not tested |
| 322C | 0.31 | not tested |
| 326A | 0.16 | 1.3 |
| 326C | 0.31 | 2.5 |
| 439A | 0.31 | 2.5 |
| 791A | 0.31 | not tested |
| 791C | 0.31 | not tested |

The number in the strain name refers to the patient while A indicates an isolate from the antrum and C indicates an isolate from the corpus of the patient's stomach. The menadione concentrations tested were 10, 5, 2.5, 1.25, 0.63, 0.31, 0.16, 0.08, 0.04, 0.02 mM alongside appropriate bacterial growth, sterility, and menadione negative controls. All strains and concentrations were tested in triplicate.

Supplementary Table 2. Outputs of DESeq2 analysis, used to identify significantly differentially expressed genes

| GeneID | Base mean | log2(FC) | StdErr | Wald-Stats | P-value | P-adj_BH | Effect of menadione on gene expression |
|---|---|---|---|---|---|---|---|
| 322A_00252 | 63601.15958 | -2.576364067 | 0.065474076 | -39.34937651 | 0 | 0 | Downregulated |
| 322A_01557 | 73521.61093 | -4.213895949 | 0.07005605 | -60.1503504 | 0 | 0 | Downregulated |
| 322A_01003 | 5207.160983 | -3.327696283 | 0.093703055 | -35.51321013 | 3.07E-276 | 1.62E-273 | Downregulated |
| 322A_00940 | 833.184363 | 3.236664217 | 0.094538753 | 34.23637522 | 6.96E-257 | 2.75E-254 | Upregulated |
| 322A_01163 | 237918.8957 | -4.440836085 | 0.131129806 | -33.86595476 | 2.11E-251 | 6.68E-249 | Downregulated |
| 322A_00270 | 2664.553627 | 4.945398544 | 0.146701974 | 33.71051115 | 4.05E-249 | 1.07E-246 | Upregulated |
| 322A_01444 | 3056.9371 | -2.673812004 | 0.082783664 | -32.29878779 | 7.27E-229 | 1.64E-226 | Downregulated |
| 322A_00276 | 21696.71056 | -2.126016368 | 0.067501867 | -31.49566778 | 9.96E-218 | 1.97E-215 | Downregulated |
| 322A_00054 | 1421.487531 | 2.156105251 | 0.069088724 | 31.20777356 | 8.36E-214 | 1.47E-211 | Upregulated |
| 322A_00300 | 6118.711479 | -3.023471057 | 0.097781691 | -30.92062575 | 6.31E-210 | 9.98E-208 | Downregulated |
| 322A_00936 | 2729.772532 | 2.379499767 | 0.078237575 | 30.41377181 | 3.61E-203 | 5.19E-201 | Upregulated |
| 322A_00578 | 3458.147463 | 2.109876319 | 0.069981147 | 30.14921031 | 1.10E-199 | 1.45E-197 | Upregulated |
| 322A_00778 | 142358.2895 | -4.865300471 | 0.162098274 | -30.01451121 | 6.35E-198 | 7.72E-196 | Downregulated |
| 322A_00548 | 715.8700253 | 3.251086464 | 0.109502754 | 29.68954066 | 1.05E-193 | 1.18E-191 | Upregulated |
| 322A_01004 | 757.1626693 | 3.695593966 | 0.12457055 | 29.66667461 | 2.07E-193 | 2.18E-191 | Upregulated |
| 322A_00818 | 4524.593955 | 3.883903317 | 0.132444985 | 29.3246537 | 5.03E-189 | 4.97E-187 | Upregulated |
| 322A_00981 | 18043.65346 | -1.930392412 | 0.066141585 | -29.18575979 | 2.94E-187 | 2.73E-185 | Downregulated |
| 322A_00463 | 13362.93074 | -4.359460204 | 0.150976978 | -28.87499972 | 2.46E-183 | 2.16E-181 | Downregulated |
| 322A_00762 | 190170.3878 | -4.750037503 | 0.165169473 | -28.75856784 | 7.08E-182 | 5.89E-180 | Downregulated |
| 322A_00390 | 4075849.764 | -4.295116805 | 0.149520924 | -28.72585786 | 1.81E-181 | 1.43E-179 | Downregulated |
| 322A_01080 | 4996.573116 | -2.758341351 | 0.096296385 | -28.64428766 | 1.89E-180 | 1.42E-178 | Downregulated |
| 322A_00741 | 854.2468688 | 4.303309867 | 0.152235194 | 28.26751003 | 8.67E-176 | 6.23E-174 | Upregulated |
| 322A_01062 | 2035.038049 | -1.537188994 | 0.05447191 | -28.21984746 | 3.34E-175 | 2.29E-173 | Downregulated |
| 322A_00231 | 4626.298569 | 2.04185817 | 0.072390489 | 28.20616614 | 4.91E-175 | 3.24E-173 | Upregulated |
| 322A_01340 | 3242.109129 | 4.277181464 | 0.151819082 | 28.1728845 | 1.26E-174 | 7.95E-173 | Upregulated |
| 322A_00272 | 7008.710278 | 3.934391203 | 0.140946459 | 27.91408325 | 1.80E-171 | 1.09E-169 | Upregulated |
| 322A_01247 | 2975.808229 | 2.239885923 | 0.080414029 | 27.85441741 | 9.52E-171 | 5.58E-169 | Upregulated |
| 322A_00234 | 3007.238096 | 2.789889416 | 0.100449818 | 27.77396174 | 8.95E-170 | 5.05E-168 | Upregulated |
| 322A_00271 | 1807.262175 | 4.813022192 | 0.174578692 | 27.56935652 | 2.59E-167 | 1.41E-165 | Upregulated |
| 322A_01366 | 59937.12042 | -2.693111445 | 0.098345321 | -27.38423573 | 4.23E-165 | 2.23E-163 | Downregulated |
| 322A_01283 | 755.4315269 | -1.919828771 | 0.070346511 | -27.29103059 | 5.42E-164 | 2.76E-162 | Downregulated |
| 322A_01184 | 5950.048842 | 2.890941455 | 0.106228682 | 27.21432101 | 4.40E-163 | 2.17E-161 | Upregulated |
| 322A_00462 | 15253.76914 | -3.835364232 | 0.142025139 | -27.00482646 | 1.30E-160 | 6.21E-159 | Downregulated |
| 322A_00354 | 1974.449013 | 2.921758362 | 0.108915263 | 26.82597716 | 1.61E-158 | 7.48E-157 | Upregulated |
| 322A_00740 | 1674.533172 | 4.451047098 | 0.166388083 | 26.750997 | 1.20E-157 | 5.43E-156 | Upregulated |
| 322A_00307 | 9930.315467 | 2.222785897 | 0.083164267 | 26.72765575 | 2.25E-157 | 9.86E-156 | Upregulated |
| 322A_01237 | 2191.752153 | 2.252801342 | 0.084679177 | 26.60395895 | 6.11E-156 | 2.61E-154 | Upregulated |
| 322A_01377 | 1350.813746 | 3.251402047 | 0.122987714 | 26.43680358 | 5.17E-154 | 2.15E-152 | Upregulated |

| GeneID | Base mean | log2(FC) | StdErr | Wald-Stats | P-value | P-adj_BH | Effect of menadione on gene expression |
|---|---|---|---|---|---|---|---|
| 322A_00002 | 2287.388259 | 2.954050126 | 0.112516869 | 26.25428655 | 6.39E-152 | 2.59E-150 | Upregulated |
| 322A_00457 | 3378.461848 | 2.988783303 | 0.114609116 | 26.07805909 | 6.47E-150 | 2.56E-148 | Upregulated |
| 322A_00594 | 6863.63242 | -4.405130659 | 0.169350869 | -26.0118575 | 3.64E-149 | 1.40E-147 | Downregulated |
| 322A_01011 | 1261.752854 | 2.314757708 | 0.089056889 | 25.99189941 | 6.11E-149 | 2.30E-147 | Upregulated |
| 322A_01081 | 26502.52314 | -3.269880224 | 0.126517748 | -25.84522953 | 2.75E-147 | 1.01E-145 | Downregulated |
| 322A_00295 | 1330.443356 | 2.828774821 | 0.110066888 | 25.70050686 | 1.15E-145 | 4.15E-144 | Upregulated |
| 322A_00481 | 1375.164867 | 3.700135141 | 0.145549313 | 25.42186607 | 1.45E-142 | 5.08E-141 | Upregulated |
| 322A_01363 | 14301.60148 | -3.289095619 | 0.129969812 | -25.30661205 | 2.70E-141 | 9.28E-140 | Downregulated |
| 322A_01060 | 2361.317947 | 3.253070077 | 0.128587565 | 25.29848108 | 3.32E-141 | 1.12E-139 | Upregulated |
| 322A_00408 | 4652.039864 | 1.946540898 | 0.077070722 | 25.25655447 | 9.60E-141 | 3.16E-139 | Upregulated |
| 322A_00076 | 9255.979234 | -2.324233315 | 0.092954984 | -25.00385914 | 5.55E-138 | 1.79E-136 | Downregulated |
| 322A_00586 | 1603.386652 | 3.83527181 | 0.153526175 | 24.98122426 | 9.78E-138 | 3.09E-136 | Upregulated |
| 322A_01375 | 2434.707494 | 2.586190722 | 0.104435611 | 24.76349484 | 2.22E-135 | 6.88E-134 | Upregulated |
| 322A_00746 | 15198.63117 | -2.044480415 | 0.082572673 | -24.7597703 | 2.43E-135 | 7.40E-134 | Downregulated |
| 322A_01561 | 6227.390075 | 2.739278783 | 0.111190607 | 24.63588298 | 5.21E-134 | 1.56E-132 | Upregulated |
| 322A_00717 | 1217.109319 | 3.284824268 | 0.134646969 | 24.39582778 | 1.89E-131 | 5.54E-130 | Upregulated |
| 322A_00075 | 6715.990418 | -2.369747633 | 0.097970447 | -24.1883925 | 2.95E-129 | 8.47E-128 | Downregulated |
| 322A_01131 | 1916.42702 | 2.746218274 | 0.113581807 | 24.17832868 | 3.76E-129 | 1.06E-127 | Upregulated |
| 322A_01173 | 11017.65579 | -2.115888167 | 0.08782172 | -24.09299406 | 2.96E-128 | 8.21E-127 | Downregulated |
| 322A_00902 | 8288.716161 | 2.11490432 | 0.087909609 | 24.05771497 | 6.93E-128 | 1.89E-126 | Upregulated |
| 322A_00804 | 10986.17979 | -1.422714913 | 0.05960539 | -23.86889699 | 6.45E-126 | 1.73E-124 | Downregulated |
| 322A_00663 | 502.7030408 | 3.319132265 | 0.140886632 | 23.55888721 | 1.02E-122 | 2.68E-121 | Upregulated |
| 322A_00706 | 2798.462144 | 1.837216467 | 0.078072587 | 23.53215825 | 1.91E-122 | 4.96E-121 | Upregulated |
| 322A_00281 | 2111.800734 | 1.840233053 | 0.078822357 | 23.34658735 | 1.49E-120 | 3.81E-119 | Upregulated |
| 322A_00591 | 2705.149327 | 2.16825474 | 0.093075776 | 23.29558603 | 4.91E-120 | 1.23E-118 | Upregulated |
| 322A_00873 | 352.3614382 | 4.083610942 | 0.177473001 | 23.00975881 | 3.72E-117 | 9.20E-116 | Upregulated |
| 322A_00602 | 2268.521918 | 2.96650393 | 0.129409807 | 22.92333174 | 2.72E-116 | 6.61E-115 | Upregulated |
| 322A_00243 | 716.0998887 | 3.152285061 | 0.137653866 | 22.90008379 | 4.64E-116 | 1.11E-114 | Upregulated |
| 322A_00634 | 393.7102555 | 3.410710993 | 0.149561682 | 22.80471142 | 4.12E-115 | 9.72E-114 | Upregulated |
| 322A_00335 | 2801.099195 | 2.830762941 | 0.124566219 | 22.72496482 | 2.54E-114 | 5.90E-113 | Upregulated |
| 322A_00802 | 751.7032919 | 3.349553613 | 0.147442899 | 22.71763269 | 3.00E-114 | 6.87E-113 | Upregulated |
| 322A_00988 | 2317.147972 | 3.376205352 | 0.148633733 | 22.71493336 | 3.19E-114 | 7.20E-113 | Upregulated |
| 322A_00677 | 1176.337155 | 3.291013651 | 0.145140348 | 22.67469869 | 7.96E-114 | 1.77E-112 | Upregulated |
| 322A_00658 | 4311.215717 | 1.702837364 | 0.075414534 | 22.57969761 | 6.86E-113 | 1.51E-111 | Upregulated |
| 322A_01396 | 2441.300495 | -2.441648546 | 0.108270425 | -22.55138961 | 1.30E-112 | 2.82E-111 | Downregulated |
| 322A_00771 | 5530.336012 | -1.574039021 | 0.069908767 | -22.51561698 | 2.92E-112 | 6.24E-111 | Downregulated |
| 322A_01519 | 939722.115 | -6.911264272 | 0.307500417 | -22.47562568 | 7.19E-112 | 1.52E-110 | Downregulated |
| 322A_00073 | 3773.256265 | -1.619016466 | 0.072486923 | -22.33529023 | 1.68E-110 | 3.49E-109 | Downregulated |
| 322A_00760 | 1000.412476 | 1.876573749 | 0.08455891 | 22.19250173 | 4.06E-109 | 8.33E-108 | Upregulated |
| 322A_01059 | 1236.828677 | 4.416702934 | 0.199701159 | 22.1165614 | 2.19E-108 | 4.44E-107 | Upregulated |
| 322A_00145 | 8551.374363 | -2.314128609 | 0.104645642 | -22.11395113 | 2.32E-108 | 4.59E-107 | Downregulated |
| 322A_00823 | 17231.91826 | -3.513804004 | 0.15889297 | -22.11428233 | 2.30E-108 | 4.59E-107 | Downregulated |

| GeneID | Base mean | log2(FC) | StdErr | Wald-Stats | P-value | P-adj_BH | Effect of menadione on gene expression |
|---|---|---|---|---|---|---|---|
| 322A_00441 | 4906.379681 | 1.774430953 | 0.080457854 | 22.05416699 | 8.71E-108 | 1.68E-106 | Upregulated |
| 322A_00549 | 1144.493924 | 3.886689843 | 0.17623386 | 22.05416053 | 8.71E-108 | 1.68E-106 | Upregulated |
| 322A_01605 | 51290.93518 | -3.024084436 | 0.137160515 | -22.04777697 | 1.00E-107 | 1.91E-106 | Downregulated |
| 322A_01354 | 13871.00656 | 1.471822993 | 0.067020859 | 21.96067033 | 6.85E-107 | 1.29E-105 | Upregulated |
| 322A_00563 | 2684.419869 | 2.57856587 | 0.117864505 | 21.87737404 | 4.27E-106 | 7.94E-105 | Upregulated |
| 322A_00901 | 725.9137768 | 4.388724889 | 0.200672208 | 21.8701181 | 5.00E-106 | 9.20E-105 | Upregulated |
| 322A_00709 | 1237.83351 | 2.989794675 | 0.136923844 | 21.83545683 | 1.07E-105 | 1.94E-104 | Upregulated |
| 322A_01455 | 11744.81155 | -1.603336559 | 0.073538691 | -21.80262562 | 2.19E-105 | 3.94E-104 | Downregulated |
| 322A_00900 | 1474.516317 | 3.241737387 | 0.148875359 | 21.77484171 | 4.02E-105 | 7.14E-104 | Upregulated |
| 322A_01157 | 6631.134784 | 2.147166284 | 0.098795184 | 21.73351157 | 9.89E-105 | 1.74E-103 | Upregulated |
| 322A_01239 | 2917.631066 | 1.739384094 | 0.080698748 | 21.55404065 | 4.85E-103 | 8.43E-102 | Upregulated |
| 322A_00233 | 2819.888881 | 1.78092077 | 0.082832284 | 21.50032179 | 1.55E-102 | 2.66E-101 | Upregulated |
| 322A_00951 | 4336.215664 | -2.096339919 | 0.097596918 | -21.47957078 | 2.42E-102 | 4.11E-101 | Downregulated |
| 322A_00831 | 702.5436224 | 2.650323136 | 0.124270205 | 21.32710042 | 6.36E-101 | 1.07E-99 | Upregulated |
| 322A_01556 | 44973.61404 | -3.237470134 | 0.152181378 | -21.27376009 | 1.99E-100 | 3.31E-99 | Downregulated |
| 322A_00083 | 3140.075687 | 2.874595242 | 0.135233645 | 21.2565094 | 2.87E-100 | 4.73E-99 | Upregulated |
| 322A_01278 | 225.5728397 | -2.662994225 | 0.125381271 | -21.23917074 | 4.15E-100 | 6.77E-99 | Downregulated |
| 322A_00127 | 1216.991294 | 2.278203111 | 0.107384501 | 21.21538108 | 6.89E-100 | 1.11E-98 | Upregulated |
| 322A_00522 | 1149.341295 | 3.79385034 | 0.179501068 | 21.13553078 | 3.75E-99 | 5.99E-98 | Upregulated |
| 322A_01187 | 1407.104958 | 2.184604311 | 0.103645327 | 21.07769238 | 1.27E-98 | 2.01E-97 | Upregulated |
| 322A_00824 | 8431.822836 | -4.006201827 | 0.190439183 | -21.03664682 | 3.03E-98 | 4.74E-97 | Downregulated |
| 322A_00562 | 3454.667044 | 2.53776825 | 0.120785374 | 21.0105592 | 5.25E-98 | 8.14E-97 | Upregulated |
| 322A_01238 | 3476.252859 | 1.49669472 | 0.071638668 | 20.89227466 | 6.29E-97 | 9.66E-96 | Upregulated |
| 322A_00025 | 6212.110973 | -3.029983177 | 0.145224551 | -20.86412495 | 1.13E-96 | 1.72E-95 | Downregulated |
| 322A_00491 | 3043.627478 | -3.18319927 | 0.152840787 | -20.82689667 | 2.47E-96 | 3.72E-95 | Downregulated |
| 322A_01367 | 5430.571836 | 3.159590973 | 0.151975721 | 20.79010347 | 5.32E-96 | 7.93E-95 | Upregulated |
| 322A_01172 | 6206.310659 | -2.084739863 | 0.100669086 | -20.7088387 | 2.88E-95 | 4.26E-94 | Downregulated |
| 322A_01251 | 1152.422182 | 1.719441838 | 0.083031743 | 20.70824698 | 2.92E-95 | 4.27E-94 | Upregulated |
| 322A_00573 | 402.5842176 | 3.198670183 | 0.155000108 | 20.63656745 | 1.29E-94 | 1.87E-93 | Upregulated |
| 322A_00107 | 5450.943542 | -1.254525815 | 0.0609727 | -20.57520512 | 4.58E-94 | 6.58E-93 | Downregulated |
| 322A_00575 | 4193.396325 | 2.129478439 | 0.104605929 | 20.35714867 | 4.01E-92 | 5.72E-91 | Upregulated |
| 322A_01013 | 10288.40957 | -3.10040669 | 0.15247082 | -20.33442653 | 6.38E-92 | 9.00E-91 | Downregulated |
| 322A_00618 | 5409.83948 | 2.627422079 | 0.129634799 | 20.26787633 | 2.47E-91 | 3.46E-90 | Upregulated |
| 322A_01406 | 1687.384686 | 2.478017025 | 0.122319329 | 20.25858903 | 2.98E-91 | 4.14E-90 | Upregulated |
| 322A_00790 | 19493.64622 | -2.264576192 | 0.112717436 | -20.09073542 | 8.89E-90 | 1.22E-88 | Downregulated |
| 322A_00605 | 4681.304076 | 2.486072074 | 0.124156561 | 20.0236866 | 3.42E-89 | 4.67E-88 | Upregulated |
| 322A_00963 | 7426.047073 | 0.94465489 | 0.047512114 | 19.88240078 | 5.78E-88 | 7.81E-87 | Upregulated |
| 322A_00523 | 2617.931599 | 3.24414126 | 0.163256199 | 19.87147367 | 7.19E-88 | 9.63E-87 | Upregulated |
| 322A_01508 | 3435.683944 | 2.334741501 | 0.117714412 | 19.8339477 | 1.52E-87 | 2.01E-86 | Upregulated |
| 322A_00832 | 1498.210638 | 2.970348286 | 0.150797086 | 19.69765048 | 2.26E-86 | 2.98E-85 | Upregulated |
| 322A_01155 | 3137.977429 | 2.312128837 | 0.117694876 | 19.64511049 | 6.37E-86 | 8.32E-85 | Upregulated |
| 322A_00830 | 2235.96544 | 2.818740136 | 0.144009728 | 19.57326196 | 2.61E-85 | 3.39E-84 | Upregulated |

| GeneID | Base mean | log2(FC) | StdErr | Wald-Stats | P-value | P-adj_BH | Effect of menadione on gene expression |
|---|---|---|---|---|---|---|---|
| 322A_00174 | 14853.36954 | -3.471463354 | 0.177999545 | -19.50265297 | 1.04E-84 | 1.34E-83 | Downregulated |
| 322A_00358 | 556.3957528 | 1.900013029 | 0.098406595 | 19.30778144 | 4.62E-83 | 5.89E-82 | Upregulated |
| 322A_00613 | 10005.78559 | -1.980084997 | 0.102652716 | -19.28916323 | 6.62E-83 | 8.38E-82 | Downregulated |
| 322A_01547 | 11089.07397 | 2.58030806 | 0.134170946 | 19.23149638 | 2.02E-82 | 2.53E-81 | Upregulated |
| 322A_00404 | 665.1964718 | 3.824797412 | 0.199106251 | 19.2098309 | 3.06E-82 | 3.81E-81 | Upregulated |
| 322A_01135 | 7353.262879 | 1.134763749 | 0.059222505 | 19.16102258 | 7.83E-82 | 9.67E-81 | Upregulated |
| 322A_00467 | 5241.30268 | -1.225174893 | 0.06397849 | -19.14979388 | 9.72E-82 | 1.19E-80 | Downregulated |
| 322A_01170 | 1206.644414 | -1.88711776 | 0.099315333 | -19.00127296 | 1.66E-80 | 2.02E-79 | Downregulated |
| 322A_00570 | 25431.00386 | -2.544420203 | 0.133974378 | -18.99184183 | 1.99E-80 | 2.40E-79 | Downregulated |
| 322A_00385 | 1444.597352 | 1.530209591 | 0.080741665 | 18.9519202 | 4.26E-80 | 5.10E-79 | Upregulated |
| 322A_00890 | 3288.298592 | 2.073750089 | 0.109890292 | 18.87109447 | 1.97E-79 | 2.34E-78 | Upregulated |
| 322A_00606 | 2387.170246 | 1.698340603 | 0.090034465 | 18.86322757 | 2.29E-79 | 2.70E-78 | Upregulated |
| 322A_01494 | 320.3292619 | 5.484281528 | 0.291127068 | 18.83810245 | 3.68E-79 | 4.31E-78 | Upregulated |
| 322A_00041 | 330.312684 | -2.199786336 | 0.116778579 | -18.83724187 | 3.74E-79 | 4.35E-78 | Downregulated |
| 322A_01234 | 6231.209928 | 1.428218502 | 0.075852285 | 18.82894502 | 4.37E-79 | 5.05E-78 | Upregulated |
| 322A_00151 | 2324.421536 | 3.309967953 | 0.176391367 | 18.764909 | 1.46E-78 | 1.68E-77 | Upregulated |
| 322A_00116 | 2111.182118 | 2.132738687 | 0.1140969 | 18.69234555 | 5.71E-78 | 6.45E-77 | Upregulated |
| 322A_00494 | 24524.75452 | 1.003771026 | 0.05369961 | 18.69233346 | 5.72E-78 | 6.45E-77 | Upregulated |
| 322A_01318 | 6037.781488 | -1.709347774 | 0.091804268 | -18.61948038 | 2.23E-77 | 2.50E-76 | Downregulated |
| 322A_00707 | 4705.642955 | 1.789454366 | 0.096183261 | 18.60463396 | 2.95E-77 | 3.28E-76 | Upregulated |
| 322A_00632 | 353.3083514 | 3.140901555 | 0.169595968 | 18.51990696 | 1.43E-76 | 1.58E-75 | Upregulated |
| 322A_00726 | 1294.345047 | 2.428666091 | 0.131468348 | 18.47339022 | 3.38E-76 | 3.71E-75 | Upregulated |
| 322A_00241 | 3619.176436 | 2.019684735 | 0.109633225 | 18.42219571 | 8.72E-76 | 9.51E-75 | Upregulated |
| 322A_01548 | 4714.380963 | 3.165441907 | 0.173185831 | 18.27771869 | 1.25E-74 | 1.35E-73 | Upregulated |
| 322A_01576 | 1177.610223 | 2.670276271 | 0.146209833 | 18.2633152 | 1.62E-74 | 1.74E-73 | Upregulated |
| 322A_00317 | 244.6216466 | 5.075216115 | 0.278576047 | 18.21842249 | 3.69E-74 | 3.94E-73 | Upregulated |
| 322A_00387 | 2543.177429 | 1.763663561 | 0.096956985 | 18.19016515 | 6.18E-74 | 6.55E-73 | Upregulated |
| 322A_00273 | 1384.698176 | 4.313517399 | 0.237551637 | 18.15823056 | 1.11E-73 | 1.16E-72 | Upregulated |
| 322A_01296 | 1505.551664 | 1.695700743 | 0.093385009 | 18.15816863 | 1.11E-73 | 1.16E-72 | Upregulated |
| 322A_01451 | 6192.905059 | -2.198487043 | 0.12134204 | -18.11809851 | 2.29E-73 | 2.39E-72 | Downregulated |
| 322A_01223 | 6914.196099 | -1.581007103 | 0.087329649 | -18.10389845 | 2.97E-73 | 3.07E-72 | Downregulated |
| 322A_00287 | 15994.13462 | -1.879420744 | 0.103846665 | -18.09803663 | 3.30E-73 | 3.39E-72 | Downregulated |
| 322A_01486 | 49630.37868 | -1.745027722 | 0.096645276 | -18.05600637 | 7.08E-73 | 7.22E-72 | Downregulated |
| 322A_00479 | 8616.872286 | -1.274572273 | 0.070768222 | -18.01051711 | 1.61E-72 | 1.63E-71 | Downregulated |
| 322A_00725 | 1092.707101 | 2.938281749 | 0.164236271 | 17.89057753 | 1.40E-71 | 1.41E-70 | Upregulated |
| 322A_00118 | 471.8580375 | 3.521867356 | 0.196885173 | 17.88792575 | 1.46E-71 | 1.47E-70 | Upregulated |
| 322A_00067 | 484.6503499 | 3.303239101 | 0.184924579 | 17.86262873 | 2.31E-71 | 2.29E-70 | Upregulated |
| 322A_00520 | 3497.567099 | -1.415023895 | 0.080025592 | -17.68214209 | 5.76E-70 | 5.69E-69 | Downregulated |
| 322A_00819 | 4257.75576 | 1.612351605 | 0.091229861 | 17.67350722 | 6.71E-70 | 6.59E-69 | Upregulated |
| 322A_00909 | 2911.939681 | 1.957098718 | 0.110912076 | 17.6454971 | 1.10E-69 | 1.08E-68 | Upregulated |
| 322A_00455 | 938.1871699 | 3.355185376 | 0.190167021 | 17.64336088 | 1.14E-69 | 1.11E-68 | Upregulated |
| 322A_01036 | 1982.727105 | -1.53882539 | 0.087317374 | -17.62335859 | 1.63E-69 | 1.57E-68 | Downregulated |

| GeneID | Base mean | log2(FC) | StdErr | Wald-Stats | P-value | P-adj_BH | Effect of menadione on gene expression |
|---|---|---|---|---|---|---|---|
| 322A_00279 | 1943.858451 | -1.405888275 | 0.079842294 | -17.60831521 | 2.13E-69 | 2.04E-68 | Downregulated |
| 322A_01425 | 9930.992848 | 1.553460859 | 0.088434309 | 17.56626896 | 4.47E-69 | 4.25E-68 | Upregulated |
| 322A_00744 | 1081.727719 | 2.624449607 | 0.149687339 | 17.53287637 | 8.04E-69 | 7.61E-68 | Upregulated |
| 322A_01136 | 8297.177404 | -2.174219454 | 0.124122617 | -17.51670656 | 1.07E-68 | 1.01E-67 | Downregulated |
| 322A_00982 | 5623.791344 | -2.005449708 | 0.114811513 | -17.46732233 | 2.54E-68 | 2.38E-67 | Downregulated |
| 322A_00694 | 46100.5606 | -3.047613441 | 0.174628306 | -17.45200137 | 3.32E-68 | 3.09E-67 | Downregulated |
| 322A_00660 | 4008.976192 | -1.614920656 | 0.092747073 | -17.4120929 | 6.68E-68 | 6.18E-67 | Downregulated |
| 322A_00668 | 2152.398031 | 1.913325876 | 0.109951532 | 17.40153908 | 8.03E-68 | 7.38E-67 | Upregulated |
| 322A_00943 | 3336.100985 | 2.723737671 | 0.156628314 | 17.38981665 | 9.85E-68 | 9.01E-67 | Upregulated |
| 322A_01178 | 2994.650083 | 1.646333102 | 0.094744311 | 17.37659054 | 1.24E-67 | 1.13E-66 | Upregulated |
| 322A_00047 | 436.6983127 | -2.705254338 | 0.155693373 | -17.37552653 | 1.26E-67 | 1.14E-66 | Downregulated |
| 322A_01229 | 336.5924717 | 2.34897599 | 0.135266839 | 17.36549775 | 1.51E-67 | 1.35E-66 | Upregulated |
| 322A_00133 | 1070272.333 | -4.117909884 | 0.237425859 | -17.34398223 | 2.19E-67 | 1.96E-66 | Downregulated |
| 322A_01551 | 117678.6557 | -3.068889021 | 0.177004314 | -17.33793352 | 2.43E-67 | 2.16E-66 | Downregulated |
| 322A_00633 | 227.8619319 | 3.445897741 | 0.198953833 | 17.3200872 | 3.32E-67 | 2.93E-66 | Upregulated |
| 322A_00336 | 5615.339419 | 1.939042506 | 0.112469462 | 17.24061335 | 1.32E-66 | 1.16E-65 | Upregulated |
| 322A_00650 | 340.2586775 | 3.591536915 | 0.208377828 | 17.23569606 | 1.43E-66 | 1.25E-65 | Upregulated |
| 322A_00516 | 6035.296516 | -3.405881581 | 0.197699426 | -17.22757446 | 1.65E-66 | 1.43E-65 | Downregulated |
| 322A_01563 | 24445.48368 | -2.230267246 | 0.129570267 | -17.21280125 | 2.13E-66 | 1.84E-65 | Downregulated |
| 322A_00738 | 1715.414107 | 1.926718101 | 0.112541349 | 17.12009076 | 1.05E-65 | 9.03E-65 | Upregulated |
| 322A_00791 | 1142.331978 | 2.060909256 | 0.120838175 | 17.0551173 | 3.20E-65 | 2.74E-64 | Upregulated |
| 322A_01076 | 11461.61322 | -2.80621902 | 0.164607732 | -17.04791743 | 3.62E-65 | 3.08E-64 | Downregulated |
| 322A_01308 | 17022.1986 | -1.902938281 | 0.11163528 | -17.04602948 | 3.74E-65 | 3.16E-64 | Downregulated |
| 322A_00724 | 416.2642815 | 3.294698161 | 0.19399273 | 16.98361664 | 1.09E-64 | 9.13E-64 | Upregulated |
| 322A_00743 | 3600.127859 | 1.628841813 | 0.096199183 | 16.93197141 | 2.61E-64 | 2.19E-63 | Upregulated |
| 322A_00526 | 1046.956218 | 2.499360693 | 0.147930604 | 16.8954944 | 4.86E-64 | 4.04E-63 | Upregulated |
| 322A_00187 | 3364.075278 | 1.550865651 | 0.091798114 | 16.89430833 | 4.95E-64 | 4.10E-63 | Upregulated |
| 322A_00228 | 1861.134894 | -1.440870528 | 0.085415967 | -16.86886625 | 7.62E-64 | 6.28E-63 | Downregulated |
| 322A_00567 | 3449.724422 | -2.023353274 | 0.120164052 | -16.83825768 | 1.28E-63 | 1.05E-62 | Downregulated |
| 322A_00941 | 457.6981232 | 2.724461157 | 0.162251416 | 16.79160178 | 2.81E-63 | 2.29E-62 | Upregulated |
| 322A_00496 | 15219.21748 | 1.271720162 | 0.075979562 | 16.7376611 | 6.97E-63 | 5.65E-62 | Upregulated |
| 322A_01352 | 70228.04991 | -2.69694171 | 0.161385731 | -16.71115347 | 1.09E-62 | 8.77E-62 | Downregulated |
| 322A_00980 | 34554.0491 | -2.444897585 | 0.146777878 | -16.6571258 | 2.69E-62 | 2.16E-61 | Downregulated |
| 322A_00136 | 4849.361158 | -2.113251502 | 0.127367862 | -16.59171685 | 8.00E-62 | 6.39E-61 | Downregulated |
| 322A_01159 | 627.3888194 | 3.1363349 | 0.190107852 | 16.49766102 | 3.81E-61 | 3.03E-60 | Upregulated |
| 322A_00394 | 2536.464356 | 1.729121261 | 0.104894881 | 16.48432446 | 4.76E-61 | 3.76E-60 | Upregulated |
| 322A_01106 | 1817.536781 | 1.449043027 | 0.088011718 | 16.46420564 | 6.63E-61 | 5.22E-60 | Upregulated |
| 322A_01186 | 13079.30177 | 2.538837323 | 0.154552979 | 16.42697111 | 1.23E-60 | 9.60E-60 | Upregulated |
| 322A_01075 | 2730.057299 | 1.522399247 | 0.092782698 | 16.40822347 | 1.67E-60 | 1.30E-59 | Upregulated |
| 322A_01427 | 2272.212763 | 2.322489768 | 0.141706347 | 16.38945474 | 2.27E-60 | 1.76E-59 | Upregulated |
| 322A_00482 | 592.8823423 | 1.657972811 | 0.101280307 | 16.3701401 | 3.12E-60 | 2.41E-59 | Upregulated |
| 322A_00359 | 2497.112723 | 1.78396693 | 0.108986293 | 16.36872752 | 3.20E-60 | 2.45E-59 | Upregulated |

| GeneID | Base mean | log2(FC) | StdErr | Wald-Stats | P-value | P-adj_BH | Effect of menadione on gene expression |
|---|---|---|---|---|---|---|---|
| 322A_01577 | 2425.0366 | 2.038283089 | 0.124761579 | 16.33742625 | 5.35E-60 | 4.08E-59 | Upregulated |
| 322A_00949 | 65972.45331 | 2.707597919 | 0.165861818 | 16.32441963 | 6.62E-60 | 5.03E-59 | Upregulated |
| 322A_00333 | 519.5834131 | 3.223945424 | 0.19777657 | 16.30094717 | 9.72E-60 | 7.35E-59 | Upregulated |
| 322A_01595 | 9323.295167 | -2.453121702 | 0.151131495 | -16.2317041 | 3.01E-59 | 2.27E-58 | Downregulated |
| 322A_00932 | 19174.65645 | -2.042435901 | 0.126455412 | -16.15143129 | 1.11E-58 | 8.31E-58 | Downregulated |
| 322A_01293 | 1614.914627 | -1.699501459 | 0.105238497 | -16.14904726 | 1.15E-58 | 8.60E-58 | Downregulated |
| 322A_00454 | 2499.901329 | 3.153185529 | 0.195731577 | 16.10974364 | 2.18E-58 | 1.62E-57 | Upregulated |
| 322A_00968 | 2995.002005 | -2.990736674 | 0.185934316 | -16.08490967 | 3.26E-58 | 2.40E-57 | Downregulated |
| 322A_00603 | 1106.698518 | 2.017299891 | 0.125472198 | 16.07766444 | 3.66E-58 | 2.69E-57 | Upregulated |
| 322A_00964 | 1717.712948 | 2.210568064 | 0.138120631 | 16.0046189 | 1.19E-57 | 8.68E-57 | Upregulated |
| 322A_00264 | 17397.1668 | -2.55454377 | 0.160150639 | -15.95088087 | 2.81E-57 | 2.05E-56 | Downregulated |
| 322A_01177 | 624.8934442 | 3.361169238 | 0.2109727 | 15.93177333 | 3.81E-57 | 2.77E-56 | Upregulated |
| 322A_00159 | 875.0427075 | 1.919698259 | 0.120782289 | 15.8938721 | 6.99E-57 | 5.04E-56 | Upregulated |
| 322A_00238 | 1091.367572 | 1.254680199 | 0.079373131 | 15.80736681 | 2.77E-56 | 1.99E-55 | Upregulated |
| 322A_00827 | 26271.29912 | -2.785191519 | 0.176868078 | -15.7472821 | 7.17E-56 | 5.13E-55 | Downregulated |
| 322A_00944 | 8789.741893 | 1.322878087 | 0.084248292 | 15.7021354 | 1.46E-55 | 1.04E-54 | Upregulated |
| 322A_01610 | 12942.51541 | -2.828202714 | 0.180282807 | -15.68758976 | 1.84E-55 | 1.30E-54 | Downregulated |
| 322A_00639 | 54481.59649 | -1.743109504 | 0.111231316 | -15.67103186 | 2.39E-55 | 1.68E-54 | Downregulated |
| 322A_00525 | 243.9646409 | 3.657424345 | 0.23358105 | 15.65805252 | 2.93E-55 | 2.06E-54 | Upregulated |
| 322A_01086 | 18026.39344 | -1.001431408 | 0.064264012 | -15.58308256 | 9.49E-55 | 6.64E-54 | Downregulated |
| 322A_00617 | 450.5010189 | 3.588672695 | 0.230970099 | 15.53739084 | 1.94E-54 | 1.35E-53 | Upregulated |
| 322A_01252 | 995.9008727 | 1.62453361 | 0.104604509 | 15.53024457 | 2.17E-54 | 1.50E-53 | Upregulated |
| 322A_00583 | 4559.61851 | 1.739790039 | 0.112114994 | 15.51790686 | 2.62E-54 | 1.81E-53 | Upregulated |
| 322A_00290 | 2821.104596 | 1.667959107 | 0.107673731 | 15.49086383 | 4.00E-54 | 2.75E-53 | Upregulated |
| 322A_01300 | 11028.71556 | -1.026237565 | 0.066364687 | -15.46360887 | 6.11E-54 | 4.18E-53 | Downregulated |
| 322A_00898 | 4355.907231 | 0.756124148 | 0.048917967 | 15.45698216 | 6.77E-54 | 4.61E-53 | Upregulated |
| 322A_00776 | 256.5836026 | 2.714067891 | 0.175784888 | 15.43971112 | 8.85E-54 | 6.00E-53 | Upregulated |
| 322A_00934 | 885.3057094 | 1.867600477 | 0.121711839 | 15.34444377 | 3.86E-53 | 2.61E-52 | Upregulated |
| 322A_01311 | 8676.590971 | 1.487066333 | 0.097156921 | 15.30581982 | 6.99E-53 | 4.70E-52 | Upregulated |
| 322A_01066 | 54078.56149 | -3.33200666 | 0.217719665 | -15.30411439 | 7.18E-53 | 4.81E-52 | Downregulated |
| 322A_00301 | 55965.73864 | -2.649477482 | 0.173128774 | -15.30350744 | 7.24E-53 | 4.83E-52 | Downregulated |
| 322A_01241 | 2845.397468 | 2.182494047 | 0.142831564 | 15.28019429 | 1.04E-52 | 6.88E-52 | Upregulated |
| 322A_01009 | 766.3745422 | 1.52156847 | 0.099740553 | 15.25526403 | 1.52E-52 | 1.00E-51 | Upregulated |
| 322A_01048 | 733.9852702 | 1.588243419 | 0.104277356 | 15.23095219 | 2.20E-52 | 1.45E-51 | Upregulated |
| 322A_01332 | 4983.774862 | -2.74634768 | 0.180493334 | -15.21578451 | 2.78E-52 | 1.82E-51 | Downregulated |
| 322A_00703 | 6857.504625 | -0.982223942 | 0.064573689 | -15.21090036 | 2.99E-52 | 1.96E-51 | Downregulated |
| 322A_01051 | 4028.308399 | -1.551480865 | 0.102085371 | -15.19787648 | 3.65E-52 | 2.38E-51 | Downregulated |
| 322A_01198 | 13789.80677 | -3.200040303 | 0.210765371 | -15.18295103 | 4.59E-52 | 2.97E-51 | Downregulated |
| 322A_01220 | 3609.109592 | 1.329269314 | 0.08795786 | 15.11256998 | 1.34E-51 | 8.64E-51 | Upregulated |
| 322A_00314 | 7866.797519 | 1.051099939 | 0.069561696 | 15.11032641 | 1.38E-51 | 8.90E-51 | Upregulated |
| 322A_01456 | 79577.98371 | -3.008583729 | 0.199402179 | -15.08801834 | 1.94E-51 | 1.24E-50 | Downregulated |
| 322A_00319 | 3605.134501 | 1.561618992 | 0.103692995 | 15.06002398 | 2.97E-51 | 1.89E-50 | Upregulated |

| GeneID | Base mean | log2(FC) | StdErr | Wald-Stats | P-value | P-adj_BH | Effect of menadione on gene expression |
|---|---|---|---|---|---|---|---|
| 322A_00905 | 1953.181429 | -2.786599176 | 0.185279762 | -15.03995444 | 4.02E-51 | 2.55E-50 | Downregulated |
| 322A_00664 | 3419.745031 | 2.215855589 | 0.147684016 | 15.00403122 | 6.91E-51 | 4.37E-50 | Upregulated |
| 322A_00117 | 835.5939516 | 2.193751481 | 0.146619647 | 14.96219316 | 1.30E-50 | 8.17E-50 | Upregulated |
| 322A_00169 | 2360.115524 | 1.280685563 | 0.085952889 | 14.89985481 | 3.30E-50 | 2.07E-49 | Upregulated |
| 322A_00193 | 1123.290074 | 2.230934212 | 0.149958335 | 14.87702714 | 4.65E-50 | 2.90E-49 | Upregulated |
| 322A_01515 | 1992.475818 | 2.157257749 | 0.145164016 | 14.86082993 | 5.92E-50 | 3.68E-49 | Upregulated |
| 322A_00917 | 27629.95859 | -1.549017284 | 0.104524438 | -14.81966621 | 1.09E-49 | 6.78E-49 | Downregulated |
| 322A_01333 | 32618.04991 | -3.042147729 | 0.205502159 | -14.80348306 | 1.39E-49 | 8.59E-49 | Downregulated |
| 322A_00391 | 4263.325469 | -1.599895411 | 0.10887037 | -14.6954163 | 6.90E-49 | 4.24E-48 | Downregulated |
| 322A_00517 | 4040.638713 | 1.725053542 | 0.117460149 | 14.68628777 | 7.89E-49 | 4.84E-48 | Upregulated |
| 322A_01243 | 916.6733968 | 2.49277697 | 0.169937864 | 14.66875547 | 1.02E-48 | 6.24E-48 | Upregulated |
| 322A_00115 | 35258.38821 | -2.198815165 | 0.149909939 | -14.6675743 | 1.04E-48 | 6.32E-48 | Downregulated |
| 322A_00235 | 178.0750697 | 2.230214608 | 0.152085349 | 14.66423049 | 1.09E-48 | 6.62E-48 | Upregulated |
| 322A_00406 | 2062.624696 | -0.777012855 | 0.053046447 | -14.64778316 | 1.39E-48 | 8.40E-48 | Downregulated |
| 322A_01202 | 1248.122863 | -1.135166732 | 0.07761371 | -14.62585333 | 1.92E-48 | 1.16E-47 | Downregulated |
| 322A_00222 | 1348.061097 | 4.482854883 | 0.306694324 | 14.61668684 | 2.20E-48 | 1.32E-47 | Upregulated |
| 322A_01295 | 2440.508071 | 2.804883898 | 0.192997144 | 14.53329228 | 7.45E-48 | 4.45E-47 | Upregulated |
| 322A_00265 | 81751.33331 | -3.553062483 | 0.24510338 | -14.49617908 | 1.28E-47 | 7.61E-47 | Downregulated |
| 322A_01005 | 1889.54692 | 1.715752164 | 0.11854049 | 14.47397559 | 1.77E-47 | 1.05E-46 | Upregulated |
| 322A_01225 | 2310.164533 | -1.796661326 | 0.124436859 | -14.4383372 | 2.97E-47 | 1.75E-46 | Downregulated |
| 322A_01443 | 1020.13278 | 1.601436465 | 0.111384551 | 14.37754559 | 7.16E-47 | 4.21E-46 | Upregulated |
| 322A_00483 | 3123.775337 | 1.426284698 | 0.099289248 | 14.36494607 | 8.59E-47 | 5.03E-46 | Upregulated |
| 322A_00868 | 34370.90777 | -2.616170164 | 0.182184093 | -14.3600362 | 9.22E-47 | 5.38E-46 | Downregulated |
| 322A_00689 | 12168.13118 | -2.143257263 | 0.149628013 | -14.32390376 | 1.55E-46 | 9.02E-46 | Downregulated |
| 322A_00179 | 726.5871276 | -1.613480044 | 0.112813923 | -14.30213584 | 2.12E-46 | 1.23E-45 | Downregulated |
| 322A_00794 | 1735.206211 | 1.876572503 | 0.131662106 | 14.25294311 | 4.30E-46 | 2.48E-45 | Upregulated |
| 322A_01046 | 423.4932291 | 2.367449349 | 0.166375058 | 14.22959296 | 6.00E-46 | 3.45E-45 | Upregulated |
| 322A_01092 | 7863.323508 | -1.092433101 | 0.076814476 | -14.22170865 | 6.72E-46 | 3.85E-45 | Downregulated |
| 322A_00447 | 309.3326519 | 2.71612776 | 0.191304126 | 14.19795705 | 9.43E-46 | 5.38E-45 | Upregulated |
| 322A_00392 | 2002.526278 | -1.295638726 | 0.09146321 | -14.16568175 | 1.49E-45 | 8.50E-45 | Downregulated |
| 322A_01061 | 1971.768544 | 2.456570885 | 0.173832328 | 14.1318414 | 2.42E-45 | 1.37E-44 | Upregulated |
| 322A_00990 | 431.97103 | -3.210006278 | 0.227680997 | -14.09870092 | 3.87E-45 | 2.18E-44 | Downregulated |
| 322A_00368 | 83208.85915 | -2.151418348 | 0.153033312 | -14.05849697 | 6.83E-45 | 3.84E-44 | Downregulated |
| 322A_00282 | 14088.80538 | -2.354399063 | 0.167959482 | -14.01766092 | 1.22E-44 | 6.81E-44 | Downregulated |
| 322A_00003 | 1581.860696 | 2.127674647 | 0.1522082 | 13.9787124 | 2.10E-44 | 1.17E-43 | Upregulated |
| 322A_00051 | 10643.38387 | -2.460779908 | 0.17605689 | -13.97718608 | 2.15E-44 | 1.20E-43 | Downregulated |
| 322A_01349 | 3208.894094 | 1.873960978 | 0.134282347 | 13.95537855 | 2.92E-44 | 1.62E-43 | Upregulated |
| 322A_00587 | 783.37136 | 3.817881093 | 0.273717603 | 13.94824831 | 3.22E-44 | 1.78E-43 | Upregulated |
| 322A_01533 | 2660.941776 | 1.692394116 | 0.121550446 | 13.92338881 | 4.57E-44 | 2.52E-43 | Upregulated |
| 322A_00043 | 137.6839514 | -2.938508582 | 0.211239183 | -13.9108121 | 5.45E-44 | 2.99E-43 | Downregulated |
| 322A_01493 | 300.1432759 | 3.217146039 | 0.231478569 | 13.89824575 | 6.49E-44 | 3.55E-43 | Upregulated |
| 322A_01031 | 7628.826737 | -2.339577877 | 0.168743786 | -13.86467572 | 1.04E-43 | 5.65E-43 | Downregulated |

| GeneID | Base mean | log2(FC) | StdErr | Wald-Stats | P-value | P-adj_BH | Effect of menadione on gene expression |
|---|---|---|---|---|---|---|---|
| 322A_00344 | 300.7129827 | 2.043462697 | 0.148236463 | 13.78515552 | 3.13E-43 | 1.70E-42 | Upregulated |
| 322A_00172 | 1259.395515 | -1.001234502 | 0.072644185 | -13.78272047 | 3.24E-43 | 1.75E-42 | Downregulated |
| 322A_01506 | 283.7763624 | 1.499878395 | 0.10889463 | 13.77366721 | 3.67E-43 | 1.98E-42 | Upregulated |
| 322A_00176 | 41536.93505 | 1.742939888 | 0.126633196 | 13.76368871 | 4.21E-43 | 2.26E-42 | Upregulated |
| 322A_00704 | 421.3446366 | 2.0395205 | 0.14818232 | 13.76358866 | 4.22E-43 | 2.26E-42 | Upregulated |
| 322A_01407 | 603.4372591 | 1.583027077 | 0.115155534 | 13.74686068 | 5.32E-43 | 2.84E-42 | Upregulated |
| 322A_01501 | 1241.163851 | 2.098170463 | 0.152977799 | 13.71552265 | 8.20E-43 | 4.36E-42 | Upregulated |
| 322A_01129 | 2221.312865 | -1.879158552 | 0.137277638 | -13.68874481 | 1.19E-42 | 6.29E-42 | Downregulated |
| 322A_00429 | 2731.484831 | -2.324449779 | 0.169935013 | -13.67846295 | 1.37E-42 | 7.22E-42 | Downregulated |
| 322A_00985 | 834.8541244 | -1.075555524 | 0.07882327 | -13.64515232 | 2.16E-42 | 1.14E-41 | Downregulated |
| 322A_00059 | 360.0966683 | 2.595593749 | 0.190648888 | 13.61452343 | 3.28E-42 | 1.72E-41 | Upregulated |
| 322A_00108 | 160288.6084 | -3.821462551 | 0.280754869 | -13.6113848 | 3.43E-42 | 1.79E-41 | Downregulated |
| 322A_00920 | 2632.908814 | -3.611122531 | 0.265663691 | -13.59283429 | 4.42E-42 | 2.30E-41 | Downregulated |
| 322A_00189 | 1236.899196 | -2.220252094 | 0.16377961 | -13.5563401 | 7.27E-42 | 3.78E-41 | Downregulated |
| 322A_00716 | 6332.821699 | 1.814365325 | 0.134113874 | 13.52854308 | 1.06E-41 | 5.50E-41 | Upregulated |
| 322A_00907 | 92542.56217 | -3.270695391 | 0.242652138 | -13.47894732 | 2.08E-41 | 1.07E-40 | Downregulated |
| 322A_00216 | 5999.174818 | -2.546519565 | 0.188996825 | -13.47387487 | 2.23E-41 | 1.15E-40 | Downregulated |
| 322A_00023 | 14024.12237 | -1.848139296 | 0.137209495 | -13.46947082 | 2.37E-41 | 1.21E-40 | Downregulated |
| 322A_00999 | 1278.349433 | -1.905149567 | 0.141470937 | -13.46672051 | 2.46E-41 | 1.26E-40 | Downregulated |
| 322A_00595 | 2050.944053 | -3.407369208 | 0.253105916 | -13.46222665 | 2.61E-41 | 1.33E-40 | Downregulated |
| 322A_00825 | 5561.466406 | -2.004840925 | 0.149751498 | -13.38778541 | 7.13E-41 | 3.62E-40 | Downregulated |
| 322A_01197 | 13608.73546 | -1.71995603 | 0.128556225 | -13.37901787 | 8.02E-41 | 4.06E-40 | Downregulated |
| 322A_01194 | 4687.302841 | -1.829405606 | 0.137596861 | -13.29540214 | 2.46E-40 | 1.24E-39 | Downregulated |
| 322A_00665 | 5208.987224 | 1.491594558 | 0.112386026 | 13.27206429 | 3.36E-40 | 1.69E-39 | Upregulated |
| 322A_01550 | 2260.857588 | 1.404882033 | 0.105885612 | 13.26792192 | 3.55E-40 | 1.78E-39 | Upregulated |
| 322A_00881 | 38323.07783 | -2.77831661 | 0.209466116 | -13.26379972 | 3.75E-40 | 1.88E-39 | Downregulated |
| 322A_00286 | 1477.603438 | -1.75610158 | 0.132558067 | -13.24779112 | 4.65E-40 | 2.32E-39 | Downregulated |
| 322A_00911 | 16941.62455 | -2.301856553 | 0.173806477 | -13.24379039 | 4.90E-40 | 2.44E-39 | Downregulated |
| 322A_00641 | 6001.325772 | 1.49561495 | 0.113023797 | 13.23274378 | 5.68E-40 | 2.81E-39 | Upregulated |
| 322A_00994 | 37188.52094 | -1.823209803 | 0.138028454 | -13.20894168 | 7.79E-40 | 3.85E-39 | Downregulated |
| 322A_00995 | 9528.215676 | -1.561601983 | 0.118306504 | -13.19962917 | 8.82E-40 | 4.34E-39 | Downregulated |
| 322A_01017 | 1448311.947 | -4.829930799 | 0.366963013 | -13.16190088 | 1.45E-39 | 7.14E-39 | Downregulated |
| 322A_01133 | 1038.604929 | 1.22963984 | 0.0934993 | 13.1513267 | 1.67E-39 | 8.18E-39 | Upregulated |
| 322A_00403 | 253.3784463 | 2.287697323 | 0.174824519 | 13.08567781 | 3.98E-39 | 1.94E-38 | Upregulated |
| 322A_00315 | 1521.809295 | 2.023124697 | 0.154889692 | 13.06171297 | 5.45E-39 | 2.65E-38 | Upregulated |
| 322A_00094 | 102775.6803 | -4.346795842 | 0.332910345 | -13.05695636 | 5.80E-39 | 2.81E-38 | Downregulated |
| 322A_00465 | 1117.864373 | 0.874744114 | 0.067074389 | 13.04140264 | 7.11E-39 | 3.44E-38 | Upregulated |
| 322A_00773 | 40349.82452 | -3.009712098 | 0.231285688 | -13.01296297 | 1.03E-38 | 4.98E-38 | Downregulated |
| 322A_00711 | 129515.1038 | -3.024261819 | 0.233308403 | -12.96250707 | 2.00E-38 | 9.59E-38 | Downregulated |
| 322A_00293 | 2102.478557 | 0.993960847 | 0.076688978 | 12.96093485 | 2.04E-38 | 9.76E-38 | Upregulated |
| 322A_01463 | 876.7423641 | -1.372135762 | 0.105878448 | -12.95953795 | 2.07E-38 | 9.91E-38 | Downregulated |
| 322A_00129 | 1483.086797 | 2.157928109 | 0.166600386 | 12.95271975 | 2.27E-38 | 1.08E-37 | Upregulated |

| GeneID | Base mean | log2(FC) | StdErr | Wald-Stats | P-value | P-adj_BH | Effect of menadione on gene expression |
|---|---|---|---|---|---|---|---|
| 322A_00908 | 130337.6202 | -3.460437096 | 0.267385103 | -12.9417722 | 2.62E-38 | 1.24E-37 | Downregulated |
| 322A_01586 | 610.0011817 | -1.829131379 | 0.141438354 | -12.93235765 | 2.96E-38 | 1.40E-37 | Downregulated |
| 322A_00874 | 1861.972762 | 2.008518271 | 0.155475317 | 12.91856682 | 3.54E-38 | 1.67E-37 | Upregulated |
| 322A_00797 | 2753.681043 | -0.801769793 | 0.062084205 | -12.91423154 | 3.74E-38 | 1.76E-37 | Downregulated |
| 322A_01228 | 659.7787185 | 2.027374989 | 0.157548869 | 12.86822938 | 6.79E-38 | 3.19E-37 | Upregulated |
| 322A_01417 | 723.8753776 | 1.70374503 | 0.132424344 | 12.8657993 | 7.01E-38 | 3.28E-37 | Upregulated |
| 322A_00739 | 1934.784486 | 2.612993843 | 0.20328607 | 12.85377714 | 8.19E-38 | 3.82E-37 | Upregulated |
| 322A_00887 | 2838.585168 | 1.014254507 | 0.078954445 | 12.84607221 | 9.05E-38 | 4.21E-37 | Upregulated |
| 322A_01058 | 1750.440444 | 2.001846982 | 0.155870229 | 12.84303613 | 9.41E-38 | 4.36E-37 | Upregulated |
| 322A_00844 | 13691.16837 | 1.328333672 | 0.103488453 | 12.83557376 | 1.04E-37 | 4.79E-37 | Upregulated |
| 322A_00374 | 212.6077692 | 1.844259425 | 0.143799508 | 12.82521372 | 1.18E-37 | 5.44E-37 | Upregulated |
| 322A_01368 | 1001.408034 | 2.56375995 | 0.199896974 | 12.82540652 | 1.18E-37 | 5.44E-37 | Upregulated |
| 322A_00973 | 8847.701525 | -1.248387527 | 0.097959341 | -12.74393549 | 3.37E-37 | 1.54E-36 | Downregulated |
| 322A_01538 | 2761.031567 | 1.330051801 | 0.105021363 | 12.6645833 | 9.29E-37 | 4.25E-36 | Upregulated |
| 322A_01109 | 211.1910015 | 2.591168995 | 0.204648979 | 12.66152904 | 9.66E-37 | 4.40E-36 | Upregulated |
| 322A_01514 | 41513.89713 | -2.25011674 | 0.178554202 | -12.60186946 | 2.06E-36 | 9.37E-36 | Downregulated |
| 322A_01301 | 1775.345057 | 1.410521232 | 0.112229163 | 12.56822381 | 3.16E-36 | 1.43E-35 | Upregulated |
| 322A_01203 | 8921.834999 | -2.009164924 | 0.160022135 | -12.55554379 | 3.71E-36 | 1.67E-35 | Downregulated |
| 322A_01421 | 1285.305862 | 1.629038607 | 0.129975517 | 12.53342667 | 4.90E-36 | 2.21E-35 | Upregulated |
| 322A_00128 | 655.373255 | 1.642695 | 0.131717837 | 12.4713178 | 1.07E-35 | 4.81E-35 | Upregulated |
| 322A_00263 | 64009.17736 | -2.052766165 | 0.16480387 | -12.45581286 | 1.30E-35 | 5.82E-35 | Downregulated |
| 322A_00921 | 10439.63696 | -2.625535565 | 0.210982773 | -12.44431251 | 1.50E-35 | 6.71E-35 | Downregulated |
| 322A_00460 | 2595.354169 | -3.183499508 | 0.256250524 | -12.42338729 | 1.95E-35 | 8.69E-35 | Downregulated |
| 322A_00556 | 1025.500081 | -4.218144235 | 0.339602611 | -12.4208239 | 2.01E-35 | 8.95E-35 | Downregulated |
| 322A_00134 | 51262.26152 | -2.885369003 | 0.232369668 | -12.41714994 | 2.11E-35 | 9.34E-35 | Downregulated |
| 322A_00998 | 677.7133572 | 1.979017824 | 0.159571774 | 12.40205438 | 2.55E-35 | 1.12E-34 | Upregulated |
| 322A_00158 | 35956.70453 | -1.413493883 | 0.114072278 | -12.39121295 | 2.92E-35 | 1.28E-34 | Downregulated |
| 322A_01350 | 895.8220055 | 2.372669001 | 0.191487214 | 12.39074377 | 2.93E-35 | 1.29E-34 | Upregulated |
| 322A_00572 | 4041.82714 | 1.307256859 | 0.105646708 | 12.37385329 | 3.62E-35 | 1.59E-34 | Upregulated |
| 322A_01104 | 1321.054193 | 2.469194396 | 0.20041375 | 12.32048399 | 7.03E-35 | 3.07E-34 | Upregulated |
| 322A_00541 | 3576.759047 | -0.811942476 | 0.066086621 | -12.28603406 | 1.08E-34 | 4.69E-34 | Downregulated |
| 322A_00375 | 435.8558907 | 1.906982106 | 0.1557435 | 12.24437685 | 1.80E-34 | 7.82E-34 | Upregulated |
| 322A_00400 | 3213.513087 | -3.041230774 | 0.249059254 | -12.21087243 | 2.72E-34 | 1.18E-33 | Downregulated |
| 322A_01281 | 854.8670365 | -4.344048061 | 0.3565511 | -12.1835217 | 3.80E-34 | 1.64E-33 | Downregulated |
| 322A_00882 | 29733.54556 | -2.901667136 | 0.238456848 | -12.1685209 | 4.57E-34 | 1.97E-33 | Downregulated |
| 322A_00651 | 2035.170446 | 0.917747722 | 0.075428884 | 12.16705951 | 4.66E-34 | 2.00E-33 | Upregulated |
| 322A_00979 | 1144.561998 | 1.85233736 | 0.152302725 | 12.1622076 | 4.94E-34 | 2.12E-33 | Upregulated |
| 322A_01127 | 489.5036455 | -1.933509879 | 0.159206935 | -12.14463354 | 6.13E-34 | 2.62E-33 | Downregulated |
| 322A_00742 | 538.4181243 | 1.372043205 | 0.113403786 | 12.09874247 | 1.07E-33 | 4.57E-33 | Upregulated |
| 322A_01029 | 1282.512992 | -2.419017817 | 0.200284505 | -12.07790794 | 1.38E-33 | 5.87E-33 | Downregulated |
| 322A_00173 | 1860.052822 | -2.09331312 | 0.173442412 | -12.06921132 | 1.54E-33 | 6.51E-33 | Downregulated |
| 322A_00666 | 861.0000324 | 1.728039508 | 0.143341517 | 12.05540127 | 1.82E-33 | 7.68E-33 | Upregulated |

| GeneID | Base mean | log2(FC) | StdErr | Wald-Stats | P-value | P-adj_BH | Effect of menadione on gene expression |
|---|---|---|---|---|---|---|---|
| 322A_00424 | 1295.500867 | 2.522972798 | 0.209331039 | 12.05254995 | 1.88E-33 | 7.93E-33 | Upregulated |
| 322A_00366 | 1719.320771 | -0.89016951 | 0.073953112 | -12.0369445 | 2.27E-33 | 9.55E-33 | Downregulated |
| 322A_01495 | 34245.13834 | -2.242312544 | 0.186461319 | -12.02561771 | 2.61E-33 | 1.09E-32 | Downregulated |
| 322A_00542 | 9357.212592 | -1.536242622 | 0.127904238 | -12.01088135 | 3.12E-33 | 1.30E-32 | Downregulated |
| 322A_00210 | 11117.38061 | -1.502169437 | 0.125104156 | -12.00735035 | 3.25E-33 | 1.36E-32 | Downregulated |
| 322A_00631 | 1039.532649 | 2.064417168 | 0.171964748 | 12.00488586 | 3.35E-33 | 1.39E-32 | Upregulated |
| 322A_00211 | 9278.63957 | -1.066687427 | 0.089046416 | -11.97900461 | 4.58E-33 | 1.90E-32 | Downregulated |
| 322A_00670 | 13529.10989 | -0.712313017 | 0.059492502 | -11.97315619 | 4.91E-33 | 2.03E-32 | Downregulated |
| 322A_00505 | 15636.11288 | -1.204055104 | 0.100822935 | -11.94227388 | 7.12E-33 | 2.94E-32 | Downregulated |
| 322A_01171 | 18716.74791 | -2.098653244 | 0.176036174 | -11.92171582 | 9.12E-33 | 3.76E-32 | Downregulated |
| 322A_00416 | 2271.354067 | 1.096279135 | 0.091999912 | 11.91608899 | 9.76E-33 | 4.01E-32 | Upregulated |
| 322A_00438 | 613.2282519 | 1.421292617 | 0.119286924 | 11.91490709 | 9.90E-33 | 4.05E-32 | Upregulated |
| 322A_00302 | 1115606.822 | -3.100842249 | 0.261719612 | -11.84795525 | 2.21E-32 | 9.01E-32 | Downregulated |
| 322A_00629 | 808.5188507 | 0.97117275 | 0.082002753 | 11.84317256 | 2.33E-32 | 9.51E-32 | Upregulated |
| 322A_00600 | 3867.454642 | 1.04504316 | 0.088378266 | 11.82466246 | 2.91E-32 | 1.18E-31 | Upregulated |
| 322A_01488 | 919.2928719 | 1.254676072 | 0.106325943 | 11.80028159 | 3.89E-32 | 1.58E-31 | Upregulated |
| 322A_00690 | 3940.050202 | -0.89942444 | 0.076273299 | -11.79212709 | 4.29E-32 | 1.73E-31 | Downregulated |
| 322A_01439 | 4121.901904 | 1.673264758 | 0.14200755 | 11.78292818 | 4.78E-32 | 1.93E-31 | Upregulated |
| 322A_00205 | 3158.967871 | 1.025835903 | 0.087208001 | 11.76309386 | 6.05E-32 | 2.43E-31 | Upregulated |
| 322A_01534 | 512.8293307 | 1.568984754 | 0.133480758 | 11.75438902 | 6.70E-32 | 2.69E-31 | Upregulated |
| 322A_01309 | 3626.012658 | -1.52177643 | 0.129640516 | -11.73843241 | 8.10E-32 | 3.24E-31 | Downregulated |
| 322A_00285 | 3067.310741 | -1.651792708 | 0.140866607 | -11.72593525 | 9.39E-32 | 3.75E-31 | Downregulated |
| 322A_00103 | 1826.032866 | -1.344261566 | 0.114811711 | -11.70840113 | 1.15E-31 | 4.60E-31 | Downregulated |
| 322A_01069 | 10011.50595 | -1.481184276 | 0.126569673 | -11.7025212 | 1.24E-31 | 4.91E-31 | Downregulated |
| 322A_01102 | 6808.785781 | 1.244326081 | 0.10633243 | 11.70222561 | 1.24E-31 | 4.92E-31 | Upregulated |
| 322A_01103 | 3957.383973 | 1.163285307 | 0.099444374 | 11.69784931 | 1.31E-31 | 5.17E-31 | Upregulated |
| 322A_00672 | 913.8532309 | 2.448180893 | 0.209596192 | 11.68046454 | 1.60E-31 | 6.32E-31 | Upregulated |
| 322A_00638 | 7017.997708 | 1.426914347 | 0.122657118 | 11.63335946 | 2.79E-31 | 1.10E-30 | Upregulated |
| 322A_01022 | 7107.861053 | 1.397899287 | 0.120217341 | 11.62810021 | 2.97E-31 | 1.16E-30 | Upregulated |
| 322A_00877 | 14769.88165 | -1.284680998 | 0.11059934 | -11.61562987 | 3.43E-31 | 1.34E-30 | Downregulated |
| 322A_01277 | 606.9267789 | -2.770496765 | 0.238594655 | -11.61173021 | 3.59E-31 | 1.40E-30 | Downregulated |
| 322A_01280 | 47.0567241 | -3.292741373 | 0.283934188 | -11.59684711 | 4.28E-31 | 1.66E-30 | Downregulated |
| 322A_00165 | 3982.833416 | -2.512226096 | 0.217146868 | -11.56924859 | 5.90E-31 | 2.29E-30 | Downregulated |
| 322A_01497 | 2887.731531 | -1.731165349 | 0.149734308 | -11.56158108 | 6.45E-31 | 2.50E-30 | Downregulated |
| 322A_00543 | 43626.90567 | -1.096405731 | 0.095180332 | -11.51924677 | 1.06E-30 | 4.08E-30 | Downregulated |
| 322A_01227 | 2615.521433 | 2.164450366 | 0.188702647 | 11.47016428 | 1.86E-30 | 7.18E-30 | Upregulated |
| 322A_00978 | 1285.211267 | 2.050630541 | 0.178937874 | 11.46001403 | 2.09E-30 | 8.06E-30 | Upregulated |
| 322A_01242 | 391.7567333 | 3.753530461 | 0.327788218 | 11.45108414 | 2.32E-30 | 8.91E-30 | Upregulated |
| 322A_00018 | 7343.27094 | 1.528525659 | 0.133547806 | 11.44553178 | 2.48E-30 | 9.48E-30 | Upregulated |
| 322A_00124 | 5410.133646 | 1.108351932 | 0.096862337 | 11.4425479 | 2.56E-30 | 9.79E-30 | Upregulated |
| 322A_00009 | 7149.397778 | -1.873840517 | 0.164688854 | -11.37806521 | 5.38E-30 | 2.05E-29 | Downregulated |
| 322A_00947 | 17189.24322 | 1.718842616 | 0.151212195 | 11.3670899 | 6.10E-30 | 2.32E-29 | Upregulated |

| GeneID | Base mean | log2(FC) | StdErr | Wald-Stats | P-value | P-adj_BH | Effect of menadione on gene expression |
|---|---|---|---|---|---|---|---|
| 322A_00611 | 3338.341195 | 1.323552695 | 0.11648197 | 11.36272587 | 6.41E-30 | 2.43E-29 | Upregulated |
| 322A_01502 | 1487.09346 | 1.416346531 | 0.124648773 | 11.36269934 | 6.41E-30 | 2.43E-29 | Upregulated |
| 322A_00126 | 535.9180786 | 1.367781827 | 0.120407092 | 11.35964504 | 6.64E-30 | 2.51E-29 | Upregulated |
| 322A_01371 | 1403.550945 | 1.030280026 | 0.090842587 | 11.34137698 | 8.18E-30 | 3.08E-29 | Upregulated |
| 322A_00715 | 7875.92092 | 2.09350507 | 0.184664697 | 11.33679098 | 8.62E-30 | 3.24E-29 | Upregulated |
| 322A_01279 | 4834.89661 | -1.742328132 | 0.153974147 | -11.31571871 | 1.10E-29 | 4.11E-29 | Downregulated |
| 322A_01532 | 1180.859204 | 2.739040294 | 0.242182979 | 11.30979684 | 1.17E-29 | 4.39E-29 | Upregulated |
| 322A_00661 | 2169.313273 | 1.354682423 | 0.120037318 | 11.28551062 | 1.55E-29 | 5.77E-29 | Upregulated |
| 322A_00135 | 31010.1063 | -2.126235158 | 0.188412058 | -11.28502699 | 1.56E-29 | 5.79E-29 | Downregulated |
| 322A_01345 | 968.7530696 | 1.461364688 | 0.129780597 | 11.26027098 | 2.06E-29 | 7.65E-29 | Upregulated |
| 322A_00732 | 18255.10613 | -2.006602981 | 0.178532068 | -11.23945411 | 2.61E-29 | 9.66E-29 | Downregulated |
| 322A_00869 | 12008.84023 | -2.642589064 | 0.236087087 | -11.19328085 | 4.40E-29 | 1.62E-28 | Downregulated |
| 322A_00131 | 1917.842907 | 1.203785648 | 0.108036324 | 11.14241583 | 7.80E-29 | 2.87E-28 | Upregulated |
| 322A_01174 | 1475.021115 | -1.16058448 | 0.104157964 | -11.14254189 | 7.79E-29 | 2.87E-28 | Downregulated |
| 322A_00459 | 4766.080102 | 0.866108709 | 0.077844973 | 11.12607119 | 9.37E-29 | 3.44E-28 | Upregulated |
| 322A_00042 | 78.92674293 | -3.432589778 | 0.309419809 | -11.09363292 | 1.35E-28 | 4.93E-28 | Downregulated |
| 322A_01246 | 1348.881165 | 1.936673149 | 0.174639504 | 11.08954795 | 1.41E-28 | 5.15E-28 | Upregulated |
| 322A_01522 | 1258.282229 | 0.945518768 | 0.085269969 | 11.08853182 | 1.43E-28 | 5.20E-28 | Upregulated |
| 322A_00268 | 646.1335819 | -2.463217456 | 0.222186329 | -11.0862692 | 1.46E-28 | 5.32E-28 | Downregulated |
| 322A_01381 | 5783.518197 | 2.38786443 | 0.215717817 | 11.06938899 | 1.77E-28 | 6.40E-28 | Upregulated |
| 322A_01292 | 1592.213352 | 0.744588375 | 0.067660383 | 11.00479106 | 3.62E-28 | 1.31E-27 | Upregulated |
| 322A_00445 | 4034.64837 | -1.268424449 | 0.115267513 | -11.00417985 | 3.65E-28 | 1.32E-27 | Downregulated |
| 322A_00817 | 9488.273582 | 2.143863756 | 0.195069914 | 10.99023275 | 4.26E-28 | 1.53E-27 | Upregulated |
| 322A_00428 | 614.2760024 | 1.748085812 | 0.159562925 | 10.95546355 | 6.26E-28 | 2.25E-27 | Upregulated |
| 322A_00884 | 138083.0191 | -2.700559773 | 0.246508264 | -10.95525044 | 6.27E-28 | 2.25E-27 | Downregulated |
| 322A_00040 | 137.7051607 | -2.566798197 | 0.234449174 | -10.94820747 | 6.78E-28 | 2.42E-27 | Downregulated |
| 322A_01134 | 2688.954512 | 2.184223918 | 0.199684903 | 10.93835277 | 7.56E-28 | 2.70E-27 | Upregulated |
| 322A_00095 | 789823.7633 | -3.328636359 | 0.304595775 | -10.92804509 | 8.47E-28 | 3.01E-27 | Downregulated |
| 322A_00306 | 20048.14019 | -2.128462779 | 0.194829513 | -10.92474518 | 8.78E-28 | 3.12E-27 | Downregulated |
| 322A_00242 | 2375.034912 | 1.172785565 | 0.107492618 | 10.91038241 | 1.03E-27 | 3.64E-27 | Upregulated |
| 322A_00304 | 19179.39752 | -1.686629383 | 0.155079418 | -10.87590737 | 1.50E-27 | 5.31E-27 | Downregulated |
| 322A_00581 | 744.616564 | 1.178065459 | 0.108561697 | 10.85157558 | 1.96E-27 | 6.92E-27 | Upregulated |
| 322A_00320 | 2393.748421 | -0.901023008 | 0.083076582 | -10.8456918 | 2.09E-27 | 7.36E-27 | Downregulated |
| 322A_00535 | 35875.73902 | -1.799467469 | 0.16596266 | -10.84260438 | 2.16E-27 | 7.60E-27 | Downregulated |
| 322A_00005 | 20228.47672 | -1.968792265 | 0.182158688 | -10.80811619 | 3.15E-27 | 1.10E-26 | Downregulated |
| 322A_01450 | 15308.17061 | -0.911955891 | 0.084635679 | -10.7750762 | 4.51E-27 | 1.58E-26 | Downregulated |
| 322A_01210 | 9686.392365 | -1.315879491 | 0.122508824 | -10.74109964 | 6.53E-27 | 2.28E-26 | Downregulated |
| 322A_00580 | 2397.106571 | 1.046894962 | 0.097724743 | 10.71269083 | 8.87E-27 | 3.09E-26 | Upregulated |
| 322A_01064 | 3220.2104 | -1.688543368 | 0.157898592 | -10.69384688 | 1.09E-26 | 3.78E-26 | Downregulated |
| 322A_00584 | 79.92328553 | 2.855053161 | 0.267016729 | 10.69241304 | 1.10E-26 | 3.83E-26 | Upregulated |
| 322A_00770 | 4298.749513 | -2.027750937 | 0.189948895 | -10.67524472 | 1.33E-26 | 4.60E-26 | Downregulated |
| 322A_01588 | 16180.4691 | -3.761924013 | 0.353541022 | -10.64070019 | 1.93E-26 | 6.65E-26 | Downregulated |

| GeneID | Base mean | log2(FC) | StdErr | Wald-Stats | P-value | P-adj_BH | Effect of menadione on gene expression |
|---|---|---|---|---|---|---|---|
| 322A_01128 | 284.5171989 | -1.84987628 | 0.174000622 | -10.63143485 | 2.13E-26 | 7.33E-26 | Downregulated |
| 322A_00798 | 166.7892774 | 2.101924607 | 0.197860984 | 10.62323944 | 2.32E-26 | 7.99E-26 | Upregulated |
| 322A_00245 | 532.9519865 | 1.901429814 | 0.179033759 | 10.62050992 | 2.39E-26 | 8.21E-26 | Upregulated |
| 322A_00529 | 1617.04974 | 2.053280919 | 0.193386194 | 10.61751552 | 2.47E-26 | 8.45E-26 | Upregulated |
| 322A_00008 | 3870.487976 | -2.250156703 | 0.212120528 | -10.60791582 | 2.74E-26 | 9.35E-26 | Downregulated |
| 322A_01164 | 79926.23856 | -2.568642803 | 0.242418651 | -10.59589596 | 3.11E-26 | 1.06E-25 | Downregulated |
| 322A_00766 | 37090.67313 | -2.020756902 | 0.190840176 | -10.58873948 | 3.36E-26 | 1.14E-25 | Downregulated |
| 322A_01326 | 1610.774756 | 1.639065973 | 0.155124723 | 10.566117 | 4.28E-26 | 1.45E-25 | Upregulated |
| 322A_01552 | 32726.61078 | -2.863464792 | 0.271071787 | -10.5634925 | 4.40E-26 | 1.49E-25 | Downregulated |
| 322A_00728 | 142.7644364 | 2.731337372 | 0.259429459 | 10.52824681 | 6.40E-26 | 2.16E-25 | Upregulated |
| 322A_00757 | 23747.31104 | 1.520423345 | 0.144531536 | 10.51966503 | 7.01E-26 | 2.36E-25 | Upregulated |
| 322A_00155 | 3677.300395 | 0.994628204 | 0.094735935 | 10.49895385 | 8.73E-26 | 2.94E-25 | Upregulated |
| 322A_00340 | 1546.966261 | -2.241880934 | 0.213959815 | -10.47804671 | 1.09E-25 | 3.66E-25 | Downregulated |
| 322A_00969 | 1622.168504 | -1.826630615 | 0.174555853 | -10.46444781 | 1.26E-25 | 4.21E-25 | Downregulated |
| 322A_00377 | 223.6280121 | 1.538617151 | 0.147365635 | 10.44081378 | 1.61E-25 | 5.40E-25 | Upregulated |
| 322A_00521 | 1936.381072 | 1.873184391 | 0.180094676 | 10.4011092 | 2.45E-25 | 8.17E-25 | Upregulated |
| 322A_00102 | 3530.305513 | -1.172067263 | 0.112700083 | -10.39987929 | 2.48E-25 | 8.26E-25 | Downregulated |
| 322A_00880 | 2816.31441 | -1.575101175 | 0.151993061 | -10.36298082 | 3.65E-25 | 1.21E-24 | Downregulated |
| 322A_00062 | 884.4507564 | 1.405009124 | 0.135587838 | 10.36235361 | 3.68E-25 | 1.22E-24 | Upregulated |
| 322A_01559 | 166.2130463 | -2.641557514 | 0.255140878 | -10.35332923 | 4.04E-25 | 1.34E-24 | Downregulated |
| 322A_01049 | 3735.20334 | -1.413242679 | 0.13652919 | -10.35121262 | 4.13E-25 | 1.36E-24 | Downregulated |
| 322A_01192 | 132315.0631 | -2.342303483 | 0.2266322 | -10.33526341 | 4.88E-25 | 1.61E-24 | Downregulated |
| 322A_00253 | 13099.50631 | -1.719937873 | 0.166840903 | -10.3088502 | 6.43E-25 | 2.11E-24 | Downregulated |
| 322A_00682 | 545.2791714 | 1.596076258 | 0.154935149 | 10.3015763 | 6.93E-25 | 2.27E-24 | Upregulated |
| 322A_00705 | 2361.019288 | 0.881837184 | 0.085724347 | 10.28689299 | 8.07E-25 | 2.64E-24 | Upregulated |
| 322A_01509 | 18580.48624 | 0.563467263 | 0.055028793 | 10.23949876 | 1.32E-24 | 4.31E-24 | Upregulated |
| 322A_00769 | 6394.344175 | -1.896998214 | 0.185351595 | -10.23459343 | 1.39E-24 | 4.52E-24 | Downregulated |
| 322A_00001 | 31278.55666 | 1.61424326 | 0.158002831 | 10.21654645 | 1.67E-24 | 5.44E-24 | Upregulated |
| 322A_00534 | 179469.2872 | -2.355358567 | 0.231464023 | -10.1759165 | 2.54E-24 | 8.25E-24 | Downregulated |
| 322A_01594 | 5341.101114 | -2.443193401 | 0.241874647 | -10.10107272 | 5.46E-24 | 1.77E-23 | Downregulated |
| 322A_01213 | 9015.545784 | -1.610046723 | 0.159529822 | -10.0924498 | 5.97E-24 | 1.93E-23 | Downregulated |
| 322A_00082 | 9928.38935 | -2.321628399 | 0.230183294 | -10.08599866 | 6.37E-24 | 2.06E-23 | Downregulated |
| 322A_00052 | 52742.4669 | -1.462727323 | 0.145051957 | -10.08416127 | 6.49E-24 | 2.09E-23 | Downregulated |
| 322A_00767 | 14255.06144 | -2.05213741 | 0.203579356 | -10.08028247 | 6.75E-24 | 2.17E-23 | Downregulated |
| 322A_01537 | 29600.09377 | -1.680342296 | 0.167038274 | -10.05962438 | 8.33E-24 | 2.67E-23 | Downregulated |
| 322A_01376 | 1091.094056 | 3.219268299 | 0.320426726 | 10.04681582 | 9.49E-24 | 3.04E-23 | Upregulated |
| 322A_00539 | 19696.29244 | -1.581315697 | 0.157773447 | -10.02269857 | 1.21E-23 | 3.87E-23 | Downregulated |
| 322A_00700 | 10710.08024 | -1.339703911 | 0.133909595 | -10.00454007 | 1.46E-23 | 4.64E-23 | Downregulated |
| 322A_00278 | 2067.812215 | 1.387631892 | 0.138719244 | 10.0031679 | 1.48E-23 | 4.70E-23 | Upregulated |
| 322A_00754 | 1826.708852 | 1.21836642 | 0.121859931 | 9.998088848 | 1.55E-23 | 4.93E-23 | Upregulated |
| 322A_00912 | 9844.845428 | -2.846389485 | 0.284774725 | -9.995232144 | 1.60E-23 | 5.07E-23 | Downregulated |
| 322A_01236 | 11775.59897 | -2.160125471 | 0.216421634 | -9.981097685 | 1.84E-23 | 5.83E-23 | Downregulated |

| GeneID | Base mean | log2(FC) | StdErr | Wald-Stats | P-value | P-adj_BH | Effect of menadione on gene expression |
|---|---|---|---|---|---|---|---|
| 322A_00867 | 2552.710303 | -2.433456694 | 0.244038733 | -9.971600241 | 2.03E-23 | 6.40E-23 | Downregulated |
| 322A_00456 | 3008.28297 | 1.963835096 | 0.196979055 | 9.969766041 | 2.07E-23 | 6.51E-23 | Upregulated |
| 322A_01344 | 563.7642528 | 1.593160629 | 0.159922737 | 9.962064558 | 2.23E-23 | 7.02E-23 | Upregulated |
| 322A_01115 | 206.1663122 | -2.240013717 | 0.225040373 | -9.95383044 | 2.43E-23 | 7.61E-23 | Downregulated |
| 322A_01021 | 54449.39831 | 1.632985731 | 0.164391518 | 9.93351573 | 2.98E-23 | 9.32E-23 | Upregulated |
| 322A_00895 | 107.7067069 | 3.752083247 | 0.378142733 | 9.922399461 | 3.33E-23 | 1.04E-22 | Upregulated |
| 322A_00619 | 10705.53042 | -0.988909046 | 0.099751517 | -9.913724371 | 3.63E-23 | 1.13E-22 | Downregulated |
| 322A_01232 | 9478.275966 | -2.104794877 | 0.21250313 | -9.904771162 | 3.97E-23 | 1.24E-22 | Downregulated |
| 322A_01010 | 1830.514025 | 0.772841155 | 0.078087264 | 9.897147338 | 4.28E-23 | 1.33E-22 | Upregulated |
| 322A_01053 | 1094.558514 | -1.126262934 | 0.113805717 | -9.896365181 | 4.32E-23 | 1.34E-22 | Downregulated |
| 322A_00566 | 6473.658676 | -1.977459498 | 0.200097878 | -9.882461109 | 4.96E-23 | 1.53E-22 | Downregulated |
| 322A_00684 | 182.8488713 | 2.24613584 | 0.227379286 | 9.878366161 | 5.17E-23 | 1.59E-22 | Upregulated |
| 322A_01078 | 1053.738579 | 0.733241702 | 0.074225987 | 9.87850393 | 5.16E-23 | 1.59E-22 | Upregulated |
| 322A_01145 | 2877.515987 | -1.574745605 | 0.159438346 | -9.876831064 | 5.25E-23 | 1.61E-22 | Downregulated |
| 322A_00426 | 1253.675878 | 0.65117646 | 0.066016805 | 9.863798535 | 5.97E-23 | 1.83E-22 | Upregulated |
| 322A_00509 | 2326.22318 | -1.05890825 | 0.107367953 | -9.862423747 | 6.06E-23 | 1.86E-22 | Downregulated |
| 322A_01602 | 29682.96712 | -2.182533046 | 0.221439416 | -9.856118148 | 6.45E-23 | 1.97E-22 | Downregulated |
| 322A_01604 | 7260.405495 | -2.596945669 | 0.263855184 | -9.842314357 | 7.40E-23 | 2.26E-22 | Downregulated |
| 322A_01284 | 407.8653957 | -1.379916314 | 0.140506048 | -9.821045681 | 9.14E-23 | 2.78E-22 | Downregulated |
| 322A_00283 | 528.2069659 | -2.306707992 | 0.23501884 | -9.814991832 | 9.70E-23 | 2.95E-22 | Downregulated |
| 322A_01598 | 1285.452086 | -1.222272336 | 0.124599055 | -9.809643714 | 1.02E-22 | 3.11E-22 | Downregulated |
| 322A_00916 | 9989.514305 | -1.183450057 | 0.121091262 | -9.773207729 | 1.47E-22 | 4.44E-22 | Downregulated |
| 322A_01365 | 5238.858947 | -1.174844501 | 0.120366457 | -9.760563945 | 1.66E-22 | 5.02E-22 | Downregulated |
| 322A_00792 | 2265.469364 | 2.087602559 | 0.214003862 | 9.754976108 | 1.76E-22 | 5.30E-22 | Upregulated |
| 322A_00345 | 12987.75271 | -1.118523748 | 0.115292667 | -9.701603522 | 2.97E-22 | 8.94E-22 | Downregulated |
| 322A_01426 | 3763.29277 | -0.794012608 | 0.082083683 | -9.673208854 | 3.92E-22 | 1.18E-21 | Downregulated |
| 322A_00727 | 1620.585337 | 1.821209663 | 0.188460389 | 9.66362043 | 4.30E-22 | 1.29E-21 | Upregulated |
| 322A_00692 | 4748.707918 | -1.294421291 | 0.134203285 | -9.645228095 | 5.15E-22 | 1.54E-21 | Downregulated |
| 322A_00532 | 3173.51426 | -0.786990845 | 0.081602952 | -9.644146702 | 5.20E-22 | 1.56E-21 | Downregulated |
| 322A_00206 | 87138.91311 | -1.107319284 | 0.114856523 | -9.640891582 | 5.37E-22 | 1.60E-21 | Downregulated |
| 322A_01445 | 5622.252243 | -1.406114649 | 0.145882946 | -9.638649919 | 5.49E-22 | 1.63E-21 | Downregulated |
| 322A_01146 | 6111.910642 | -1.013771014 | 0.105810635 | -9.58099356 | 9.61E-22 | 2.86E-21 | Downregulated |
| 322A_00011 | 869.4659426 | 0.864837292 | 0.09031348 | 9.575949133 | 1.01E-21 | 2.99E-21 | Upregulated |
| 322A_00060 | 5792.08122 | -0.559801593 | 0.058513496 | -9.567050943 | 1.10E-21 | 3.26E-21 | Downregulated |
| 322A_00227 | 3144.722366 | -0.756688057 | 0.079191061 | -9.555220614 | 1.23E-21 | 3.64E-21 | Downregulated |
| 322A_01166 | 2053.205271 | 2.284631363 | 0.239124514 | 9.554149517 | 1.25E-21 | 3.68E-21 | Upregulated |
| 322A_00993 | 14593.56434 | -2.13105534 | 0.223380537 | -9.54002244 | 1.43E-21 | 4.20E-21 | Downregulated |
| 322A_00590 | 10885.4156 | 1.044022967 | 0.109722433 | 9.51512772 | 1.81E-21 | 5.33E-21 | Upregulated |
| 322A_00622 | 251.4114258 | 1.864969076 | 0.196640102 | 9.48417466 | 2.44E-21 | 7.17E-21 | Upregulated |
| 322A_00913 | 14763.21551 | -2.527771654 | 0.267021417 | -9.466550234 | 2.89E-21 | 8.47E-21 | Downregulated |
| 322A_00829 | 115.2314612 | 1.729346866 | 0.182749978 | 9.462911507 | 2.99E-21 | 8.75E-21 | Upregulated |
| 322A_00267 | 55939.86987 | -1.883256042 | 0.199133368 | -9.457260029 | 3.16E-21 | 9.22E-21 | Downregulated |

| GeneID | Base mean | log2(FC) | StdErr | Wald-Stats | P-value | P-adj_BH | Effect of menadione on gene expression |
|---|---|---|---|---|---|---|---|
| 322A_01148 | 6075.422066 | -0.610238949 | 0.064655572 | -9.438304046 | 3.79E-21 | 1.10E-20 | Downregulated |
| 322A_00453 | 39072.49066 | -1.804690509 | 0.191421391 | -9.42784136 | 4.19E-21 | 1.22E-20 | Downregulated |
| 322A_00044 | 87.19930729 | -3.153097685 | 0.334581324 | -9.424009818 | 4.34E-21 | 1.26E-20 | Downregulated |
| 322A_00143 | 5621.492797 | 0.720786785 | 0.076696309 | 9.397933176 | 5.56E-21 | 1.61E-20 | Upregulated |
| 322A_00965 | 897.3587964 | 2.553728259 | 0.272705844 | 9.364406048 | 7.65E-21 | 2.21E-20 | Upregulated |
| 322A_00608 | 63332.82634 | -2.351296453 | 0.251111616 | -9.363551131 | 7.71E-21 | 2.22E-20 | Downregulated |
| 322A_00006 | 9310.654046 | -1.087196098 | 0.116137912 | -9.36125058 | 7.88E-21 | 2.27E-20 | Downregulated |
| 322A_00339 | 333.860793 | -2.78525641 | 0.298009181 | -9.346210077 | 9.08E-21 | 2.61E-20 | Downregulated |
| 322A_00986 | 3870.764478 | -0.869400346 | 0.093185049 | -9.329826538 | 1.06E-20 | 3.04E-20 | Downregulated |
| 322A_01592 | 9651.844502 | -2.04421366 | 0.219506969 | -9.312750595 | 1.25E-20 | 3.57E-20 | Downregulated |
| 322A_00123 | 3367.20881 | -0.75111273 | 0.080944304 | -9.279377179 | 1.70E-20 | 4.87E-20 | Downregulated |
| 322A_00232 | 2866.58519 | 0.779038916 | 0.084212776 | 9.250839949 | 2.23E-20 | 6.36E-20 | Upregulated |
| 322A_00816 | 7614.428829 | 1.797382531 | 0.194643952 | 9.234206948 | 2.60E-20 | 7.41E-20 | Upregulated |
| 322A_01448 | 4879.700265 | -1.198414598 | 0.129822353 | -9.231188406 | 2.68E-20 | 7.61E-20 | Downregulated |
| 322A_00351 | 42418.51553 | -2.437346681 | 0.265029816 | -9.196499908 | 3.70E-20 | 1.05E-19 | Downregulated |
| 322A_01123 | 617.3174171 | -2.171641824 | 0.236339005 | -9.188672945 | 3.98E-20 | 1.13E-19 | Downregulated |
| 322A_00612 | 1460.500569 | 0.911839849 | 0.099289847 | 9.183616246 | 4.17E-20 | 1.18E-19 | Upregulated |
| 322A_00866 | 3634.229293 | -1.588797506 | 0.173300833 | -9.167858467 | 4.83E-20 | 1.36E-19 | Downregulated |
| 322A_00485 | 1464.690968 | -0.960651709 | 0.105057258 | -9.144077516 | 6.01E-20 | 1.69E-19 | Downregulated |
| 322A_01289 | 2056.061041 | 1.460670512 | 0.159817286 | 9.1396278 | 6.27E-20 | 1.76E-19 | Upregulated |
| 322A_00910 | 3573.803502 | -1.937523344 | 0.212132501 | -9.133552552 | 6.63E-20 | 1.86E-19 | Downregulated |
| 322A_01382 | 25406.56258 | 1.672820924 | 0.183392634 | 9.121527357 | 7.41E-20 | 2.08E-19 | Upregulated |
| 322A_00080 | 97555.00923 | -2.189035708 | 0.240717059 | -9.0938121 | 9.56E-20 | 2.68E-19 | Downregulated |
| 322A_00099 | 822.6158727 | -2.936910507 | 0.323297518 | -9.084234634 | 1.04E-19 | 2.92E-19 | Downregulated |
| 322A_00745 | 2191.735075 | -2.846620642 | 0.313917161 | -9.068063162 | 1.21E-19 | 3.38E-19 | Downregulated |
| 322A_00114 | 14306.29975 | -1.762148202 | 0.194860797 | -9.043112978 | 1.52E-19 | 4.24E-19 | Downregulated |
| 322A_01391 | 1346.504069 | -1.474610437 | 0.163105518 | -9.040837205 | 1.55E-19 | 4.32E-19 | Downregulated |
| 322A_01613 | 1194.338389 | 0.83771884 | 0.092693216 | 9.037542032 | 1.60E-19 | 4.44E-19 | Upregulated |
| 322A_00662 | 215.1662311 | 2.258536454 | 0.250054561 | 9.032174615 | 1.68E-19 | 4.66E-19 | Upregulated |
| 322A_01052 | 498.1263844 | 1.109620906 | 0.122893265 | 9.029143329 | 1.73E-19 | 4.78E-19 | Upregulated |
| 322A_00702 | 1725.434972 | 1.008735017 | 0.1118354 | 9.019818542 | 1.88E-19 | 5.20E-19 | Upregulated |
| 322A_00175 | 4041.970672 | -1.010254924 | 0.112064316 | -9.014956389 | 1.97E-19 | 5.42E-19 | Downregulated |
| 322A_00585 | 3114.236472 | 1.221341761 | 0.135595767 | 9.007226287 | 2.11E-19 | 5.81E-19 | Upregulated |
| 322A_01543 | 410.2427154 | 0.930572587 | 0.103434847 | 8.996702904 | 2.33E-19 | 6.38E-19 | Upregulated |
| 322A_00363 | 813.8747148 | -1.079198168 | 0.120126386 | -8.983856121 | 2.61E-19 | 7.16E-19 | Downregulated |
| 322A_00266 | 15265.49294 | -2.968878986 | 0.330646192 | -8.979020654 | 2.73E-19 | 7.47E-19 | Downregulated |
| 322A_00768 | 9890.483408 | -1.657963466 | 0.184932614 | -8.965230245 | 3.10E-19 | 8.45E-19 | Downregulated |
| 322A_01322 | 13012.80137 | -2.375950141 | 0.265662862 | -8.943478691 | 3.77E-19 | 1.03E-18 | Downregulated |
| 322A_01007 | 4915.811177 | 1.700433148 | 0.190417017 | 8.930048221 | 4.26E-19 | 1.16E-18 | Upregulated |
| 322A_01070 | 13316.08408 | -2.062935216 | 0.231492438 | -8.911458322 | 5.04E-19 | 1.37E-18 | Downregulated |
| 322A_00168 | 3604.049949 | 0.706542016 | 0.079407911 | 8.897627515 | 5.71E-19 | 1.55E-18 | Upregulated |
| 322A_00649 | 524.4528675 | -1.367734102 | 0.153937778 | -8.884980161 | 6.39E-19 | 1.73E-18 | Downregulated |

| GeneID | Base mean | log2(FC) | StdErr | Wald-Stats | P-value | P-adj_BH | Effect of menadione on gene expression |
|---|---|---|---|---|---|---|---|
| 322A_00842 | 1101.809638 | 1.358538859 | 0.153790579 | 8.833693639 | 1.01E-18 | 2.74E-18 | Upregulated |
| 322A_00303 | 160226.0877 | -2.245612222 | 0.254381048 | -8.827749714 | 1.07E-18 | 2.88E-18 | Downregulated |
| 322A_00645 | 1583.003575 | 2.181569582 | 0.248470505 | 8.779994154 | 1.63E-18 | 4.40E-18 | Upregulated |
| 322A_00246 | 373.8753722 | 1.436188269 | 0.163751828 | 8.770517497 | 1.78E-18 | 4.78E-18 | Upregulated |
| 322A_00034 | 22591.80629 | -1.380998197 | 0.157673626 | -8.758587154 | 1.98E-18 | 5.31E-18 | Downregulated |
| 322A_01190 | 1713.157508 | -1.949880013 | 0.223258124 | -8.733747179 | 2.46E-18 | 6.60E-18 | Downregulated |
| 322A_01465 | 963.9006124 | -1.223741015 | 0.140118128 | -8.733638033 | 2.47E-18 | 6.60E-18 | Downregulated |
| 322A_00249 | 876.0824976 | 0.870356302 | 0.099692051 | 8.730448313 | 2.54E-18 | 6.77E-18 | Upregulated |
| 322A_01393 | 1750.544588 | 2.097035896 | 0.240406625 | 8.722870673 | 2.71E-18 | 7.23E-18 | Upregulated |
| 322A_00373 | 923.8445382 | 1.193589398 | 0.136982701 | 8.713431648 | 2.95E-18 | 7.85E-18 | Upregulated |
| 322A_00434 | 629.0966951 | 0.916481891 | 0.105185537 | 8.713002943 | 2.96E-18 | 7.86E-18 | Upregulated |
| 322A_00923 | 4571.784238 | -0.997316459 | 0.114530506 | -8.707867395 | 3.10E-18 | 8.21E-18 | Downregulated |
| 322A_01083 | 2434.833622 | -1.976705485 | 0.227049784 | -8.706044336 | 3.15E-18 | 8.33E-18 | Downregulated |
| 322A_01314 | 18219.18062 | -1.03525222 | 0.118957556 | -8.702702476 | 3.24E-18 | 8.57E-18 | Downregulated |
| 322A_00081 | 89892.85909 | -1.883143235 | 0.216800578 | -8.686061894 | 3.75E-18 | 9.90E-18 | Downregulated |
| 322A_00139 | 8969.742941 | -1.41536822 | 0.163060423 | -8.680023014 | 3.96E-18 | 1.04E-17 | Downregulated |
| 322A_01420 | 551.9976825 | 1.4658391 | 0.169259616 | 8.660300256 | 4.71E-18 | 1.24E-17 | Upregulated |
| 322A_01185 | 10383.159 | 1.439595014 | 0.166597232 | 8.641170056 | 5.56E-18 | 1.46E-17 | Upregulated |
| 322A_01587 | 561.2332217 | -1.450897568 | 0.167959997 | -8.638351932 | 5.70E-18 | 1.50E-17 | Downregulated |
| 322A_00085 | 5899.929504 | -0.851949801 | 0.098769123 | -8.625669414 | 6.37E-18 | 1.67E-17 | Downregulated |
| 322A_00958 | 5168.318588 | 1.099654829 | 0.127592551 | 8.618487648 | 6.78E-18 | 1.77E-17 | Upregulated |
| 322A_01268 | 364.5669949 | -2.218322456 | 0.257480605 | -8.615493406 | 6.96E-18 | 1.82E-17 | Downregulated |
| 322A_01615 | 3855.670609 | -2.581207593 | 0.300153198 | -8.599633834 | 8.00E-18 | 2.08E-17 | Downregulated |
| 322A_01012 | 876.1939558 | 0.91503595 | 0.106507479 | 8.591283521 | 8.60E-18 | 2.24E-17 | Upregulated |
| 322A_01438 | 2020.450761 | 1.164891872 | 0.136215681 | 8.551819162 | 1.21E-17 | 3.15E-17 | Upregulated |
| 322A_01188 | 497.8966832 | 2.48312909 | 0.290895187 | 8.536164239 | 1.39E-17 | 3.60E-17 | Upregulated |
| 322A_00643 | 1291.600085 | -1.21779683 | 0.142749426 | -8.531010327 | 1.45E-17 | 3.75E-17 | Downregulated |
| 322A_01553 | 87333.99205 | -2.692859362 | 0.315760174 | -8.528179245 | 1.49E-17 | 3.84E-17 | Downregulated |
| 322A_01099 | 12287.66629 | 0.902269452 | 0.106178087 | 8.497699263 | 1.93E-17 | 4.99E-17 | Upregulated |
| 322A_00747 | 1417.098273 | 1.400508416 | 0.164835453 | 8.496402887 | 1.96E-17 | 5.04E-17 | Upregulated |
| 322A_00256 | 1748.33731 | 1.194786652 | 0.140700446 | 8.491704786 | 2.04E-17 | 5.23E-17 | Upregulated |
| 322A_00899 | 1036.175102 | 1.045812961 | 0.123180924 | 8.490056133 | 2.07E-17 | 5.30E-17 | Upregulated |
| 322A_01454 | 4006.048285 | -0.871007492 | 0.102705419 | -8.480638086 | 2.24E-17 | 5.74E-17 | Downregulated |
| 322A_00038 | 3074.532013 | -0.458408292 | 0.054227913 | -8.453364065 | 2.83E-17 | 7.24E-17 | Downregulated |
| 322A_00885 | 73409.09498 | -2.242470475 | 0.265331881 | -8.45156816 | 2.87E-17 | 7.34E-17 | Downregulated |
| 322A_00857 | 2280.906998 | 1.257569851 | 0.149031524 | 8.43828082 | 3.22E-17 | 8.21E-17 | Upregulated |
| 322A_01351 | 12902.51028 | 1.024710088 | 0.121500159 | 8.433816826 | 3.35E-17 | 8.52E-17 | Upregulated |
| 322A_00313 | 1985.670174 | 1.842117129 | 0.218884847 | 8.415918927 | 3.90E-17 | 9.91E-17 | Upregulated |
| 322A_00736 | 1408.113821 | -1.559185221 | 0.185283854 | -8.415116504 | 3.93E-17 | 9.96E-17 | Downregulated |
| 322A_01147 | 474.7221165 | 1.312454288 | 0.156220213 | 8.401309038 | 4.42E-17 | 1.12E-16 | Upregulated |
| 322A_00144 | 3385.385629 | 0.714615382 | 0.085315098 | 8.376188973 | 5.47E-17 | 1.38E-16 | Upregulated |
| 322A_00879 | 3818.925338 | -0.644737991 | 0.077038569 | -8.369028649 | 5.81E-17 | 1.47E-16 | Downregulated |

| GeneID | Base mean | log2(FC) | StdErr | Wald-Stats | P-value | P-adj_BH | Effect of menadione on gene expression |
|---|---|---|---|---|---|---|---|
| 322A_00321 | 1654.952481 | 1.240784784 | 0.148568204 | 8.351617326 | 6.73E-17 | 1.70E-16 | Upregulated |
| 322A_00389 | 1911.282994 | -1.272574646 | 0.152468606 | -8.346470016 | 7.03E-17 | 1.77E-16 | Downregulated |
| 322A_01505 | 888.1240514 | 1.783902854 | 0.213950848 | 8.337909727 | 7.56E-17 | 1.90E-16 | Upregulated |
| 322A_01285 | 638.6919896 | -2.296290707 | 0.275578575 | -8.332616954 | 7.91E-17 | 1.98E-16 | Downregulated |
| 322A_00378 | 18453.88239 | -1.389645314 | 0.167053805 | -8.318549313 | 8.90E-17 | 2.23E-16 | Downregulated |
| 322A_00194 | 7862.802647 | -1.180550481 | 0.141932191 | -8.317707695 | 8.97E-17 | 2.24E-16 | Downregulated |
| 322A_00372 | 1316.015623 | 0.773864959 | 0.093327996 | 8.291884423 | 1.11E-16 | 2.78E-16 | Upregulated |
| 322A_01282 | 154.8983202 | -1.245767569 | 0.150269713 | -8.290210633 | 1.13E-16 | 2.82E-16 | Downregulated |
| 322A_00508 | 21471.64708 | -0.800725191 | 0.096623301 | -8.287081699 | 1.16E-16 | 2.89E-16 | Downregulated |
| 322A_00475 | 541.7847888 | -1.373205243 | 0.166124537 | -8.266119313 | 1.38E-16 | 3.44E-16 | Downregulated |
| 322A_01331 | 9766.965251 | -0.816454818 | 0.099011095 | -8.246094232 | 1.64E-16 | 4.06E-16 | Downregulated |
| 322A_01513 | 452.1097286 | 1.61398286 | 0.19579849 | 8.243081231 | 1.68E-16 | 4.16E-16 | Upregulated |
| 322A_00439 | 3832.179379 | 0.810217269 | 0.0984433 | 8.230293629 | 1.87E-16 | 4.62E-16 | Upregulated |
| 322A_01079 | 652.6332398 | -3.146360623 | 0.382903776 | -8.217105238 | 2.08E-16 | 5.15E-16 | Downregulated |
| 322A_00015 | 40618.72377 | -2.196998764 | 0.268218534 | -8.191077358 | 2.59E-16 | 6.39E-16 | Downregulated |
| 322A_01591 | 855.601653 | -1.910106323 | 0.233310148 | -8.186983447 | 2.68E-16 | 6.60E-16 | Downregulated |
| 322A_00750 | 2932.335213 | 0.468888836 | 0.057281558 | 8.185685788 | 2.71E-16 | 6.65E-16 | Upregulated |
| 322A_01199 | 5424.582297 | -1.075910554 | 0.13143508 | -8.18587057 | 2.70E-16 | 6.65E-16 | Downregulated |
| 322A_00096 | 2249.99647 | 0.959620645 | 0.117528185 | 8.165025676 | 3.21E-16 | 7.88E-16 | Upregulated |
| 322A_00398 | 842.7330459 | -0.718787611 | 0.088196042 | -8.149885094 | 3.64E-16 | 8.92E-16 | Downregulated |
| 322A_00180 | 1571.176674 | -1.096208824 | 0.134660741 | -8.140522735 | 3.94E-16 | 9.62E-16 | Downregulated |
| 322A_01400 | 437.6513006 | -2.010016581 | 0.247035199 | -8.13655944 | 4.07E-16 | 9.92E-16 | Downregulated |
| 322A_00731 | 167.4652237 | 1.741528335 | 0.214087885 | 8.134642156 | 4.13E-16 | 1.01E-15 | Upregulated |
| 322A_00163 | 2659.768629 | 1.049852519 | 0.129431938 | 8.111232295 | 5.01E-16 | 1.22E-15 | Upregulated |
| 322A_01138 | 560.6037269 | -1.563418947 | 0.192883516 | -8.10550832 | 5.25E-16 | 1.28E-15 | Downregulated |
| 322A_01385 | 2089.590173 | 1.120983914 | 0.138425068 | 8.098127959 | 5.58E-16 | 1.35E-15 | Upregulated |
| 322A_01105 | 899.9395853 | 1.736810066 | 0.214956353 | 8.079826635 | 6.49E-16 | 1.57E-15 | Upregulated |
| 322A_00655 | 94.67752056 | 2.130979541 | 0.264026647 | 8.071077528 | 6.97E-16 | 1.68E-15 | Upregulated |
| 322A_00215 | 3789.768609 | -1.056302403 | 0.130925443 | -8.067968918 | 7.15E-16 | 1.73E-15 | Downregulated |
| 322A_00676 | 221.3258306 | 1.414683421 | 0.175594849 | 8.056520045 | 7.85E-16 | 1.89E-15 | Upregulated |
| 322A_01536 | 8363.801713 | -1.403345943 | 0.174798036 | -8.028385083 | 9.88E-16 | 2.38E-15 | Downregulated |
| 322A_00952 | 333.0977215 | -1.515289436 | 0.188892836 | -8.021952909 | 1.04E-15 | 2.50E-15 | Downregulated |
| 322A_01457 | 9159.876716 | -1.69398784 | 0.21183121 | -7.996875641 | 1.28E-15 | 3.06E-15 | Downregulated |
| 322A_00259 | 1256.382254 | 1.616428174 | 0.202230927 | 7.992982063 | 1.32E-15 | 3.16E-15 | Upregulated |
| 322A_00695 | 1648.659666 | -1.798540855 | 0.225033845 | -7.992312704 | 1.32E-15 | 3.16E-15 | Downregulated |
| 322A_01558 | 7017.417811 | -1.061091348 | 0.132762591 | -7.99239709 | 1.32E-15 | 3.16E-15 | Downregulated |
| 322A_00886 | 1159.205327 | -0.916067182 | 0.114622757 | -7.992018402 | 1.33E-15 | 3.17E-15 | Downregulated |
| 322A_01597 | 10819.45065 | -0.871023976 | 0.109118989 | -7.982331786 | 1.44E-15 | 3.42E-15 | Downregulated |
| 322A_01217 | 25682.83484 | -1.940042176 | 0.243068897 | -7.981449718 | 1.45E-15 | 3.44E-15 | Downregulated |
| 322A_00091 | 13971.0046 | -1.997199637 | 0.251058642 | -7.95511209 | 1.79E-15 | 4.25E-15 | Downregulated |
| 322A_01255 | 2915.706613 | 1.701766282 | 0.214100596 | 7.948442532 | 1.89E-15 | 4.48E-15 | Upregulated |
| 322A_01459 | 191.4920216 | -2.029962301 | 0.255395846 | -7.948298034 | 1.89E-15 | 4.48E-15 | Downregulated |

| GeneID | Base mean | log2(FC) | StdErr | Wald-Stats | P-value | P-adj_BH | Effect of menadione on gene expression |
|---|---|---|---|---|---|---|---|
| 322A_00401 | 1361.259475 | 1.818231768 | 0.229029408 | 7.938857226 | 2.04E-15 | 4.82E-15 | Upregulated |
| 322A_00269 | 29676.03174 | -1.902055808 | 0.239618816 | -7.937839932 | 2.06E-15 | 4.85E-15 | Downregulated |
| 322A_00555 | 1382294.905 | -1.985070613 | 0.250158309 | -7.935257554 | 2.10E-15 | 4.95E-15 | Downregulated |
| 322A_01042 | 13631.24368 | -1.195568403 | 0.15087136 | -7.924422519 | 2.29E-15 | 5.39E-15 | Downregulated |
| 322A_01233 | 5218.070902 | -1.472522515 | 0.18596683 | -7.918199792 | 2.41E-15 | 5.66E-15 | Downregulated |
| 322A_00121 | 208.1288653 | 1.552102765 | 0.196535645 | 7.89730922 | 2.85E-15 | 6.68E-15 | Upregulated |
| 322A_00961 | 6128.302119 | 0.711868047 | 0.09033744 | 7.880099804 | 3.27E-15 | 7.66E-15 | Upregulated |
| 322A_00919 | 2030.508389 | -0.582849475 | 0.074217457 | -7.853266563 | 4.05E-15 | 9.48E-15 | Downregulated |
| 322A_00254 | 3380.385132 | -1.701477958 | 0.217396745 | -7.826602717 | 5.01E-15 | 1.17E-14 | Downregulated |
| 322A_01549 | 2453.373096 | -1.738513831 | 0.222490676 | -7.813872755 | 5.55E-15 | 1.29E-14 | Downregulated |
| 322A_01224 | 866.5028483 | -1.313909765 | 0.16822091 | -7.810620942 | 5.69E-15 | 1.33E-14 | Downregulated |
| 322A_00260 | 3764.961897 | 1.055523359 | 0.135275195 | 7.802785718 | 6.06E-15 | 1.41E-14 | Upregulated |
| 322A_01435 | 71.13120815 | 2.486256956 | 0.318724143 | 7.800654603 | 6.16E-15 | 1.43E-14 | Upregulated |
| 322A_00537 | 838.9092111 | -0.813726525 | 0.104515767 | -7.785682024 | 6.93E-15 | 1.61E-14 | Downregulated |
| 322A_01112 | 93.16841624 | -1.720844853 | 0.221288938 | -7.776461249 | 7.46E-15 | 1.73E-14 | Downregulated |
| 322A_01573 | 15602.9929 | -1.45650416 | 0.187342156 | -7.77456708 | 7.57E-15 | 1.75E-14 | Downregulated |
| 322A_01378 | 4478.171233 | 1.276930699 | 0.164323852 | 7.770817709 | 7.80E-15 | 1.80E-14 | Upregulated |
| 322A_00621 | 60542.25825 | -1.140578699 | 0.146996792 | -7.759208117 | 8.55E-15 | 1.97E-14 | Downregulated |
| 322A_00369 | 3082.765786 | -1.074813288 | 0.138854076 | -7.740595864 | 9.90E-15 | 2.28E-14 | Downregulated |
| 322A_00966 | 4832.90489 | 0.608567574 | 0.078671322 | 7.73557069 | 1.03E-14 | 2.37E-14 | Upregulated |
| 322A_01554 | 8821.208649 | -1.403164908 | 0.181789147 | -7.718639593 | 1.18E-14 | 2.70E-14 | Downregulated |
| 322A_00026 | 2782.572004 | 1.516887865 | 0.197251571 | 7.690118034 | 1.47E-14 | 3.37E-14 | Upregulated |
| 322A_00092 | 7804.7953 | -1.014473491 | 0.132310803 | -7.667351954 | 1.76E-14 | 4.02E-14 | Downregulated |
| 322A_00119 | 1497.336411 | 0.706423985 | 0.092156932 | 7.665446031 | 1.78E-14 | 4.07E-14 | Upregulated |
| 322A_00615 | 22597.51282 | -1.543976958 | 0.201805843 | -7.650804037 | 2.00E-14 | 4.56E-14 | Downregulated |
| 322A_00330 | 3211.526607 | -1.974286959 | 0.258268171 | -7.644329353 | 2.10E-14 | 4.78E-14 | Downregulated |
| 322A_00212 | 8840.493668 | -0.811982529 | 0.106231816 | -7.643496624 | 2.11E-14 | 4.81E-14 | Downregulated |
| 322A_00699 | 26090.89061 | -1.324224202 | 0.1737704 | -7.620539519 | 2.53E-14 | 5.74E-14 | Downregulated |
| 322A_00209 | 7685.954731 | -1.771525451 | 0.232671176 | -7.613858678 | 2.66E-14 | 6.03E-14 | Downregulated |
| 322A_01153 | 4502.281444 | -0.731895467 | 0.096187939 | -7.609014998 | 2.76E-14 | 6.26E-14 | Downregulated |
| 322A_01569 | 826.5094923 | 1.947234288 | 0.256206885 | 7.600241845 | 2.96E-14 | 6.69E-14 | Upregulated |
| 322A_01441 | 2276.405882 | -0.998551226 | 0.131460321 | -7.595837423 | 3.06E-14 | 6.91E-14 | Downregulated |
| 322A_01077 | 18960.59578 | -1.592348035 | 0.2098334 | -7.588629994 | 3.23E-14 | 7.29E-14 | Downregulated |
| 322A_01405 | 8815.090578 | -0.532530741 | 0.070285502 | -7.576679824 | 3.55E-14 | 7.98E-14 | Downregulated |
| 322A_00068 | 1233.489774 | 1.502547192 | 0.198529661 | 7.568376362 | 3.78E-14 | 8.50E-14 | Upregulated |
| 322A_01412 | 1474.579422 | -1.673012798 | 0.222811483 | -7.508647102 | 5.97E-14 | 1.34E-13 | Downregulated |
| 322A_00448 | 364.5180267 | 1.764975724 | 0.23544656 | 7.496290131 | 6.56E-14 | 1.47E-13 | Upregulated |
| 322A_01452 | 19469.12529 | -1.245016988 | 0.166201995 | -7.490987027 | 6.84E-14 | 1.53E-13 | Downregulated |
| 322A_00452 | 1632.509074 | 1.543951839 | 0.206284419 | 7.484578078 | 7.18E-14 | 1.61E-13 | Upregulated |
| 322A_01008 | 8927.394353 | 0.917957517 | 0.122800121 | 7.475216709 | 7.71E-14 | 1.72E-13 | Upregulated |
| 322A_00809 | 3942.107244 | 1.409057822 | 0.189007034 | 7.455054934 | 8.98E-14 | 2.00E-13 | Upregulated |
| 322A_00601 | 2926.304882 | 0.95594171 | 0.128938686 | 7.41392469 | 1.23E-13 | 2.73E-13 | Upregulated |

| GeneID | Base mean | log2(FC) | StdErr | Wald-Stats | P-value | P-adj_BH | Effect of menadione on gene expression |
|---|---|---|---|---|---|---|---|
| 322A_00719 | 4039.411904 | 0.816367411 | 0.110193964 | 7.408458483 | 1.28E-13 | 2.84E-13 | Upregulated |
| 322A_01386 | 3679.915986 | 0.768646359 | 0.104029772 | 7.388715203 | 1.48E-13 | 3.29E-13 | Upregulated |
| 322A_01307 | 5914.31836 | -1.028588216 | 0.139409368 | -7.378185779 | 1.60E-13 | 3.56E-13 | Downregulated |
| 322A_00353 | 3442.086156 | 0.48203625 | 0.065504225 | 7.358857374 | 1.85E-13 | 4.11E-13 | Upregulated |
| 322A_00261 | 35466.00567 | -1.568154702 | 0.214366063 | -7.315312311 | 2.57E-13 | 5.68E-13 | Downregulated |
| 322A_00687 | 2328.22572 | -0.73417041 | 0.100410957 | -7.311656342 | 2.64E-13 | 5.83E-13 | Downregulated |
| 322A_00098 | 339.7174768 | -2.565120087 | 0.351263649 | -7.302549231 | 2.82E-13 | 6.23E-13 | Downregulated |
| 322A_00996 | 4239.462564 | -0.750919978 | 0.102846352 | -7.301376878 | 2.85E-13 | 6.27E-13 | Downregulated |
| 322A_00815 | 15729.62697 | 0.995270536 | 0.136769822 | 7.27697469 | 3.41E-13 | 7.51E-13 | Upregulated |
| 322A_00140 | 4373.103563 | 1.034982379 | 0.142320507 | 7.272194301 | 3.54E-13 | 7.77E-13 | Upregulated |
| 322A_00305 | 2072.070234 | -1.154459893 | 0.158830728 | -7.268492102 | 3.64E-13 | 7.96E-13 | Downregulated |
| 322A_00396 | 68814.14288 | -1.476261481 | 0.203100672 | -7.268619373 | 3.63E-13 | 7.96E-13 | Downregulated |
| 322A_00552 | 4191.719093 | -0.867904956 | 0.119502967 | -7.262622676 | 3.80E-13 | 8.30E-13 | Downregulated |
| 322A_01206 | 13385.77894 | 0.859708039 | 0.118456021 | 7.257613706 | 3.94E-13 | 8.60E-13 | Upregulated |
| 322A_00292 | 184593.9014 | -1.640866568 | 0.226444775 | -7.246210769 | 4.29E-13 | 9.35E-13 | Downregulated |
| 322A_00007 | 23043.7212 | -1.480144469 | 0.204427558 | -7.240435133 | 4.47E-13 | 9.74E-13 | Downregulated |
| 322A_00500 | 269.119601 | 2.78669523 | 0.386081056 | 7.217902012 | 5.28E-13 | 1.15E-12 | Upregulated |
| 322A_00749 | 3132.059333 | -0.670858246 | 0.093229469 | -7.19577462 | 6.21E-13 | 1.35E-12 | Downregulated |
| 322A_01419 | 596.4387107 | 1.118121588 | 0.155539376 | 7.1886722 | 6.54E-13 | 1.42E-12 | Upregulated |
| 322A_00191 | 5285.988496 | -0.739611576 | 0.103372259 | -7.15483615 | 8.38E-13 | 1.81E-12 | Downregulated |
| 322A_00349 | 2634.426021 | -0.74819257 | 0.10483943 | -7.136556986 | 9.57E-13 | 2.07E-12 | Downregulated |
| 322A_00079 | 17351.92927 | -1.119266709 | 0.15716129 | -7.121770968 | 1.07E-12 | 2.30E-12 | Downregulated |
| 322A_01374 | 27402.8972 | -1.286530012 | 0.181102076 | -7.103894339 | 1.21E-12 | 2.62E-12 | Downregulated |
| 322A_00236 | 4927.55485 | -0.393228375 | 0.055411456 | -7.096517587 | 1.28E-12 | 2.76E-12 | Downregulated |
| 322A_00486 | 3554.654811 | 0.979725895 | 0.139038189 | 7.046451791 | 1.84E-12 | 3.95E-12 | Upregulated |
| 322A_00053 | 14009.63692 | -1.141568541 | 0.162324645 | -7.032626124 | 2.03E-12 | 4.35E-12 | Downregulated |
| 322A_00171 | 965.6710319 | -1.260517587 | 0.179319268 | -7.029459789 | 2.07E-12 | 4.45E-12 | Downregulated |
| 322A_01087 | 4690.50235 | -1.214718514 | 0.173029238 | -7.020307826 | 2.21E-12 | 4.74E-12 | Downregulated |
| 322A_00856 | 7364.33745 | 1.82824385 | 0.260975339 | 7.005427615 | 2.46E-12 | 5.27E-12 | Upregulated |
| 322A_01256 | 5390.182407 | -0.809715266 | 0.115821182 | -6.991081008 | 2.73E-12 | 5.83E-12 | Downregulated |
| 322A_01402 | 6560.648964 | -1.473633707 | 0.211391055 | -6.971126133 | 3.14E-12 | 6.71E-12 | Downregulated |
| 322A_00419 | 316.3542764 | 1.898293843 | 0.272389545 | 6.969040777 | 3.19E-12 | 6.80E-12 | Upregulated |
| 322A_01584 | 721.7303864 | -2.993811005 | 0.430074959 | -6.961137688 | 3.38E-12 | 7.18E-12 | Downregulated |
| 322A_00546 | 10121.08349 | 0.853998939 | 0.123231825 | 6.930019397 | 4.21E-12 | 8.94E-12 | Upregulated |
| 322A_00984 | 11759.90515 | -2.371004689 | 0.342275201 | -6.927188077 | 4.29E-12 | 9.11E-12 | Downregulated |
| 322A_00468 | 2773.533992 | -1.153877109 | 0.166836131 | -6.916230322 | 4.64E-12 | 9.83E-12 | Downregulated |
| 322A_00214 | 1675.138375 | -1.590865098 | 0.230172187 | -6.911630446 | 4.79E-12 | 1.01E-11 | Downregulated |
| 322A_01458 | 1341.946899 | 1.84366487 | 0.267447084 | 6.893568781 | 5.44E-12 | 1.15E-11 | Upregulated |
| 322A_00756 | 2614.462327 | -0.633610829 | 0.091949047 | -6.890890658 | 5.54E-12 | 1.17E-11 | Downregulated |
| 322A_01209 | 3659.549874 | -0.99247137 | 0.144894361 | -6.849620417 | 7.40E-12 | 1.56E-11 | Downregulated |
| 322A_01098 | 4465.173018 | -1.487229967 | 0.218193828 | -6.816095476 | 9.35E-12 | 1.97E-11 | Downregulated |
| 322A_01253 | 4117.371794 | 0.693700317 | 0.102099099 | 6.794382323 | 1.09E-11 | 2.29E-11 | Upregulated |

| GeneID | Base mean | log2(FC) | StdErr | Wald-Stats | P-value | P-adj_BH | Effect of menadione on gene expression |
|---|---|---|---|---|---|---|---|
| 322A_00120 | 2930.346715 | 0.822960868 | 0.121259157 | 6.78679359 | 1.15E-11 | 2.41E-11 | Upregulated |
| 322A_00915 | 2636.962326 | -0.742738405 | 0.109549761 | -6.779918096 | 1.20E-11 | 2.52E-11 | Downregulated |
| 322A_01219 | 1406.788969 | 1.724973553 | 0.255397948 | 6.75406192 | 1.44E-11 | 3.01E-11 | Upregulated |
| 322A_00258 | 3687.605017 | 1.107842462 | 0.164690222 | 6.726825965 | 1.73E-11 | 3.63E-11 | Upregulated |
| 322A_00362 | 2344.208572 | 0.654476706 | 0.097522115 | 6.711059414 | 1.93E-11 | 4.04E-11 | Upregulated |
| 322A_00530 | 889.2181606 | 1.383890145 | 0.206818669 | 6.691321216 | 2.21E-11 | 4.61E-11 | Upregulated |
| 322A_00255 | 3206.636368 | 0.686073453 | 0.1025768 | 6.688388139 | 2.26E-11 | 4.70E-11 | Upregulated |
| 322A_00623 | 1316.652074 | 1.681747992 | 0.251495934 | 6.686978843 | 2.28E-11 | 4.74E-11 | Upregulated |
| 322A_01101 | 10397.72836 | -0.554996433 | 0.083452492 | -6.650447703 | 2.92E-11 | 6.07E-11 | Downregulated |
| 322A_00420 | 546.6261368 | 0.814362662 | 0.122724281 | 6.635709359 | 3.23E-11 | 6.70E-11 | Upregulated |
| 322A_00510 | 52.67768476 | -1.866653683 | 0.281436532 | -6.632591978 | 3.30E-11 | 6.83E-11 | Downregulated |
| 322A_01152 | 4577.323232 | 0.631400434 | 0.095328941 | 6.623386676 | 3.51E-11 | 7.26E-11 | Upregulated |
| 322A_01158 | 707.455098 | 1.931935835 | 0.291757539 | 6.621716929 | 3.55E-11 | 7.34E-11 | Upregulated |
| 322A_01235 | 13507.4172 | -1.13986129 | 0.172278938 | -6.616370524 | 3.68E-11 | 7.60E-11 | Downregulated |
| 322A_00012 | 3589.22876 | -2.026797192 | 0.307763602 | -6.585564958 | 4.53E-11 | 9.34E-11 | Downregulated |
| 322A_01050 | 17809.86555 | -0.878192563 | 0.133628755 | -6.571883142 | 4.97E-11 | 1.02E-10 | Downregulated |
| 322A_00991 | 2266.080479 | -2.936571183 | 0.447270362 | -6.565539403 | 5.18E-11 | 1.07E-10 | Downregulated |
| 322A_00777 | 12004.70402 | -0.638492955 | 0.097531573 | -6.546525772 | 5.89E-11 | 1.21E-10 | Downregulated |
| 322A_00875 | 3420.152257 | 0.902699949 | 0.137981477 | 6.542182108 | 6.06E-11 | 1.24E-10 | Upregulated |
| 322A_01090 | 20640.9698 | -0.98596459 | 0.150713284 | -6.541988647 | 6.07E-11 | 1.24E-10 | Downregulated |
| 322A_00409 | 949.4637008 | -0.509511655 | 0.078077626 | -6.525706319 | 6.77E-11 | 1.38E-10 | Downregulated |
| 322A_00350 | 118095.2567 | -1.76277524 | 0.270185271 | -6.524320274 | 6.83E-11 | 1.40E-10 | Downregulated |
| 322A_00162 | 4512.566677 | -1.224830205 | 0.187931068 | -6.517443957 | 7.15E-11 | 1.46E-10 | Downregulated |
| 322A_00906 | 5725.224686 | -1.672938935 | 0.257040305 | -6.5084693 | 7.59E-11 | 1.55E-10 | Downregulated |
| 322A_00411 | 3823.785015 | -1.291001286 | 0.198428333 | -6.506133819 | 7.71E-11 | 1.57E-10 | Downregulated |
| 322A_00596 | 2230.107063 | -1.26261945 | 0.194287633 | -6.498712412 | 8.10E-11 | 1.65E-10 | Downregulated |
| 322A_00022 | 3903.512329 | -0.512885258 | 0.079082668 | -6.485431886 | 8.85E-11 | 1.80E-10 | Downregulated |
| 322A_01555 | 3796.638005 | -1.265026125 | 0.195329125 | -6.476382481 | 9.39E-11 | 1.90E-10 | Downregulated |
| 322A_01271 | 206.2229825 | 1.745383002 | 0.270463092 | 6.453313047 | 1.09E-10 | 2.22E-10 | Upregulated |
| 322A_00415 | 3459.61327 | 1.273794694 | 0.197525212 | 6.448770167 | 1.13E-10 | 2.28E-10 | Upregulated |
| 322A_01063 | 1220.960947 | -0.515856777 | 0.080190491 | -6.432892074 | 1.25E-10 | 2.53E-10 | Downregulated |
| 322A_00748 | 1541.218404 | 0.51374682 | 0.079988189 | 6.422783524 | 1.34E-10 | 2.70E-10 | Upregulated |
| 322A_01600 | 429647.5734 | -1.399672625 | 0.218087991 | -6.41792618 | 1.38E-10 | 2.78E-10 | Downregulated |
| 322A_00029 | 425.6774436 | -1.195079247 | 0.186255111 | -6.416356782 | 1.40E-10 | 2.81E-10 | Downregulated |
| 322A_00084 | 1603.344569 | 0.838079646 | 0.130767478 | 6.408930239 | 1.47E-10 | 2.94E-10 | Upregulated |
| 322A_00086 | 7891.265165 | -1.187024721 | 0.185531144 | -6.39798093 | 1.57E-10 | 3.16E-10 | Downregulated |
| 322A_00382 | 2299.688714 | 1.088374001 | 0.170956807 | 6.366368312 | 1.94E-10 | 3.88E-10 | Upregulated |
| 322A_01230 | 3886.362488 | -0.56291906 | 0.088460074 | -6.363538236 | 1.97E-10 | 3.95E-10 | Downregulated |
| 322A_01518 | 5538.800581 | -0.501485791 | 0.078951235 | -6.351842257 | 2.13E-10 | 4.25E-10 | Downregulated |
| 322A_00476 | 224.1624774 | -0.917627603 | 0.144502474 | -6.350255313 | 2.15E-10 | 4.29E-10 | Downregulated |
| 322A_01137 | 7549.080854 | -1.24354208 | 0.196439404 | -6.330410564 | 2.45E-10 | 4.87E-10 | Downregulated |
| 322A_00488 | 11490.75513 | -0.531660905 | 0.084519015 | -6.290429495 | 3.17E-10 | 6.30E-10 | Downregulated |

| GeneID | Base mean | log2(FC) | StdErr | Wald-Stats | P-value | P-adj_BH | Effect of menadione on gene expression |
|---|---|---|---|---|---|---|---|
| 322A_00170 | 11411.89045 | -1.151801546 | 0.183171618 | -6.288100505 | 3.21E-10 | 6.39E-10 | Downregulated |
| 322A_00322 | 4214.968547 | -1.107385637 | 0.176200372 | -6.284808745 | 3.28E-10 | 6.52E-10 | Downregulated |
| 322A_00925 | 137.8654391 | 1.01651021 | 0.162710263 | 6.247363828 | 4.17E-10 | 8.28E-10 | Upregulated |
| 322A_01095 | 43534.81408 | -0.958391606 | 0.153630221 | -6.23830129 | 4.42E-10 | 8.76E-10 | Downregulated |
| 322A_00628 | 2070.444388 | 0.759622338 | 0.121846091 | 6.234277461 | 4.54E-10 | 8.98E-10 | Upregulated |
| 322A_01204 | 7549.188405 | -1.439790593 | 0.231025035 | -6.232184277 | 4.60E-10 | 9.09E-10 | Downregulated |
| 322A_01539 | 102153.4243 | -1.388353983 | 0.223376116 | -6.215319745 | 5.12E-10 | 1.01E-09 | Downregulated |
| 322A_00104 | 3633.854164 | 0.820216638 | 0.132248686 | 6.202077804 | 5.57E-10 | 1.10E-09 | Upregulated |
| 322A_00599 | 1848.075157 | 1.183409217 | 0.190845615 | 6.200871932 | 5.62E-10 | 1.11E-09 | Upregulated |
| 322A_01487 | 781.6413749 | 1.154513882 | 0.186421233 | 6.19303855 | 5.90E-10 | 1.16E-09 | Upregulated |
| 322A_01500 | 1299.737992 | -0.579203431 | 0.093785631 | -6.17582274 | 6.58E-10 | 1.29E-09 | Downregulated |
| 322A_00588 | 7606.417884 | -1.036217866 | 0.167796062 | -6.175459994 | 6.60E-10 | 1.29E-09 | Downregulated |
| 322A_01369 | 1470.416294 | 1.603928263 | 0.260036003 | 6.168100733 | 6.91E-10 | 1.35E-09 | Upregulated |
| 322A_00383 | 6646.685063 | 0.922618791 | 0.149648412 | 6.165242787 | 7.04E-10 | 1.38E-09 | Upregulated |
| 322A_00607 | 63196.48038 | -1.539051947 | 0.250296322 | -6.148919545 | 7.80E-10 | 1.52E-09 | Downregulated |
| 322A_01182 | 3933.071284 | 0.45478551 | 0.074055257 | 6.14116441 | 8.19E-10 | 1.60E-09 | Upregulated |
| 322A_01424 | 2018.514589 | 0.984307634 | 0.16045638 | 6.134425033 | 8.55E-10 | 1.67E-09 | Upregulated |
| 322A_00708 | 3094.593236 | 1.150333875 | 0.187697209 | 6.128667974 | 8.86E-10 | 1.73E-09 | Upregulated |
| 322A_00352 | 137.4863233 | 1.24640348 | 0.203494111 | 6.12501007 | 9.07E-10 | 1.76E-09 | Upregulated |
| 322A_00157 | 34508.18845 | -1.21669929 | 0.198753044 | -6.121663686 | 9.26E-10 | 1.80E-09 | Downregulated |
| 322A_01276 | 491.1188798 | -2.387180781 | 0.390005868 | -6.120884259 | 9.31E-10 | 1.81E-09 | Downregulated |
| 322A_00195 | 3202.189584 | 0.544676777 | 0.089058059 | 6.115974009 | 9.60E-10 | 1.86E-09 | Upregulated |
| 322A_00561 | 108.5340631 | 1.713533341 | 0.2801819 | 6.115788865 | 9.61E-10 | 1.86E-09 | Upregulated |
| 322A_00031 | 96348.66906 | -1.822463141 | 0.298087422 | -6.113854554 | 9.73E-10 | 1.88E-09 | Downregulated |
| 322A_01310 | 1498.862605 | 0.911403546 | 0.14938254 | 6.101138355 | 1.05E-09 | 2.03E-09 | Upregulated |
| 322A_01379 | 635.1768689 | 0.923464806 | 0.152100273 | 6.07142111 | 1.27E-09 | 2.44E-09 | Upregulated |
| 322A_00644 | 2561.18435 | 0.513607136 | 0.084682959 | 6.065058947 | 1.32E-09 | 2.54E-09 | Upregulated |
| 322A_00381 | 5203.43904 | 0.51878833 | 0.085787638 | 6.047355353 | 1.47E-09 | 2.83E-09 | Upregulated |
| 322A_01037 | 465.0811402 | 0.665310066 | 0.11018786 | 6.037961591 | 1.56E-09 | 3.00E-09 | Upregulated |
| 322A_00576 | 2723.115129 | 0.513630375 | 0.085364491 | 6.016908991 | 1.78E-09 | 3.41E-09 | Upregulated |
| 322A_00334 | 3847.869028 | 1.823934401 | 0.303511081 | 6.009449126 | 1.86E-09 | 3.57E-09 | Upregulated |
| 322A_00308 | 1938.800562 | 0.896207541 | 0.149959263 | 5.976339984 | 2.28E-09 | 4.37E-09 | Upregulated |
| 322A_01150 | 6220.702022 | -0.724712676 | 0.121348582 | -5.972156125 | 2.34E-09 | 4.48E-09 | Downregulated |
| 322A_00093 | 3196.574395 | -0.403940994 | 0.067673917 | -5.968931792 | 2.39E-09 | 4.56E-09 | Downregulated |
| 322A_00799 | 5733.563543 | 1.346428348 | 0.225592652 | 5.968405167 | 2.40E-09 | 4.57E-09 | Upregulated |
| 322A_00858 | 1803.629928 | 1.188830294 | 0.199251676 | 5.966475756 | 2.42E-09 | 4.62E-09 | Upregulated |
| 322A_01607 | 1109.552044 | -2.245693563 | 0.376748152 | -5.960728815 | 2.51E-09 | 4.78E-09 | Downregulated |
| 322A_00167 | 1064.360498 | 1.792883011 | 0.301829259 | 5.940057022 | 2.85E-09 | 5.41E-09 | Upregulated |
| 322A_00150 | 1966.884593 | 0.985864584 | 0.166562999 | 5.918869088 | 3.24E-09 | 6.15E-09 | Upregulated |
| 322A_00473 | 20002.18582 | -1.219353898 | 0.206096569 | -5.916420179 | 3.29E-09 | 6.24E-09 | Downregulated |
| 322A_00326 | 5364.920331 | -1.241944525 | 0.210663138 | -5.895405033 | 3.74E-09 | 7.08E-09 | Downregulated |
| 322A_00039 | 26.69500917 | -2.005548724 | 0.340737687 | -5.88590227 | 3.96E-09 | 7.49E-09 | Downregulated |

| GeneID | Base mean | log2(FC) | StdErr | Wald-Stats | P-value | P-adj_BH | Effect of menadione on gene expression |
|---|---|---|---|---|---|---|---|
| 322A_00930 | 22.14230676 | 2.506860822 | 0.426834904 | 5.873139239 | 4.28E-09 | 8.08E-09 | Upregulated |
| 322A_00224 | 18748.41502 | -1.222498301 | 0.209749393 | -5.828375879 | 5.60E-09 | 1.06E-08 | Downregulated |
| 322A_00240 | 5286.416288 | 0.523890346 | 0.090081351 | 5.815747008 | 6.04E-09 | 1.14E-08 | Upregulated |
| 322A_01108 | 627.6937046 | 0.950519946 | 0.163434018 | 5.815924714 | 6.03E-09 | 1.14E-08 | Upregulated |
| 322A_01045 | 2065.207633 | 1.770047313 | 0.305375632 | 5.796295204 | 6.78E-09 | 1.27E-08 | Upregulated |
| 322A_00331 | 1804.532187 | -0.979406153 | 0.169011165 | -5.794919855 | 6.84E-09 | 1.28E-08 | Downregulated |
| 322A_00310 | 1590.44051 | 1.693430731 | 0.292314208 | 5.793186525 | 6.91E-09 | 1.30E-08 | Upregulated |
| 322A_00348 | 5430.097435 | -0.707791747 | 0.122315023 | -5.7866297 | 7.18E-09 | 1.35E-08 | Downregulated |
| 322A_01504 | 677.888617 | 1.756474066 | 0.30414368 | 5.775145702 | 7.69E-09 | 1.44E-08 | Upregulated |
| 322A_00524 | 1147.449346 | 2.67778974 | 0.464031482 | 5.770707037 | 7.89E-09 | 1.48E-08 | Upregulated |
| 322A_01193 | 2325.036141 | -1.050230212 | 0.182115601 | -5.766832737 | 8.08E-09 | 1.51E-08 | Downregulated |
| 322A_01222 | 3854.44683 | -1.130877758 | 0.196229716 | -5.76303009 | 8.26E-09 | 1.54E-08 | Downregulated |
| 322A_01498 | 1361.870611 | -1.103802779 | 0.192017961 | -5.748435053 | 9.01E-09 | 1.68E-08 | Downregulated |
| 322A_00312 | 692.9387779 | 0.847876639 | 0.147577252 | 5.745307152 | 9.18E-09 | 1.71E-08 | Upregulated |
| 322A_00244 | 2251.550265 | -1.073830336 | 0.187542898 | -5.725785126 | 1.03E-08 | 1.91E-08 | Downregulated |
| 322A_01485 | 23480.76536 | -1.336537141 | 0.233585689 | -5.721828023 | 1.05E-08 | 1.96E-08 | Downregulated |
| 322A_01240 | 3727.406703 | -0.880444653 | 0.153925947 | -5.719923574 | 1.07E-08 | 1.98E-08 | Downregulated |
| 322A_00472 | 32458.19924 | -0.931941728 | 0.163228905 | -5.70941605 | 1.13E-08 | 2.10E-08 | Downregulated |
| 322A_00478 | 5409.896742 | -0.878885683 | 0.154009917 | -5.70668241 | 1.15E-08 | 2.13E-08 | Downregulated |
| 322A_00837 | 4328.589924 | 1.409593264 | 0.247774003 | 5.689028098 | 1.28E-08 | 2.36E-08 | Upregulated |
| 322A_00469 | 5801.635338 | 0.925709789 | 0.162964942 | 5.680422908 | 1.34E-08 | 2.48E-08 | Upregulated |
| 322A_01422 | 2686.435415 | 0.618203227 | 0.108945244 | 5.674439801 | 1.39E-08 | 2.56E-08 | Upregulated |
| 322A_00558 | 49.23315952 | -1.311125687 | 0.231441481 | -5.665041907 | 1.47E-08 | 2.71E-08 | Downregulated |
| 322A_00289 | 4658.31386 | 0.945848981 | 0.167199152 | 5.657020212 | 1.54E-08 | 2.83E-08 | Upregulated |
| 322A_01180 | 1405.010347 | -0.830930957 | 0.147624053 | -5.628696276 | 1.82E-08 | 3.33E-08 | Downregulated |
| 322A_00937 | 4816.680526 | 1.209545878 | 0.215440659 | 5.614287857 | 1.97E-08 | 3.62E-08 | Upregulated |
| 322A_00274 | 3200.111955 | 1.214020234 | 0.216262885 | 5.613631913 | 1.98E-08 | 3.63E-08 | Upregulated |
| 322A_01038 | 1099.824399 | -1.185239434 | 0.211604866 | -5.601191776 | 2.13E-08 | 3.90E-08 | Downregulated |
| 322A_01015 | 7802.489068 | -0.937484738 | 0.167949598 | -5.581940927 | 2.38E-08 | 4.35E-08 | Downregulated |
| 322A_01403 | 9660.204418 | -0.9685329 | 0.17445237 | -5.551847172 | 2.83E-08 | 5.16E-08 | Downregulated |
| 322A_00506 | 4863.085122 | 1.026260041 | 0.184939948 | 5.549152868 | 2.87E-08 | 5.23E-08 | Upregulated |
| 322A_00045 | 18.58209088 | -4.085584459 | 0.737645287 | -5.538684423 | 3.05E-08 | 5.55E-08 | Downregulated |
| 322A_01306 | 1189.350908 | -0.926904822 | 0.167478947 | -5.53445575 | 3.12E-08 | 5.68E-08 | Downregulated |
| 322A_00371 | 1620.00465 | -0.615592825 | 0.111243983 | -5.533717944 | 3.14E-08 | 5.70E-08 | Downregulated |
| 322A_01014 | 3527.571702 | 0.423974305 | 0.076774378 | 5.522341131 | 3.35E-08 | 6.07E-08 | Upregulated |
| 322A_00061 | 3728.377269 | 0.89880562 | 0.163495849 | 5.497421655 | 3.85E-08 | 6.99E-08 | Upregulated |
| 322A_00531 | 3031.183276 | -1.359233368 | 0.247670145 | -5.488079185 | 4.06E-08 | 7.36E-08 | Downregulated |
| 322A_01321 | 14439.78511 | -0.626758188 | 0.11437883 | -5.479669493 | 4.26E-08 | 7.71E-08 | Downregulated |
| 322A_00759 | 606.8407757 | 0.755025718 | 0.137794437 | 5.479362825 | 4.27E-08 | 7.71E-08 | Upregulated |
| 322A_01384 | 2487.810385 | 1.140204557 | 0.208345883 | 5.472652198 | 4.43E-08 | 8.00E-08 | Upregulated |
| 322A_00893 | 5603.026143 | -1.080170195 | 0.197638138 | -5.465393489 | 4.62E-08 | 8.33E-08 | Downregulated |
| 322A_00430 | 1000.909483 | -1.507758342 | 0.276192961 | -5.459075919 | 4.79E-08 | 8.62E-08 | Downregulated |

| GeneID | Base mean | log2(FC) | StdErr | Wald-Stats | P-value | P-adj_BH | Effect of menadione on gene expression |
|---|---|---|---|---|---|---|---|
| 322A_00564 | 8501.901996 | 0.379252394 | 0.0696511 | 5.445030926 | 5.18E-08 | 9.32E-08 | Upregulated |
| 322A_01275 | 224.2695326 | 1.077294249 | 0.197882092 | 5.444121991 | 5.21E-08 | 9.35E-08 | Upregulated |
| 322A_00891 | 1010.527239 | 1.481620937 | 0.272982094 | 5.427538913 | 5.71E-08 | 1.03E-07 | Upregulated |
| 322A_00342 | 1136.285922 | 1.207342794 | 0.222528728 | 5.425559229 | 5.78E-08 | 1.04E-07 | Upregulated |
| 322A_00786 | 20.18082345 | -1.670869621 | 0.308595897 | -5.414425909 | 6.15E-08 | 1.10E-07 | Downregulated |
| 322A_01254 | 8731.635177 | -1.179062794 | 0.21802236 | -5.407990235 | 6.37E-08 | 1.14E-07 | Downregulated |
| 322A_00772 | 4740.041431 | -0.697056086 | 0.129239007 | -5.393542556 | 6.91E-08 | 1.23E-07 | Downregulated |
| 322A_01560 | 580.7396501 | -2.038332693 | 0.378877126 | -5.379930731 | 7.45E-08 | 1.33E-07 | Downregulated |
| 322A_01071 | 2693.282172 | 0.871077313 | 0.162036478 | 5.375809952 | 7.62E-08 | 1.36E-07 | Upregulated |
| 322A_00989 | 4036.253079 | 1.806605652 | 0.337094839 | 5.359339399 | 8.35E-08 | 1.49E-07 | Upregulated |
| 322A_00960 | 1400.396805 | 0.994231791 | 0.185723405 | 5.353292919 | 8.64E-08 | 1.54E-07 | Upregulated |
| 322A_00574 | 62.93381732 | 2.082124593 | 0.389301702 | 5.348357288 | 8.88E-08 | 1.58E-07 | Upregulated |
| 322A_01089 | 8138.806032 | -1.159915097 | 0.216962792 | -5.346147533 | 8.98E-08 | 1.59E-07 | Downregulated |
| 322A_00876 | 2607.037904 | -0.379062685 | 0.070958628 | -5.342023913 | 9.19E-08 | 1.63E-07 | Downregulated |
| 322A_00346 | 6341.94815 | -0.873616823 | 0.163576687 | -5.340717182 | 9.26E-08 | 1.64E-07 | Downregulated |
| 322A_01264 | 276.0137749 | -0.851073291 | 0.159389538 | -5.33958064 | 9.32E-08 | 1.65E-07 | Downregulated |
| 322A_01496 | 159.7422024 | -2.133953057 | 0.400189756 | -5.332353034 | 9.69E-08 | 1.71E-07 | Downregulated |
| 322A_00838 | 19413.98331 | 1.35389568 | 0.254036128 | 5.329539905 | 9.85E-08 | 1.74E-07 | Upregulated |
| 322A_01492 | 5784.412242 | 0.987021108 | 0.185603848 | 5.317891417 | 1.05E-07 | 1.85E-07 | Upregulated |
| 322A_01313 | 7474.520146 | -0.950545683 | 0.179078296 | -5.307989307 | 1.11E-07 | 1.95E-07 | Downregulated |
| 322A_01357 | 13595.18063 | -1.056240225 | 0.199333598 | -5.298856967 | 1.17E-07 | 2.05E-07 | Downregulated |
| 322A_01408 | 135.8331301 | -1.008027469 | 0.190631923 | -5.287820917 | 1.24E-07 | 2.17E-07 | Downregulated |
| 322A_01491 | 8713.40618 | -1.074069412 | 0.203419262 | -5.280077232 | 1.29E-07 | 2.27E-07 | Downregulated |
| 322A_00764 | 1181.337681 | 1.225067346 | 0.232041058 | 5.279528359 | 1.30E-07 | 2.27E-07 | Upregulated |
| 322A_00078 | 5585.253245 | -0.657326864 | 0.12539883 | -5.241889927 | 1.59E-07 | 2.78E-07 | Downregulated |
| 322A_00783 | 4013.876684 | 0.876224234 | 0.167463993 | 5.23231423 | 1.67E-07 | 2.93E-07 | Upregulated |
| 322A_01168 | 434.5578331 | -1.402032157 | 0.268143088 | -5.228671631 | 1.71E-07 | 2.98E-07 | Downregulated |
| 322A_00775 | 609.7855624 | 0.762289263 | 0.145904072 | 5.224592091 | 1.75E-07 | 3.05E-07 | Upregulated |
| 322A_00208 | 2462.36539 | -0.884381515 | 0.169789927 | -5.208680695 | 1.90E-07 | 3.32E-07 | Downregulated |
| 322A_00559 | 77.4548457 | -1.672094601 | 0.321594728 | -5.199384368 | 2.00E-07 | 3.48E-07 | Downregulated |
| 322A_00405 | 813.3289228 | 1.951749196 | 0.375621731 | 5.196049737 | 2.04E-07 | 3.54E-07 | Upregulated |
| 322A_01574 | 4441.641901 | -0.562689781 | 0.108293704 | -5.195960236 | 2.04E-07 | 3.54E-07 | Downregulated |
| 322A_01348 | 10621.65432 | -1.012783345 | 0.195099959 | -5.191099722 | 2.09E-07 | 3.63E-07 | Downregulated |
| 322A_00671 | 3215.741399 | 0.865769349 | 0.167198551 | 5.17809121 | 2.24E-07 | 3.89E-07 | Upregulated |
| 322A_00630 | 1759.987869 | 0.493170433 | 0.095341354 | 5.172681226 | 2.31E-07 | 4.00E-07 | Upregulated |
| 322A_01489 | 1787.135931 | -0.644029465 | 0.124679928 | -5.165462292 | 2.40E-07 | 4.15E-07 | Downregulated |
| 322A_00490 | 4081.166289 | -0.392326313 | 0.076009272 | -5.161558628 | 2.45E-07 | 4.23E-07 | Downregulated |
| 322A_00680 | 7290.435295 | -0.920748827 | 0.178550928 | -5.156785459 | 2.51E-07 | 4.34E-07 | Downregulated |
| 322A_01529 | 164.5487839 | -0.848692088 | 0.16467051 | -5.153880236 | 2.55E-07 | 4.40E-07 | Downregulated |
| 322A_00956 | 11436.07292 | -0.617162056 | 0.120000421 | -5.142999074 | 2.70E-07 | 4.66E-07 | Downregulated |
| 322A_00033 | 156289.7631 | -1.189939315 | 0.231491508 | -5.140315181 | 2.74E-07 | 4.72E-07 | Downregulated |
| 322A_00329 | 34.37109956 | -1.465871053 | 0.285177401 | -5.140207627 | 2.74E-07 | 4.72E-07 | Downregulated |

| GeneID | Base mean | log2(FC) | StdErr | Wald-Stats | P-value | P-adj_BH | Effect of menadione on gene expression |
|---|---|---|---|---|---|---|---|
| 322A_01409 | 758.5175472 | 1.556707016 | 0.30317591 | 5.13466593 | 2.83E-07 | 4.85E-07 | Upregulated |
| 322A_01499 | 2025.086836 | -0.833640942 | 0.162587055 | -5.127351272 | 2.94E-07 | 5.04E-07 | Downregulated |
| 322A_01328 | 16119.2656 | -0.43047503 | 0.084352475 | -5.103288652 | 3.34E-07 | 5.72E-07 | Downregulated |
| 322A_00316 | 2444.475011 | 1.408164629 | 0.276572199 | 5.091490163 | 3.55E-07 | 6.08E-07 | Upregulated |
| 322A_00945 | 11542.50147 | 0.606849817 | 0.119436395 | 5.080945529 | 3.76E-07 | 6.42E-07 | Upregulated |
| 322A_00637 | 5788.55245 | -0.291180665 | 0.057499829 | -5.064026601 | 4.10E-07 | 7.01E-07 | Downregulated |
| 322A_00436 | 761.2938398 | 0.720877481 | 0.142411089 | 5.061947669 | 4.15E-07 | 7.08E-07 | Upregulated |
| 322A_00793 | 4103.666477 | -1.748451613 | 0.346039894 | -5.052745781 | 4.36E-07 | 7.42E-07 | Downregulated |
| 322A_01429 | 718.8200425 | 0.58764551 | 0.116552376 | 5.041900716 | 4.61E-07 | 7.84E-07 | Upregulated |
| 322A_00284 | 257.3856865 | -0.587495819 | 0.116592304 | -5.038890221 | 4.68E-07 | 7.96E-07 | Downregulated |
| 322A_00967 | 71.54860148 | 2.356016126 | 0.467953593 | 5.034721731 | 4.79E-07 | 8.13E-07 | Upregulated |
| 322A_00207 | 381.8682356 | 0.52523361 | 0.10475512 | 5.013918249 | 5.33E-07 | 9.05E-07 | Upregulated |
| 322A_01139 | 363.2542918 | -0.979398398 | 0.195425226 | -5.011627291 | 5.40E-07 | 9.15E-07 | Downregulated |
| 322A_00833 | 2113.993756 | 1.230658906 | 0.246212249 | 4.998365893 | 5.78E-07 | 9.79E-07 | Upregulated |
| 322A_00146 | 20414.77333 | -0.912508109 | 0.182568889 | -4.998157757 | 5.79E-07 | 9.79E-07 | Downregulated |
| 322A_00551 | 8808.298358 | 0.511138241 | 0.102516256 | 4.985923792 | 6.17E-07 | 1.04E-06 | Upregulated |
| 322A_00364 | 600.9072696 | 1.000222876 | 0.200924619 | 4.978100155 | 6.42E-07 | 1.08E-06 | Upregulated |
| 322A_00418 | 1068.06034 | 0.736014236 | 0.149571732 | 4.92081109 | 8.62E-07 | 1.45E-06 | Upregulated |
| 322A_00554 | 3335.387014 | 0.795656414 | 0.161699371 | 4.92059065 | 8.63E-07 | 1.45E-06 | Upregulated |
| 322A_00765 | 962.0862062 | 0.888392481 | 0.180539538 | 4.920764129 | 8.62E-07 | 1.45E-06 | Upregulated |
| 322A_00230 | 4144.372107 | 0.661574132 | 0.134656961 | 4.913033289 | 8.97E-07 | 1.51E-06 | Upregulated |
| 322A_00425 | 1182.646466 | 1.190904913 | 0.242456549 | 4.911828188 | 9.02E-07 | 1.51E-06 | Upregulated |
| 322A_01294 | 634.4240305 | 1.76958339 | 0.363184721 | 4.87240594 | 1.10E-06 | 1.85E-06 | Upregulated |
| 322A_00939 | 491.1705984 | 1.4572362 | 0.299206634 | 4.870333848 | 1.11E-06 | 1.87E-06 | Upregulated |
| 322A_01503 | 894.3726877 | 0.967578453 | 0.198871024 | 4.865356626 | 1.14E-06 | 1.91E-06 | Upregulated |
| 322A_01231 | 23359.18889 | -1.080323807 | 0.22208515 | -4.864457649 | 1.15E-06 | 1.92E-06 | Downregulated |
| 322A_01249 | 843.3840251 | 0.702259213 | 0.144475312 | 4.860755806 | 1.17E-06 | 1.95E-06 | Upregulated |
| 322A_00653 | 2255.392075 | -0.70421879 | 0.144891841 | -4.860306717 | 1.17E-06 | 1.95E-06 | Downregulated |
| 322A_00309 | 934.3299964 | 0.805168801 | 0.165806453 | 4.856076388 | 1.20E-06 | 1.99E-06 | Upregulated |
| 322A_00393 | 1591.83759 | -1.868183847 | 0.385122363 | -4.850883842 | 1.23E-06 | 2.05E-06 | Downregulated |
| 322A_01516 | 4147.749192 | 0.706889479 | 0.145738267 | 4.850404052 | 1.23E-06 | 2.05E-06 | Upregulated |
| 322A_01088 | 1012.268718 | -1.489774052 | 0.307924493 | -4.83811482 | 1.31E-06 | 2.18E-06 | Downregulated |
| 322A_01154 | 32455.63808 | 0.53776918 | 0.111314324 | 4.831086961 | 1.36E-06 | 2.25E-06 | Upregulated |
| 322A_00914 | 21740.661 | -1.365076802 | 0.283036711 | -4.822967294 | 1.41E-06 | 2.34E-06 | Downregulated |
| 322A_00820 | 70.5463217 | -2.013087697 | 0.417542045 | -4.821281404 | 1.43E-06 | 2.36E-06 | Downregulated |
| 322A_00101 | 1129.543067 | -1.496363657 | 0.310736588 | -4.815537379 | 1.47E-06 | 2.43E-06 | Downregulated |
| 322A_01035 | 3262.859693 | -0.664260358 | 0.138235425 | -4.805283166 | 1.55E-06 | 2.55E-06 | Downregulated |
| 322A_00152 | 1654.970879 | 1.428067648 | 0.297616889 | 4.798342105 | 1.60E-06 | 2.64E-06 | Upregulated |
| 322A_00458 | 1348.965966 | 0.888402716 | 0.1851599 | 4.798029781 | 1.60E-06 | 2.64E-06 | Upregulated |
| 322A_00918 | 740.8009715 | 0.703137611 | 0.147054134 | 4.78148823 | 1.74E-06 | 2.87E-06 | Upregulated |
| 322A_01030 | 205938.9707 | -1.154208233 | 0.241407065 | -4.781170062 | 1.74E-06 | 2.87E-06 | Downregulated |
| 322A_00897 | 19925.59557 | -0.667891296 | 0.13981904 | -4.776826512 | 1.78E-06 | 2.93E-06 | Downregulated |

| GeneID | Base mean | log2(FC) | StdErr | Wald-Stats | P-value | P-adj_BH | Effect of menadione on gene expression |
|---|---|---|---|---|---|---|---|
| 322A_01023 | 18169.41557 | 0.983712582 | 0.206007443 | 4.775131273 | 1.80E-06 | 2.95E-06 | Upregulated |
| 322A_00318 | 5319.319872 | 0.330163251 | 0.06922224 | 4.769612327 | 1.85E-06 | 3.03E-06 | Upregulated |
| 322A_00493 | 120.1990194 | 2.057727294 | 0.431488435 | 4.768904855 | 1.85E-06 | 3.03E-06 | Upregulated |
| 322A_00028 | 2964.043334 | -0.295819601 | 0.062049377 | -4.767487065 | 1.87E-06 | 3.05E-06 | Downregulated |
| 322A_00395 | 5137.154316 | -1.212326417 | 0.254714354 | -4.759552799 | 1.94E-06 | 3.17E-06 | Downregulated |
| 322A_00188 | 29280.70223 | -1.17803853 | 0.247688264 | -4.756133825 | 1.97E-06 | 3.22E-06 | Downregulated |
| 322A_00035 | 7389.467457 | 0.875050174 | 0.184025853 | 4.755039357 | 1.98E-06 | 3.24E-06 | Upregulated |
| 322A_00338 | 17090.13491 | -0.603783212 | 0.126980288 | -4.754936541 | 1.99E-06 | 3.24E-06 | Downregulated |
| 322A_00514 | 30830.75973 | -0.861317131 | 0.181323832 | -4.750159539 | 2.03E-06 | 3.31E-06 | Downregulated |
| 322A_00678 | 359.4058156 | 1.382552116 | 0.291695765 | 4.739705822 | 2.14E-06 | 3.48E-06 | Upregulated |
| 322A_00734 | 3791.902392 | 0.533766082 | 0.112762952 | 4.733523476 | 2.21E-06 | 3.59E-06 | Upregulated |
| 322A_01297 | 16433.10618 | -1.109293782 | 0.234762758 | -4.725169321 | 2.30E-06 | 3.73E-06 | Downregulated |
| 322A_00843 | 7697.226599 | 1.185837077 | 0.251793628 | 4.709559524 | 2.48E-06 | 4.03E-06 | Upregulated |
| 322A_00164 | 3082.537881 | 0.617843475 | 0.131303982 | 4.705443551 | 2.53E-06 | 4.10E-06 | Upregulated |
| 322A_01018 | 39264.3036 | 1.204754422 | 0.256367483 | 4.699326163 | 2.61E-06 | 4.22E-06 | Upregulated |
| 322A_00048 | 33.01372475 | -2.093466735 | 0.448697598 | -4.665651748 | 3.08E-06 | 4.97E-06 | Downregulated |
| 322A_00859 | 5901.37972 | 1.255189787 | 0.269750879 | 4.65314439 | 3.27E-06 | 5.28E-06 | Upregulated |
| 322A_00604 | 2450.791935 | 1.337535719 | 0.287927902 | 4.645384174 | 3.39E-06 | 5.48E-06 | Upregulated |
| 322A_00785 | 26.6799847 | 1.556010021 | 0.335093916 | 4.643504246 | 3.43E-06 | 5.52E-06 | Upregulated |
| 322A_00688 | 7974.712236 | -0.536023634 | 0.115660126 | -4.634472169 | 3.58E-06 | 5.76E-06 | Downregulated |
| 322A_00577 | 638.8343451 | 0.825747577 | 0.178488027 | 4.626347154 | 3.72E-06 | 5.99E-06 | Upregulated |
| 322A_00087 | 3740.144597 | -0.415204091 | 0.089812626 | -4.623003584 | 3.78E-06 | 6.08E-06 | Downregulated |
| 322A_00203 | 194.5181589 | 1.636365443 | 0.353997469 | 4.622534306 | 3.79E-06 | 6.08E-06 | Upregulated |
| 322A_00800 | 5015.606845 | 0.931329999 | 0.202343919 | 4.602708118 | 4.17E-06 | 6.69E-06 | Upregulated |
| 322A_00154 | 358.6180757 | -0.946071547 | 0.205730103 | -4.598605336 | 4.25E-06 | 6.81E-06 | Downregulated |
| 322A_00361 | 1892.962631 | 0.804074171 | 0.175562017 | 4.580000757 | 4.65E-06 | 7.44E-06 | Upregulated |
| 322A_01181 | 5427.467193 | -0.646068986 | 0.141068605 | -4.579821198 | 4.65E-06 | 7.44E-06 | Downregulated |
| 322A_01124 | 365.7330485 | -0.689016237 | 0.151142391 | -4.558722623 | 5.15E-06 | 8.22E-06 | Downregulated |
| 322A_01531 | 427.1990879 | 0.821550203 | 0.180432261 | 4.553233441 | 5.28E-06 | 8.43E-06 | Upregulated |
| 322A_00499 | 5779.479477 | 0.524998407 | 0.115513216 | 4.544920723 | 5.50E-06 | 8.76E-06 | Upregulated |
| 322A_01523 | 3583.540674 | -1.28123147 | 0.284138063 | -4.509186338 | 6.51E-06 | 1.04E-05 | Downregulated |
| 322A_00610 | 9114.506007 | -0.4403043 | 0.097722861 | -4.505642741 | 6.62E-06 | 1.05E-05 | Downregulated |
| 322A_00755 | 758.7579944 | 1.392917134 | 0.309352637 | 4.502683892 | 6.71E-06 | 1.07E-05 | Upregulated |
| 322A_01397 | 4119.029523 | 0.752078261 | 0.167999116 | 4.476679877 | 7.58E-06 | 1.20E-05 | Upregulated |
| 322A_00379 | 1738.469678 | 0.644315018 | 0.143997689 | 4.474481654 | 7.66E-06 | 1.21E-05 | Upregulated |
| 322A_01096 | 15438.77199 | -1.034885102 | 0.231828572 | -4.464010161 | 8.04E-06 | 1.27E-05 | Downregulated |
| 322A_00787 | 8.041872375 | -2.784936313 | 0.625749028 | -4.450564346 | 8.56E-06 | 1.36E-05 | Downregulated |
| 322A_00713 | 923.3525108 | -0.393061706 | 0.088440495 | -4.444363467 | 8.82E-06 | 1.39E-05 | Downregulated |
| 322A_01609 | 200415.5168 | -1.627137485 | 0.366112903 | -4.44435985 | 8.82E-06 | 1.39E-05 | Downregulated |
| 322A_01269 | 94.51947887 | -1.130129967 | 0.254476294 | -4.441002925 | 8.95E-06 | 1.41E-05 | Downregulated |
| 322A_01319 | 6436.623278 | -0.459904397 | 0.103654901 | -4.436880389 | 9.13E-06 | 1.44E-05 | Downregulated |
| 322A_00536 | 1253.065763 | 0.807575997 | 0.182325015 | 4.429320878 | 9.45E-06 | 1.49E-05 | Upregulated |

| GeneID | Base mean | log2(FC) | StdErr | Wald-Stats | P-value | P-adj_BH | Effect of menadione on gene expression |
|---|---|---|---|---|---|---|---|
| 322A_00860 | 539.1825844 | 1.271985008 | 0.289809015 | 4.389045691 | 1.14E-05 | 1.79E-05 | Upregulated |
| 322A_01299 | 2560.728877 | 0.276922028 | 0.063186454 | 4.382616979 | 1.17E-05 | 1.84E-05 | Upregulated |
| 322A_01205 | 6064.104503 | -0.816206559 | 0.186564688 | -4.374925232 | 1.21E-05 | 1.91E-05 | Downregulated |
| 322A_01094 | 11940.62833 | -0.602184731 | 0.137683963 | -4.373673727 | 1.22E-05 | 1.92E-05 | Downregulated |
| 322A_01025 | 15685.72598 | -0.444276619 | 0.101615701 | -4.372125722 | 1.23E-05 | 1.93E-05 | Downregulated |
| 322A_00753 | 1182.870638 | 0.649009529 | 0.148842211 | 4.360386252 | 1.30E-05 | 2.03E-05 | Upregulated |
| 322A_00761 | 1180.331795 | 1.352635445 | 0.31075657 | 4.352717125 | 1.34E-05 | 2.10E-05 | Upregulated |
| 322A_01245 | 13591.43257 | -0.711835324 | 0.163800663 | -4.345741412 | 1.39E-05 | 2.17E-05 | Downregulated |
| 322A_01156 | 9307.180089 | 0.327769035 | 0.075435027 | 4.345050956 | 1.39E-05 | 2.17E-05 | Upregulated |
| 322A_00440 | 1887.232073 | 0.671582979 | 0.154705141 | 4.341051478 | 1.42E-05 | 2.21E-05 | Upregulated |
| 322A_01611 | 5037.686549 | -0.331476253 | 0.076369429 | -4.340431233 | 1.42E-05 | 2.22E-05 | Downregulated |
| 322A_00803 | 2945.48026 | 1.293201517 | 0.298154239 | 4.337357478 | 1.44E-05 | 2.24E-05 | Upregulated |
| 322A_00495 | 1234.39012 | 1.645439764 | 0.380110326 | 4.328847838 | 1.50E-05 | 2.33E-05 | Upregulated |
| 322A_00030 | 153.7346227 | -2.742777168 | 0.634554778 | -4.322364695 | 1.54E-05 | 2.40E-05 | Downregulated |
| 322A_00433 | 6926.097049 | -1.454461119 | 0.336876611 | -4.317489169 | 1.58E-05 | 2.45E-05 | Downregulated |
| 322A_00294 | 2407.168792 | -1.377514008 | 0.319213478 | -4.315337867 | 1.59E-05 | 2.47E-05 | Downregulated |
| 322A_01028 | 262.5837041 | 1.603101794 | 0.372705509 | 4.30125597 | 1.70E-05 | 2.63E-05 | Upregulated |
| 322A_01593 | 1164.106369 | -0.468039605 | 0.108844412 | -4.300079308 | 1.71E-05 | 2.64E-05 | Downregulated |
| 322A_00714 | 4564.658462 | -0.459437668 | 0.107137968 | -4.288280568 | 1.80E-05 | 2.78E-05 | Downregulated |
| 322A_00177 | 371.8962163 | 0.830140938 | 0.194318534 | 4.272062575 | 1.94E-05 | 2.99E-05 | Upregulated |
| 322A_00498 | 8865.871985 | 1.250688734 | 0.292939369 | 4.269445712 | 1.96E-05 | 3.02E-05 | Upregulated |
| 322A_00861 | 3047.758297 | 0.925544126 | 0.217575322 | 4.253902138 | 2.10E-05 | 3.24E-05 | Upregulated |
| 322A_01100 | 1238.397422 | 0.746677145 | 0.175538032 | 4.253648832 | 2.10E-05 | 3.24E-05 | Upregulated |
| 322A_00840 | 5927.068949 | 1.217005388 | 0.286598116 | 4.246383062 | 2.17E-05 | 3.34E-05 | Upregulated |
| 322A_01056 | 1346.522005 | 0.885997442 | 0.208845563 | 4.24235703 | 2.21E-05 | 3.40E-05 | Upregulated |
| 322A_00813 | 6730.791439 | 0.795605782 | 0.187926818 | 4.233593646 | 2.30E-05 | 3.53E-05 | Upregulated |
| 322A_00072 | 696.5286771 | -0.857864967 | 0.203315254 | -4.219383191 | 2.45E-05 | 3.76E-05 | Downregulated |
| 322A_00839 | 12732.94989 | 1.001230205 | 0.237316719 | 4.218961934 | 2.45E-05 | 3.76E-05 | Upregulated |
| 322A_00257 | 3120.280744 | 0.714975316 | 0.170133723 | 4.202431501 | 2.64E-05 | 4.04E-05 | Upregulated |
| 322A_01535 | 9643.657367 | 0.601341927 | 0.143640908 | 4.186425275 | 2.83E-05 | 4.33E-05 | Upregulated |
| 322A_00004 | 5561.243151 | -0.748633941 | 0.178928047 | -4.183994357 | 2.86E-05 | 4.38E-05 | Downregulated |
| 322A_00841 | 8524.325568 | 0.733700284 | 0.176295516 | 4.161763733 | 3.16E-05 | 4.82E-05 | Upregulated |
| 322A_01116 | 24.59488411 | -1.541880766 | 0.371364464 | -4.151934056 | 3.30E-05 | 5.03E-05 | Downregulated |
| 322A_00226 | 1680.935531 | 0.928199971 | 0.224682839 | 4.131156505 | 3.61E-05 | 5.50E-05 | Upregulated |
| 322A_01483 | 783.566113 | 1.326420225 | 0.321134168 | 4.130423841 | 3.62E-05 | 5.51E-05 | Upregulated |
| 322A_00156 | 5619.11355 | -0.904613863 | 0.219510796 | -4.121044974 | 3.77E-05 | 5.73E-05 | Downregulated |
| 322A_01335 | 125.1419281 | -1.321577215 | 0.320800667 | -4.119621162 | 3.79E-05 | 5.76E-05 | Downregulated |
| 322A_01244 | 15363.21757 | 0.700273613 | 0.170037469 | 4.118348851 | 3.82E-05 | 5.79E-05 | Upregulated |
| 322A_01117 | 171.2577719 | -0.793577497 | 0.193322016 | -4.104951486 | 4.04E-05 | 6.13E-05 | Downregulated |
| 322A_00414 | 1500.749615 | 0.720388713 | 0.175764168 | 4.09860964 | 4.16E-05 | 6.29E-05 | Upregulated |
| 322A_00836 | 12.51957884 | 2.670722344 | 0.651796758 | 4.097477183 | 4.18E-05 | 6.32E-05 | Upregulated |
| 322A_01364 | 82.6542095 | -1.058897162 | 0.258669592 | -4.093628303 | 4.25E-05 | 6.42E-05 | Downregulated |

| GeneID | Base mean | log2(FC) | StdErr | Wald-Stats | P-value | P-adj_BH | Effect of menadione on gene expression |
|---|---|---|---|---|---|---|---|
| 322A_00138 | 2921.701009 | 0.309277238 | 0.075625736 | 4.089576573 | 4.32E-05 | 6.53E-05 | Upregulated |
| 322A_00370 | 800.3654816 | 0.931903743 | 0.228653275 | 4.07561948 | 4.59E-05 | 6.92E-05 | Upregulated |
| 322A_01317 | 652.2212338 | -0.951248843 | 0.234435565 | -4.057613195 | 4.96E-05 | 7.47E-05 | Downregulated |
| 322A_00032 | 274343.7041 | -1.01124172 | 0.249268596 | -4.05683562 | 4.97E-05 | 7.49E-05 | Downregulated |
| 322A_01001 | 1458.784583 | -1.391660693 | 0.343693281 | -4.049135582 | 5.14E-05 | 7.73E-05 | Downregulated |
| 322A_00328 | 119.3735996 | -0.633778524 | 0.156613726 | -4.04676232 | 5.19E-05 | 7.80E-05 | Downregulated |
| 322A_00701 | 13197.69173 | -0.297231081 | 0.073458031 | -4.046270746 | 5.20E-05 | 7.81E-05 | Downregulated |
| 322A_01464 | 2254.818567 | -1.156200389 | 0.286048337 | -4.041975569 | 5.30E-05 | 7.95E-05 | Downregulated |
| 322A_00635 | 1031.327402 | -1.223070682 | 0.303306476 | -4.03245818 | 5.52E-05 | 8.27E-05 | Downregulated |
| 322A_00036 | 15947.58712 | -1.000164545 | 0.248693483 | -4.021675734 | 5.78E-05 | 8.65E-05 | Downregulated |
| 322A_01113 | 41.18582026 | -1.981227274 | 0.4961624 | -3.993102409 | 6.52E-05 | 9.75E-05 | Downregulated |
| 322A_00386 | 15513.19201 | 0.280177068 | 0.070425102 | 3.978369381 | 6.94E-05 | 0.000103691 | Upregulated |
| 322A_00568 | 17063.4911 | -0.59200972 | 0.148886626 | -3.976245129 | 7.00E-05 | 0.000104423 | Downregulated |
| 322A_01603 | 2844.670429 | -1.158199592 | 0.291268898 | -3.976392952 | 7.00E-05 | 0.000104423 | Downregulated |
| 322A_00779 | 560.3981211 | 0.52571858 | 0.134172249 | 3.918236328 | 8.92E-05 | 0.000132916 | Upregulated |
| 322A_00950 | 1996.57876 | 0.949949238 | 0.243441935 | 3.902159416 | 9.53E-05 | 0.00014193 | Upregulated |
| 322A_01608 | 14.41038849 | 2.197447922 | 0.56352359 | 3.899478143 | 9.64E-05 | 0.000143376 | Upregulated |
| 322A_01360 | 26491.71682 | -0.866735532 | 0.223353673 | -3.880551956 | 0.00010422 | 0.00015486 | Downregulated |
| 322A_01305 | 14350.21087 | -0.588865439 | 0.151789971 | -3.87947527 | 0.000104682 | 0.000155401 | Downregulated |
| 322A_00598 | 2148.200132 | 0.564313172 | 0.146493817 | 3.852129635 | 0.000117095 | 0.000173665 | Upregulated |
| 322A_00808 | 1459.792101 | 0.358251866 | 0.093059197 | 3.849720168 | 0.000118253 | 0.000175218 | Upregulated |
| 322A_01339 | 1749.833277 | 0.337602348 | 0.087778566 | 3.846068162 | 0.000120028 | 0.000177682 | Upregulated |
| 322A_00507 | 12958.02515 | -0.224876422 | 0.058761656 | -3.826924515 | 0.000129754 | 0.0001919 | Downregulated |
| 322A_01526 | 191.886933 | -0.647004921 | 0.169079579 | -3.826629591 | 0.00012991 | 0.000191951 | Downregulated |
| 322A_00519 | 1396.319114 | 0.347986169 | 0.091174141 | 3.816720028 | 0.000135237 | 0.000199636 | Upregulated |
| 322A_01161 | 30538.45927 | -0.811121339 | 0.212652605 | -3.814302391 | 0.000136568 | 0.000201413 | Downregulated |
| 322A_00795 | 2564.817362 | 0.60801015 | 0.159553512 | 3.810697369 | 0.000138575 | 0.000204182 | Upregulated |
| 322A_00627 | 890.8712542 | 0.523316337 | 0.137552799 | 3.804476109 | 0.000142105 | 0.000209188 | Upregulated |
| 322A_01074 | 5278.813975 | 0.530589371 | 0.13947304 | 3.804243241 | 0.000142238 | 0.00020919 | Upregulated |
| 322A_00501 | 1085.964943 | 0.46031759 | 0.121028303 | 3.803387968 | 0.000142731 | 0.000209718 | Upregulated |
| 322A_01002 | 65.07280838 | -1.169933577 | 0.307966829 | -3.798894768 | 0.000145343 | 0.000213358 | Downregulated |
| 322A_00935 | 10187.60967 | -0.625487335 | 0.164669276 | -3.798445886 | 0.000145606 | 0.000213547 | Downregulated |
| 322A_00027 | 7901.281667 | -0.910190928 | 0.240063882 | -3.791453009 | 0.000149768 | 0.000219448 | Downregulated |
| 322A_00527 | 3433.535255 | 0.344418763 | 0.090870886 | 3.79019922 | 0.000150527 | 0.000220354 | Upregulated |
| 322A_00332 | 867.0244587 | 0.571172937 | 0.151079852 | 3.78060298 | 0.000156449 | 0.000228812 | Upregulated |
| 322A_00758 | 2840.651397 | 0.434322069 | 0.115209728 | 3.769838508 | 0.000163353 | 0.000238689 | Upregulated |
| 322A_01507 | 2614.088663 | 1.232660132 | 0.328590699 | 3.751354295 | 0.000175882 | 0.000256758 | Upregulated |
| 322A_01342 | 8306.985214 | 0.498921982 | 0.13305898 | 3.749630285 | 0.000177095 | 0.000258291 | Upregulated |
| 322A_00640 | 5001.850699 | 0.546167937 | 0.145669228 | 3.749370702 | 0.000177279 | 0.000258321 | Upregulated |
| 322A_00477 | 9625.55198 | -1.378435151 | 0.368476472 | -3.740904115 | 0.000183359 | 0.000266935 | Downregulated |
| 322A_00565 | 6771.692417 | -0.967128259 | 0.258928459 | -3.735117661 | 0.000187627 | 0.000272897 | Downregulated |
| 322A_01380 | 18272.97043 | 0.734571094 | 0.196923402 | 3.730237683 | 0.000191299 | 0.000277982 | Upregulated |

| GeneID | Base mean | log2(FC) | StdErr | Wald-Stats | P-value | P-adj_BH | Effect of menadione on gene expression |
|---|---|---|---|---|---|---|---|
| 322A_01476 | 286.2977788 | -0.403994395 | 0.108380143 | -3.727568374 | 0.000193336 | 0.000280684 | Downregulated |
| 322A_00161 | 377.6374418 | -1.009776784 | 0.271539847 | -3.718705723 | 0.000200246 | 0.000290449 | Downregulated |
| 322A_00811 | 13149.00414 | 0.650235632 | 0.174883562 | 3.718106053 | 0.000200722 | 0.000290872 | Upregulated |
| 322A_00480 | 3594.18845 | 0.508264663 | 0.136851852 | 3.71397723 | 0.000204027 | 0.000295391 | Upregulated |
| 322A_01353 | 505.8262665 | 0.618430048 | 0.167471823 | 3.692740898 | 0.00022185 | 0.000320901 | Upregulated |
| 322A_01423 | 2211.052312 | -0.526163236 | 0.142565734 | -3.690671115 | 0.000223663 | 0.000323228 | Downregulated |
| 322A_00862 | 7433.573966 | 0.952640358 | 0.258228722 | 3.689134002 | 0.000225019 | 0.00032489 | Upregulated |
| 322A_00153 | 2732.225026 | -1.031666229 | 0.280037999 | -3.684022282 | 0.000229582 | 0.000331176 | Downregulated |
| 322A_00147 | 8923.17926 | -0.302439383 | 0.082344435 | -3.67285761 | 0.000239853 | 0.000345677 | Downregulated |
| 322A_01589 | 18138.57759 | -0.858382618 | 0.235387639 | -3.646676704 | 0.000265654 | 0.000382512 | Downregulated |
| 322A_01291 | 6864.41727 | -0.510270403 | 0.139969048 | -3.645594576 | 0.000266774 | 0.000383776 | Downregulated |
| 322A_00970 | 1002.745736 | -0.618051037 | 0.169959724 | -3.63645587 | 0.000276415 | 0.000397284 | Downregulated |
| 322A_00013 | 307.0220152 | -1.114454594 | 0.306886968 | -3.631482306 | 0.000281798 | 0.000404653 | Downregulated |
| 322A_00781 | 518.1255904 | -0.388720477 | 0.10797798 | -3.599997664 | 0.00031822 | 0.000456539 | Downregulated |
| 322A_00560 | 102.6110787 | 0.753025284 | 0.209915458 | 3.587278865 | 0.000334147 | 0.000478954 | Upregulated |
| 322A_00449 | 932.0138983 | 0.260619054 | 0.07282311 | 3.578795992 | 0.000345181 | 0.000494321 | Upregulated |
| 322A_01324 | 5016.369838 | 0.659492264 | 0.18601408 | 3.545388947 | 0.000392034 | 0.00056091 | Upregulated |
| 322A_00262 | 3984.546516 | -0.281453226 | 0.079516198 | -3.539570972 | 0.000400778 | 0.000572902 | Downregulated |
| 322A_01568 | 158.5584344 | 1.931093602 | 0.54635641 | 3.534494271 | 0.000408556 | 0.000583494 | Upregulated |
| 322A_00698 | 1240.845176 | -0.248548905 | 0.070497677 | -3.525632557 | 0.000422473 | 0.000602824 | Downregulated |
| 322A_01512 | 251.4766237 | -1.037051474 | 0.295415855 | -3.510480081 | 0.000447298 | 0.000637672 | Downregulated |
| 322A_00589 | 21505.42227 | -0.748390078 | 0.213212729 | -3.510062849 | 0.000448001 | 0.000638098 | Downregulated |
| 322A_00533 | 1533.854485 | 0.588157651 | 0.167576434 | 3.509787363 | 0.000448465 | 0.000638185 | Upregulated |
| 322A_00388 | 7201.954415 | -0.427442164 | 0.121903847 | -3.506387802 | 0.000454233 | 0.000645811 | Downregulated |
| 322A_01413 | 1401.091079 | 0.441587643 | 0.126157221 | 3.500296216 | 0.000464741 | 0.000660158 | Upregulated |
| 322A_01564 | 1468.296851 | -1.139723807 | 0.326201622 | -3.493924408 | 0.000475976 | 0.00067551 | Downregulated |
| 322A_00217 | 27742.42288 | -0.531863233 | 0.15243509 | -3.489112862 | 0.000484626 | 0.00068717 | Downregulated |
| 322A_00122 | 4729.038607 | 0.510581832 | 0.146762853 | 3.478958211 | 0.000503367 | 0.000713103 | Upregulated |
| 322A_00903 | 938.6935659 | 1.243423455 | 0.357605864 | 3.477077919 | 0.00050691 | 0.00071748 | Upregulated |
| 322A_00297 | 854.2423881 | -0.99242491 | 0.287170822 | -3.455869584 | 0.000548521 | 0.000775681 | Downregulated |
| 322A_00609 | 2843.118332 | 1.065605616 | 0.30848911 | 3.454273044 | 0.000551778 | 0.00077959 | Upregulated |
| 322A_00184 | 9846.018078 | -0.282299447 | 0.081849845 | -3.448991822 | 0.000562684 | 0.000794288 | Downregulated |
| 322A_00470 | 7980.033778 | -1.372855323 | 0.401584316 | -3.418597957 | 0.000629447 | 0.000887739 | Downregulated |
| 322A_00593 | 1921.654166 | -0.757013091 | 0.221479083 | -3.41798909 | 0.000630856 | 0.000888934 | Downregulated |
| 322A_00710 | 6044.999337 | -0.80065742 | 0.234792886 | -3.410058253 | 0.00064949 | 0.000914376 | Downregulated |
| 322A_01261 | 22.74966994 | -1.189288648 | 0.349030241 | -3.407408612 | 0.000655829 | 0.000922478 | Downregulated |
| 322A_00100 | 214.8784044 | -0.869137998 | 0.256648437 | -3.386492467 | 0.000707922 | 0.000994867 | Downregulated |
| 322A_01431 | 907.1530829 | 0.634814587 | 0.187644936 | 3.38306271 | 0.000716823 | 0.00100648 | Upregulated |
| 322A_00814 | 2435.86549 | 0.694182161 | 0.205243332 | 3.382239775 | 0.000718974 | 0.001008604 | Upregulated |
| 322A_01590 | 1203.681595 | -1.319209355 | 0.391019513 | -3.373768598 | 0.000741467 | 0.001039237 | Downregulated |
| 322A_00894 | 4679.597176 | 0.633113103 | 0.187844942 | 3.370402723 | 0.000750584 | 0.001051084 | Upregulated |
| 322A_01404 | 18125.63224 | -0.59565397 | 0.177094341 | -3.36348392 | 0.000769653 | 0.001076833 | Downregulated |

| GeneID | Base mean | log2(FC) | StdErr | Wald-Stats | P-value | P-adj_BH | Effect of menadione on gene expression |
|---|---|---|---|---|---|---|---|
| 322A_00181 | 447.1093969 | -0.573363506 | 0.171008082 | -3.352844503 | 0.000799856 | 0.001118102 | Downregulated |
| 322A_01111 | 50.6788711 | -0.818033943 | 0.24408209 | -3.351470579 | 0.000803836 | 0.001122672 | Downregulated |
| 322A_00343 | 2751.180213 | -0.327300133 | 0.097949039 | -3.341534916 | 0.000833165 | 0.001162608 | Downregulated |
| 322A_00528 | 5067.353745 | -0.23021814 | 0.06902749 | -3.335166009 | 0.000852485 | 0.001188517 | Downregulated |
| 322A_00380 | 12918.98342 | 0.221596845 | 0.066651361 | 3.324715977 | 0.000885087 | 0.001232883 | Upregulated |
| 322A_00569 | 740.5103794 | 0.517414985 | 0.156823669 | 3.299342438 | 0.000969116 | 0.001348743 | Upregulated |
| 322A_00669 | 3051.558017 | 0.57980215 | 0.176191741 | 3.290745334 | 0.000999223 | 0.001389421 | Upregulated |
| 322A_00077 | 1537.808713 | -0.587419738 | 0.178625478 | -3.288555162 | 0.00100703 | 0.001399047 | Downregulated |
| 322A_00070 | 1754.766876 | 0.985818567 | 0.301189222 | 3.273087127 | 0.001063797 | 0.001476613 | Upregulated |
| 322A_01315 | 4771.071936 | -0.53000339 | 0.162019181 | -3.271238541 | 0.001070775 | 0.001484997 | Downregulated |
| 322A_00277 | 4245.127463 | -1.352093973 | 0.415010584 | -3.257974676 | 0.001122104 | 0.001554817 | Downregulated |
| 322A_00149 | 22689.48729 | 0.313747925 | 0.096858545 | 3.239238456 | 0.001198493 | 0.00165921 | Upregulated |
| 322A_00223 | 1642.691331 | 0.457845973 | 0.141768974 | 3.229521659 | 0.001239975 | 0.001715136 | Upregulated |
| 322A_00987 | 3334.829751 | -0.561603183 | 0.173955007 | -3.228439311 | 0.001244677 | 0.001720134 | Downregulated |
| 322A_01258 | 36.50405653 | -1.327468657 | 0.412918757 | -3.214842227 | 0.001305162 | 0.00180215 | Downregulated |
| 322A_00782 | 1930.545411 | -0.314917545 | 0.098043445 | -3.212020386 | 0.00131805 | 0.001818357 | Downregulated |
| 322A_01027 | 6374.308699 | 0.560626151 | 0.175176385 | 3.200352322 | 0.001372597 | 0.001891958 | Upregulated |
| 322A_00518 | 381.6146429 | 0.405231141 | 0.126748721 | 3.197122132 | 0.001388061 | 0.001911607 | Upregulated |
| 322A_00796 | 5793.300236 | 0.193991531 | 0.061044982 | 3.177845676 | 0.001483737 | 0.002041591 | Upregulated |
| 322A_00435 | 2314.023457 | 0.870791503 | 0.274479957 | 3.172513991 | 0.001511253 | 0.002075839 | Upregulated |
| 322A_01410 | 954.9765355 | 1.258308251 | 0.396601489 | 3.172726993 | 0.001510145 | 0.002075839 | Upregulated |
| 322A_01398 | 12526.72269 | 0.512362458 | 0.162447711 | 3.154014634 | 0.00161041 | 0.00221012 | Upregulated |
| 322A_00976 | 4367.409728 | -0.475218125 | 0.151182891 | -3.143332696 | 0.001670359 | 0.002290405 | Downregulated |
| 322A_01490 | 2125.798482 | 0.246575585 | 0.078499479 | 3.14111109 | 0.001683082 | 0.002305851 | Upregulated |
| 322A_00489 | 2534.071175 | -0.294200608 | 0.093691209 | -3.140108997 | 0.00168885 | 0.00231175 | Downregulated |
| 322A_01304 | 2568.573208 | -0.452920855 | 0.144598331 | -3.132268901 | 0.001734609 | 0.002372333 | Downregulated |
| 322A_00063 | 9862.541536 | -0.509555477 | 0.162918734 | -3.127666569 | 0.001761999 | 0.002407711 | Downregulated |
| 322A_00141 | 28760.3063 | -0.385826678 | 0.123456535 | -3.125202549 | 0.001776827 | 0.002425875 | Downregulated |
| 322A_00545 | 25939.54153 | 0.409659233 | 0.132380977 | 3.094547586 | 0.001971133 | 0.002688836 | Upregulated |
| 322A_00050 | 436.5781293 | -0.801346928 | 0.259051372 | -3.093390018 | 0.001978839 | 0.002697021 | Downregulated |
| 322A_00863 | 5854.186163 | 0.872057759 | 0.282587312 | 3.085976335 | 0.00202885 | 0.0027628 | Upregulated |
| 322A_00071 | 6075.950364 | -0.778792773 | 0.254074115 | -3.065218877 | 0.002175108 | 0.002959419 | Downregulated |
| 322A_01601 | 1679.801906 | 0.462105441 | 0.151481803 | 3.050567349 | 0.002284094 | 0.003105033 | Upregulated |
| 322A_00437 | 4804.462805 | 0.459986318 | 0.151357215 | 3.039077572 | 0.002373037 | 0.003223172 | Upregulated |
| 322A_00323 | 1117.721903 | -0.538989951 | 0.177950183 | -3.028881125 | 0.002454612 | 0.003331109 | Downregulated |
| 322A_01140 | 1565.613324 | -0.72275681 | 0.239364122 | -3.019486812 | 0.002532033 | 0.003433228 | Downregulated |
| 322A_00412 | 760.7288509 | 0.595384081 | 0.197665341 | 3.012081319 | 0.002594631 | 0.003515091 | Upregulated |
| 322A_00299 | 3134.528022 | -0.46035156 | 0.153748008 | -2.994195289 | 0.002751698 | 0.003724687 | Downregulated |
| 322A_01126 | 189.9686988 | -1.075885503 | 0.359470979 | -2.992969018 | 0.002762778 | 0.003736486 | Downregulated |
| 322A_01207 | 37976.58387 | -0.686735278 | 0.229845831 | -2.987808287 | 0.002809857 | 0.00379691 | Downregulated |
| 322A_01068 | 2419.595432 | -0.793576201 | 0.266984762 | -2.972365145 | 0.00295515 | 0.003989831 | Downregulated |
| 322A_00413 | 2550.087514 | 0.315211099 | 0.106116522 | 2.970424323 | 0.002973887 | 0.004011702 | Upregulated |

| GeneID | Base mean | log2(FC) | StdErr | Wald-Stats | P-value | P-adj_BH | Effect of menadione on gene expression |
|---|---|---|---|---|---|---|---|
| 322A_01390 | 1668.709543 | 0.665835203 | 0.22436718 | 2.967614084 | 0.003001209 | 0.004045108 | Upregulated |
| 322A_00720 | 614.4920374 | -1.008863952 | 0.340976111 | -2.958752588 | 0.00308887 | 0.004159713 | Downregulated |
| 322A_00229 | 8301.635954 | -0.299382185 | 0.101439776 | -2.951329326 | 0.003164094 | 0.004257389 | Downregulated |
| 322A_01132 | 1223.1497 | 0.201916801 | 0.068430571 | 2.950681233 | 0.00317074 | 0.004262704 | Upregulated |
| 322A_00571 | 2557.253044 | -0.330024139 | 0.11218127 | -2.941882718 | 0.003262235 | 0.004378262 | Downregulated |
| 322A_01411 | 639.2223189 | -0.807269461 | 0.274385792 | -2.94209644 | 0.003259984 | 0.004378262 | Downregulated |
| 322A_01414 | 1125.414484 | 0.597343569 | 0.204555322 | 2.920205458 | 0.003498007 | 0.004690711 | Upregulated |
| 322A_01221 | 4563.522573 | -0.374721613 | 0.128628475 | -2.913208861 | 0.003577352 | 0.004793046 | Downregulated |
| 322A_00646 | 4983.892745 | 0.158538324 | 0.054639564 | 2.901529791 | 0.003713455 | 0.004971187 | Upregulated |
| 322A_01212 | 2345.240401 | 0.730119754 | 0.252430128 | 2.892363765 | 0.00382355 | 0.00511424 | Upregulated |
| 322A_00474 | 9104.379173 | -0.680515095 | 0.235462304 | -2.89012331 | 0.003850907 | 0.005146479 | Downregulated |
| 322A_00870 | 2179.588998 | 0.423811895 | 0.147122062 | 2.880682132 | 0.003968156 | 0.005298695 | Upregulated |
| 322A_00074 | 10590.17874 | 0.219536432 | 0.07626724 | 2.87851551 | 0.003995516 | 0.005330727 | Upregulated |
| 322A_00683 | 1489.006033 | -0.654672268 | 0.229421061 | -2.853583999 | 0.00432291 | 0.005762665 | Downregulated |
| 322A_00057 | 564.286752 | 0.837270341 | 0.294830711 | 2.839834219 | 0.004513698 | 0.00600726 | Upregulated |
| 322A_00106 | 1360.764437 | -0.643146648 | 0.226474974 | -2.839813317 | 0.004513994 | 0.00600726 | Downregulated |
| 322A_01267 | 49.95878705 | 0.957600106 | 0.338979549 | 2.824949495 | 0.004728807 | 0.006287842 | Upregulated |
| 322A_01480 | 40.18456606 | 0.884495693 | 0.313452484 | 2.821785564 | 0.00477571 | 0.006344872 | Upregulated |
| 322A_01169 | 641.3672412 | 0.240370347 | 0.086336225 | 2.784119254 | 0.00536733 | 0.007124893 | Upregulated |
| 322A_00540 | 6260.733782 | -1.164018538 | 0.41895845 | -2.778362721 | 0.005463359 | 0.007246284 | Downregulated |
| 322A_01040 | 162.7952572 | -1.086117529 | 0.391992625 | -2.770760107 | 0.005592561 | 0.007411433 | Downregulated |
| 322A_00192 | 4628.518404 | -1.1058458 | 0.399989714 | -2.764685597 | 0.005697768 | 0.007544532 | Downregulated |
| 322A_00237 | 5766.405463 | 0.251581279 | 0.091645768 | 2.745148898 | 0.006048345 | 0.008002036 | Upregulated |
| 322A_01072 | 3205.339916 | -0.286998741 | 0.104770616 | -2.739305664 | 0.00615691 | 0.008138859 | Downregulated |
| 322A_01020 | 5529.147443 | 0.669174732 | 0.244653595 | 2.735192717 | 0.006234376 | 0.008234377 | Upregulated |
| 322A_01107 | 114.2155562 | 1.658180056 | 0.610024481 | 2.718218872 | 0.006563441 | 0.008661769 | Upregulated |
| 322A_00202 | 928.0488819 | -0.911206286 | 0.335470945 | -2.716200312 | 0.006603595 | 0.008703714 | Downregulated |
| 322A_01323 | 33200.96869 | -0.341142246 | 0.125601511 | -2.716068 | 0.006606235 | 0.008703714 | Downregulated |
| 322A_01362 | 1830.893605 | -1.007007415 | 0.373371248 | -2.697067381 | 0.006995311 | 0.009208649 | Downregulated |
| 322A_01466 | 749.2887864 | -0.412423028 | 0.15294604 | -2.696526357 | 0.007006685 | 0.009215948 | Downregulated |
| 322A_00055 | 87.7327116 | 1.351838725 | 0.502021385 | 2.692791117 | 0.007085665 | 0.009312083 | Upregulated |
| 322A_00407 | 6720.39024 | -1.059841102 | 0.393973004 | -2.690136355 | 0.007142283 | 0.009378695 | Downregulated |
| 322A_01544 | 4581.497799 | -0.60244502 | 0.224122302 | -2.688019062 | 0.007187729 | 0.009430539 | Downregulated |
| 322A_00142 | 17843.67149 | -0.718756927 | 0.267973548 | -2.682193565 | 0.007314113 | 0.009588401 | Downregulated |
| 322A_00311 | 1441.722464 | -0.417158488 | 0.155952189 | -2.674912679 | 0.007474872 | 0.009791029 | Downregulated |
| 322A_01447 | 473.1341484 | 0.485116773 | 0.181511428 | 2.672651412 | 0.00752544 | 0.009849107 | Upregulated |
| 322A_01119 | 837.9391591 | -0.567714894 | 0.213851569 | -2.654714657 | 0.007937552 | 0.010379876 | Downregulated |
| 322A_00654 | 157.40954 | 0.838778249 | 0.316905114 | 2.646780414 | 0.00812621 | 0.0106178 | Upregulated |
| 322A_01160 | 3119.405065 | 0.173536213 | 0.065822072 | 2.636444086 | 0.008378001 | 0.010937753 | Upregulated |
| 322A_01250 | 290.1033755 | 0.380138751 | 0.144240604 | 2.635448961 | 0.008402606 | 0.010960825 | Upregulated |
| 322A_00691 | 827.1540313 | 0.362891565 | 0.13827935 | 2.624336649 | 0.008681793 | 0.011315675 | Upregulated |
| 322A_01073 | 4638.491306 | -0.234496618 | 0.08949818 | -2.62012724 | 0.008789696 | 0.011446878 | Downregulated |

| GeneID | Base mean | log2(FC) | StdErr | Wald-Stats | P-value | P-adj_BH | Effect of menadione on gene expression |
|---|---|---|---|---|---|---|---|
| 322A_01262 | 23.13494871 | -1.569690539 | 0.599768199 | -2.617161998 | 0.008866425 | 0.011537299 | Downregulated |
| 322A_01084 | 3040.863014 | -0.335937968 | 0.128829514 | -2.607616508 | 0.009117504 | 0.011854254 | Downregulated |
| 322A_01195 | 22393.21908 | -0.540476775 | 0.210151426 | -2.571844427 | 0.010115834 | 0.013141441 | Downregulated |
| 322A_00864 | 1064.156218 | -0.992687615 | 0.386222751 | -2.570246349 | 0.010162622 | 0.013191384 | Downregulated |
| 322A_00178 | 303.8292486 | -0.425943434 | 0.166140157 | -2.563759674 | 0.010354521 | 0.013428155 | Downregulated |
| 322A_01585 | 108.2574151 | -1.175279118 | 0.458465082 | -2.563508462 | 0.010362017 | 0.013428155 | Downregulated |
| 322A_00497 | 1815.634496 | 0.38807206 | 0.151464658 | 2.562129443 | 0.010403254 | 0.013470552 | Upregulated |
| 322A_01343 | 333.6726483 | 0.878936691 | 0.344947234 | 2.54803229 | 0.010833244 | 0.014015842 | Upregulated |
| 322A_00620 | 6118.191503 | -0.523216763 | 0.205476083 | -2.546363335 | 0.010885182 | 0.014071524 | Downregulated |
| 322A_00056 | 74.66446617 | 1.682831596 | 0.663691389 | 2.535563401 | 0.01122666 | 0.014501103 | Upregulated |
| 322A_00422 | 5096.005386 | 0.4586651 | 0.181919067 | 2.521259091 | 0.011693572 | 0.015091867 | Upregulated |
| 322A_01567 | 7217.353612 | -0.45153321 | 0.180200615 | -2.505725138 | 0.012220057 | 0.015758492 | Downregulated |
| 322A_01141 | 245.5972886 | -0.896096473 | 0.363701031 | -2.463827143 | 0.013746239 | 0.017712146 | Downregulated |
| 322A_01329 | 254.719329 | -0.433025966 | 0.176650776 | -2.451310854 | 0.014233697 | 0.018325306 | Downregulated |
| 322A_00325 | 2271.873653 | 0.3814049 | 0.156409246 | 2.438506099 | 0.01474811 | 0.018972141 | Upregulated |
| 322A_00130 | 140.9880365 | 1.753994286 | 0.723219022 | 2.425260166 | 0.01529742 | 0.019662781 | Upregulated |
| 322A_00132 | 13341.46843 | 0.61979721 | 0.257269297 | 2.409137883 | 0.015990256 | 0.020536632 | Upregulated |
| 322A_01570 | 88.39869587 | 0.592313453 | 0.246046195 | 2.40732621 | 0.016069809 | 0.020622051 | Upregulated |
| 322A_00931 | 685.3712626 | -0.641457168 | 0.266771436 | -2.404519676 | 0.016193734 | 0.020764229 | Downregulated |
| 322A_01191 | 586.9116339 | 0.446369866 | 0.186036769 | 2.39936367 | 0.016423594 | 0.021041898 | Upregulated |
| 322A_00722 | 14899.27518 | 0.317583223 | 0.132575194 | 2.395495073 | 0.016597937 | 0.021248048 | Upregulated |
| 322A_00929 | 3.987724911 | 2.237683256 | 0.935004912 | 2.393231553 | 0.016700698 | 0.0213623 | Upregulated |
| 322A_00069 | 29.16601936 | 0.730913902 | 0.306177185 | 2.387225232 | 0.016976087 | 0.021697004 | Upregulated |
| 322A_00667 | 2575.307147 | 0.24096192 | 0.101000777 | 2.385743221 | 0.017044647 | 0.021767034 | Upregulated |
| 322A_00220 | 26.52722671 | 1.150180658 | 0.485318736 | 2.369949012 | 0.017790539 | 0.022701244 | Upregulated |
| 322A_00788 | 2578.797763 | -0.340158445 | 0.144527388 | -2.353591592 | 0.018593027 | 0.023706109 | Downregulated |
| 322A_00789 | 26481.00974 | -0.446915715 | 0.190218182 | -2.349489989 | 0.01879915 | 0.023949602 | Downregulated |
| 322A_00037 | 34446.04031 | -0.461453508 | 0.197372508 | -2.337982688 | 0.019388146 | 0.02468008 | Downregulated |
| 322A_01527 | 44.0607588 | 0.621224911 | 0.265795471 | 2.337229106 | 0.019427275 | 0.024709993 | Upregulated |
| 322A_01085 | 9777.382094 | -0.359870076 | 0.154007147 | -2.336710227 | 0.019454256 | 0.024724421 | Downregulated |
| 322A_00883 | 11604.58924 | -0.581305798 | 0.249393206 | -2.330880648 | 0.019759654 | 0.02509238 | Downregulated |
| 322A_01575 | 590.150748 | 1.180535135 | 0.507560033 | 2.325902471 | 0.020023754 | 0.025407348 | Upregulated |
| 322A_00834 | 1522.6746 | 0.421469099 | 0.181585427 | 2.32105135 | 0.020284073 | 0.025704609 | Upregulated |
| 322A_01144 | 6238.97746 | -0.341951758 | 0.147333848 | -2.32093142 | 0.020290545 | 0.025704609 | Downregulated |
| 322A_01467 | 337.0905282 | -0.791665211 | 0.341973182 | -2.314992087 | 0.020613365 | 0.026092658 | Downregulated |
| 322A_00975 | 2616.763429 | 0.41139944 | 0.178461164 | 2.305260322 | 0.021151998 | 0.026753047 | Upregulated |
| 322A_01298 | 6844.278262 | -0.459062869 | 0.200150269 | -2.293591068 | 0.021813999 | 0.027568291 | Downregulated |
| 322A_01039 | 711.0903809 | -0.329521389 | 0.143725517 | -2.292713196 | 0.021864522 | 0.027610072 | Downregulated |
| 322A_00828 | 3082.491317 | 0.210882813 | 0.092152143 | 2.28842006 | 0.022113071 | 0.027879879 | Upregulated |
| 322A_01218 | 2985.12153 | 0.217062134 | 0.094852671 | 2.288413499 | 0.022113452 | 0.027879879 | Upregulated |
| 322A_01392 | 25.60283326 | -1.189084594 | 0.520519198 | -2.284420244 | 0.022346845 | 0.028151683 | Downregulated |
| 322A_01484 | 6063.072937 | -0.622711344 | 0.272903646 | -2.281799283 | 0.022501193 | 0.028323556 | Downregulated |

| GeneID | Base mean | log2(FC) | StdErr | Wald-Stats | P-value | P-adj_BH | Effect of menadione on gene expression |
|---|---|---|---|---|---|---|---|
| 322A_00105 | 4071.387913 | -0.3549205 | 0.157331436 | -2.25587784 | 0.024078283 | 0.030284618 | Downregulated |
| 322A_00812 | 9110.345419 | 0.439387291 | 0.19482302 | 2.255315059 | 0.02411356 | 0.03030488 | Upregulated |
| 322A_00997 | 2012.750622 | 0.304516074 | 0.135123569 | 2.253611837 | 0.024220598 | 0.030415223 | Upregulated |
| 322A_00544 | 2164.111407 | -0.513019867 | 0.22806853 | -2.249411027 | 0.024486358 | 0.030724549 | Downregulated |
| 322A_00805 | 6469.464759 | -0.64982087 | 0.28920432 | -2.246926565 | 0.024644721 | 0.030898734 | Downregulated |
| 322A_00774 | 15921.06774 | -0.176911292 | 0.078829132 | -2.244237476 | 0.024817126 | 0.031090235 | Downregulated |
| 322A_01546 | 3665.924445 | -0.392773841 | 0.176454676 | -2.225919147 | 0.026019598 | 0.03257085 | Downregulated |
| 322A_00656 | 1856.936257 | -0.582765212 | 0.261924416 | -2.224936569 | 0.026085497 | 0.032627508 | Downregulated |
| 322A_00752 | 1871.128509 | 0.791240459 | 0.356211116 | 2.221268296 | 0.026332795 | 0.032910789 | Upregulated |
| 322A_01215 | 2271.941346 | 0.195000028 | 0.087933486 | 2.217585554 | 0.026583104 | 0.033197383 | Upregulated |
| 322A_00190 | 3005.646767 | -0.175195301 | 0.079545986 | -2.202440504 | 0.027634207 | 0.03448278 | Downregulated |
| 322A_00046 | 5.063068437 | -1.547210155 | 0.702699863 | -2.201807964 | 0.027678876 | 0.03451128 | Downregulated |
| 322A_01473 | 34.50371213 | -0.641486148 | 0.291709749 | -2.199056255 | 0.027873922 | 0.034727085 | Downregulated |
| 322A_00502 | 104287.4876 | -0.598932333 | 0.273026611 | -2.193677502 | 0.028258602 | 0.035178622 | Downregulated |
| 322A_00888 | 395.9095958 | 0.301665323 | 0.138924447 | 2.171434404 | 0.029898351 | 0.037190631 | Upregulated |
| 322A_01370 | 7003.557451 | -0.419527101 | 0.19349226 | -2.16818544 | 0.03014458 | 0.037467438 | Downregulated |
| 322A_01358 | 8151.758826 | -0.495706682 | 0.228778779 | -2.166751148 | 0.030253834 | 0.037573694 | Downregulated |
| 322A_00360 | 6597.298281 | -0.779142791 | 0.360119052 | -2.163570038 | 0.030497363 | 0.037846414 | Downregulated |
| 322A_00367 | 124.3167364 | 0.623127464 | 0.288903019 | 2.156874189 | 0.031015469 | 0.038459181 | Upregulated |
| 322A_01167 | 484.9181042 | 0.201729335 | 0.093556015 | 2.156241199 | 0.031064836 | 0.038490208 | Upregulated |
| 322A_00626 | 531.7720152 | 0.693821858 | 0.322687849 | 2.150133205 | 0.03154468 | 0.039054142 | Upregulated |
| 322A_01248 | 10805.14455 | -0.229190546 | 0.106719838 | -2.147590833 | 0.031746274 | 0.039242267 | Downregulated |
| 322A_01327 | 11235.87055 | 0.259785936 | 0.120959451 | 2.147710937 | 0.031736726 | 0.039242267 | Upregulated |
| 322A_01440 | 810.2211877 | 0.47921724 | 0.223539631 | 2.143768591 | 0.032051433 | 0.039588528 | Upregulated |
| 322A_00021 | 7538.733449 | -0.356642271 | 0.166943995 | -2.136298893 | 0.032655059 | 0.040302614 | Downregulated |
| 322A_00845 | 4581.866126 | 0.250956135 | 0.118465326 | 2.118393151 | 0.034141785 | 0.042104651 | Upregulated |
| 322A_01612 | 1029.168827 | -0.488236829 | 0.230552098 | -2.117685471 | 0.034201714 | 0.042145682 | Downregulated |
| 322A_00357 | 2291.389573 | 0.389725792 | 0.184722012 | 2.109796168 | 0.034875917 | 0.04294301 | Upregulated |
| 322A_00280 | 4592.153907 | -0.525962906 | 0.250744648 | -2.09760372 | 0.035940166 | 0.044218991 | Downregulated |
| 322A_00186 | 1135.461488 | -0.319111617 | 0.152800918 | -2.088414262 | 0.036760482 | 0.045193097 | Downregulated |
| 322A_01399 | 6167.367098 | 0.325549365 | 0.156706374 | 2.077448142 | 0.037760217 | 0.046386094 | Upregulated |
| 322A_00948 | 4046.224613 | -0.306980429 | 0.14795711 | -2.074793359 | 0.03800569 | 0.046651395 | Downregulated |
| 322A_01356 | 4780.236286 | -0.191547519 | 0.093103705 | -2.057356568 | 0.03965194 | 0.048634381 | Downregulated |
| 322A_00064 | 5366.719497 | 0.353416194 | 0.171931651 | 2.055562147 | 0.03982474 | 0.04880846 | Upregulated |
| 322A_01432 | 3656.214237 | 0.486372228 | 0.237619391 | 2.046854113 | 0.040672411 | 0.049808738 | Upregulated |
| 322A_01043 | 6656.745609 | 0.314442576 | 0.15402943 | 2.041444777 | 0.041206638 | 0.050423912 | |
| 322A_01196 | 8076.586688 | -0.107277584 | 0.053076238 | -2.021197964 | 0.043259276 | 0.052894753 | |
| 322A_00685 | 512.355614 | 0.998395683 | 0.495334214 | 2.015600085 | 0.043841811 | 0.053565613 | |
| 322A_00275 | 3001.611312 | -0.318685748 | 0.158500263 | -2.010632294 | 0.04436431 | 0.054162143 | |
| 322A_01125 | 50.58091629 | -0.506241862 | 0.252190315 | -2.007380269 | 0.044709188 | 0.05454107 | |
| 322A_01130 | 5765.827817 | 0.465009397 | 0.231981443 | 2.00451118 | 0.04501533 | 0.054872195 | |
| 322A_01442 | 1538.573648 | 0.216309579 | 0.108435513 | 1.994822289 | 0.046062266 | 0.056105117 | |

| GeneID | Base mean | log2(FC) | StdErr | Wald-Stats | P-value | P-adj_BH | Effect of menadione on gene expression |
|---|---|---|---|---|---|---|---|
| 322A_00347 | 7260.808023 | -0.387296051 | 0.194358882 | -1.99268511 | 0.046295939 | 0.056346327 | |
| 322A_00200 | 31.96855834 | 0.684639674 | 0.344663281 | 1.986401544 | 0.046988755 | 0.057145556 | |
| 322A_00636 | 1692.290041 | -0.540564229 | 0.272488066 | -1.98380882 | 0.047277157 | 0.057452102 | |
| 322A_00442 | 457.0264956 | -0.680138946 | 0.342944405 | -1.98323383 | 0.047341317 | 0.057485885 | |
| 322A_01372 | 6701.276518 | -0.357898199 | 0.18104027 | -1.976898283 | 0.048053129 | 0.058305447 | |
| 322A_01481 | 2.433946604 | 1.838018557 | 0.937407823 | 1.960745912 | 0.04990867 | 0.060510436 | |
| 322A_01316 | 4954.726088 | -0.342029321 | 0.17466471 | -1.958205079 | 0.050205953 | 0.060824224 | |
| 322A_00431 | 1032.096004 | -0.32475105 | 0.167100827 | -1.943443704 | 0.051962571 | 0.062904154 | |
| 322A_01562 | 88447.01284 | -0.490124418 | 0.253379515 | -1.934349027 | 0.053070229 | 0.064195893 | |
| 322A_01453 | 10032.12069 | -0.195544772 | 0.101465045 | -1.927213175 | 0.053953069 | 0.065213916 | |
| 322A_01614 | 5744.15505 | 0.139648213 | 0.072509203 | 1.925937764 | 0.054112145 | 0.065356228 | |
| 322A_00675 | 1248.196351 | -0.72670456 | 0.380073685 | -1.912009664 | 0.055874954 | 0.067433819 | |
| 322A_01055 | 8255.916007 | -0.786909615 | 0.411961773 | -1.910152027 | 0.056113642 | 0.067670227 | |
| 322A_00247 | 480.9709292 | -0.239584748 | 0.125707828 | -1.905885671 | 0.05666504 | 0.068283101 | |
| 322A_00089 | 197.366246 | 0.611626015 | 0.322584158 | 1.896020001 | 0.0579574 | 0.069787242 | |
| 322A_00892 | 1702.101742 | 0.188831278 | 0.099982153 | 1.888649851 | 0.058938762 | 0.070914904 | |
| 322A_00376 | 950.5531568 | 0.579127389 | 0.306844292 | 1.887365691 | 0.059111156 | 0.071068242 | |
| 322A_01446 | 3.221285383 | 1.700184962 | 0.904551836 | 1.879588205 | 0.060164222 | 0.072279358 | |
| 322A_00248 | 670.3012911 | 0.357254061 | 0.190210267 | 1.87820598 | 0.060352992 | 0.072408019 | |
| 322A_00983 | 355.2540231 | -0.510011955 | 0.271552568 | -1.878133422 | 0.060362915 | 0.072408019 | |
| 322A_00250 | 344.9521942 | 0.171688093 | 0.091581808 | 1.874696482 | 0.060834491 | 0.07291837 | |
| 322A_00065 | 5385.698184 | -0.240991799 | 0.12899669 | -1.868201422 | 0.061734001 | 0.073940496 | |
| 322A_00466 | 4047.090014 | -0.259076511 | 0.139396528 | -1.858557843 | 0.063089837 | 0.075507216 | |
| 322A_01355 | 2003.131082 | 0.263215381 | 0.142661102 | 1.845039594 | 0.065031779 | 0.077772498 | |
| 322A_00889 | 3847.616209 | -0.410676501 | 0.22433428 | -1.830645326 | 0.067153499 | 0.080249192 | |
| 322A_01201 | 702.6315337 | -0.402335385 | 0.220195429 | -1.827174103 | 0.067673596 | 0.080809634 | |
| 322A_00807 | 15715.00924 | 0.204892884 | 0.112627116 | 1.819214516 | 0.068878709 | 0.082186595 | |
| 322A_01026 | 5617.015582 | 0.539892033 | 0.297993323 | 1.811758824 | 0.07002347 | 0.083489522 | |
| 322A_01401 | 4253.053852 | -0.150547293 | 0.083244103 | -1.808503989 | 0.070528097 | 0.084027823 | |
| 322A_01566 | 1125.654868 | 0.51391533 | 0.284503077 | 1.806361235 | 0.070861933 | 0.084361985 | |
| 322A_01478 | 1.75476822 | -1.702693285 | 0.944529013 | -1.802690295 | 0.071436867 | 0.084982458 | |
| 322A_00614 | 2802.642011 | -0.689990043 | 0.386498925 | -1.78523147 | 0.074223779 | 0.088231425 | |
| 322A_01468 | 112.0732791 | 0.303728252 | 0.170902324 | 1.777203752 | 0.075534722 | 0.089722311 | |
| 322A_01065 | 6214.987903 | 0.381361746 | 0.215380676 | 1.770640491 | 0.076620504 | 0.090943706 | |
| 322A_00806 | 8121.661682 | 0.245877404 | 0.139068179 | 1.768034967 | 0.077055058 | 0.091390883 | |
| 322A_01149 | 1547.678913 | -0.343161365 | 0.194876553 | -1.760916637 | 0.078252514 | 0.092741547 | |
| 322A_00962 | 6890.062391 | -0.402568867 | 0.228785465 | -1.759591091 | 0.078477163 | 0.092938124 | |
| 322A_01325 | 3841.571741 | 0.259219564 | 0.147421924 | 1.758351526 | 0.078687716 | 0.093117723 | |
| 322A_01165 | 4844.244204 | 0.388191783 | 0.221415358 | 1.753228799 | 0.079562742 | 0.094082793 | |
| 322A_01461 | 21.91354607 | 0.607892208 | 0.347394071 | 1.74986351 | 0.080141869 | 0.094696782 | |
| 322A_01000 | 280.8324151 | -0.498850118 | 0.287479252 | -1.735256076 | 0.082695458 | 0.097568298 | |
| 322A_01274 | 299.7638836 | 0.641931438 | 0.369920566 | 1.735322382 | 0.08268372 | 0.097568298 | |

| GeneID | Base mean | log2(FC) | StdErr | Wald-Stats | P-value | P-adj_BH | Effect of menadione on gene expression |
|---|---|---|---|---|---|---|---|
| 322A_00464 | 543.1334901 | -0.671100712 | 0.386966357 | -1.734261129 | 0.082871761 | 0.097703396 | |
| 322A_01347 | 55171.87245 | 0.380741095 | 0.220943253 | 1.723252866 | 0.084842807 | 0.099952667 | |
| 322A_01475 | 139.4929165 | 0.406384205 | 0.236038976 | 1.721682632 | 0.085127025 | 0.100212827 | |
| 322A_00642 | 746.5559022 | -0.433776821 | 0.252281191 | -1.719418001 | 0.085538287 | 0.100622047 | |
| 322A_00896 | 2820.617526 | 0.524099926 | 0.307081485 | 1.706712879 | 0.087875432 | 0.103294467 | |
| 322A_00553 | 8722.572305 | 0.359344654 | 0.21306577 | 1.686543331 | 0.091691196 | 0.107699689 | |
| 322A_00872 | 3071.453223 | -0.326886893 | 0.194518362 | -1.680493759 | 0.092861288 | 0.108993093 | |
| 322A_00904 | 4198.05403 | -0.497496012 | 0.296203466 | -1.679575254 | 0.093039986 | 0.109121823 | |
| 322A_00024 | 2988.931155 | 0.173761127 | 0.103827601 | 1.673554285 | 0.094218229 | 0.110421809 | |
| 322A_01024 | 25915.64365 | -0.35876072 | 0.216048369 | -1.660557413 | 0.096802368 | 0.113366329 | |
| 322A_00730 | 369.4177478 | -0.378628084 | 0.228228291 | -1.658988386 | 0.097118133 | 0.113651938 | |
| 322A_00696 | 362.6164374 | -0.816376678 | 0.492606795 | -1.657258258 | 0.097467273 | 0.113976152 | |
| 322A_00679 | 2967.686057 | 0.323637994 | 0.19605206 | 1.65077579 | 0.098784365 | 0.115430955 | |
| 322A_00972 | 4.666268144 | -1.203623359 | 0.734265518 | -1.639220867 | 0.10116727 | 0.118128104 | |
| 322A_00160 | 2790.294458 | -0.233423455 | 0.142723732 | -1.63549153 | 0.101946049 | 0.118949596 | |
| 322A_01510 | 621.4910121 | -0.32441003 | 0.199697997 | -1.624503177 | 0.104268463 | 0.121569646 | |
| 322A_01572 | 5673.687163 | 0.368415936 | 0.230347136 | 1.599394472 | 0.10973298 | 0.127846604 | |
| 322A_01449 | 5649.864497 | -0.279459879 | 0.175831241 | -1.589364198 | 0.111978193 | 0.130366364 | |
| 322A_01266 | 154.5790622 | -0.217895896 | 0.137220774 | -1.587922077 | 0.112303961 | 0.13064942 | |
| 322A_01477 | 6.719592675 | 0.956200499 | 0.602841623 | 1.586155407 | 0.112704061 | 0.131018471 | |
| 322A_00148 | 2097.054714 | -0.172117605 | 0.108607791 | -1.584762967 | 0.113020199 | 0.131289445 | |
| 322A_00673 | 608.54608 | -0.162852075 | 0.103103003 | -1.57950855 | 0.114219457 | 0.132585141 | |
| 322A_00657 | 9424.721149 | -0.204859121 | 0.129826028 | -1.577951084 | 0.114576848 | 0.132902418 | |
| 322A_00399 | 55.49864945 | -0.324156099 | 0.206035811 | -1.573299797 | 0.115649416 | 0.134048187 | |
| 322A_00384 | 10341.89819 | -0.187116365 | 0.119856466 | -1.561170384 | 0.118483555 | 0.137232601 | |
| 322A_01302 | 7029.843788 | -0.213287014 | 0.136678014 | -1.560507119 | 0.118640089 | 0.137313309 | |
| 322A_00503 | 96.49376912 | 0.735814454 | 0.48386856 | 1.520690771 | 0.128337456 | 0.148428323 | |
| 322A_00974 | 2709.919466 | -0.141957542 | 0.094418322 | -1.503495711 | 0.132711262 | 0.153374639 | |
| 322A_00592 | 8605.247483 | 0.207138497 | 0.138647108 | 1.493997966 | 0.135176151 | 0.1561092 | |
| 322A_00538 | 1064.783078 | -0.200537621 | 0.135445023 | -1.480583159 | 0.138717686 | 0.160082235 | |
| 322A_01091 | 7918.35206 | -0.21403447 | 0.145234934 | -1.473712029 | 0.140559143 | 0.162088989 | |
| 322A_00185 | 139.1276796 | -0.444194124 | 0.302166635 | -1.470030349 | 0.141553535 | 0.163116719 | |
| 322A_00218 | 344.2802639 | 0.1763103 | 0.119982486 | 1.469466967 | 0.141706175 | 0.16317368 | |
| 322A_01047 | 5560.834638 | -0.322341926 | 0.222553276 | -1.448380954 | 0.147510538 | 0.169733742 | |
| 322A_00111 | 2131.503015 | 0.178156993 | 0.123153139 | 1.44662973 | 0.148000655 | 0.170173844 | |
| 322A_00763 | 8651.83169 | 0.185952759 | 0.130290069 | 1.427221278 | 0.153516123 | 0.176387348 | |
| 322A_00648 | 777.0618613 | 0.543173231 | 0.381109949 | 1.42524023 | 0.154087771 | 0.176915589 | |
| 322A_00846 | 6415.813872 | 0.28436301 | 0.200101396 | 1.421094585 | 0.155289264 | 0.178165694 | |
| 322A_01373 | 16579.93211 | -0.190604627 | 0.136540651 | -1.395955162 | 0.162727998 | 0.186564876 | |
| 322A_01434 | 548.5987361 | -0.633662371 | 0.456672101 | -1.387565321 | 0.165269448 | 0.189341302 | |
| 322A_00946 | 6698.097257 | -0.20687511 | 0.150854801 | -1.371352511 | 0.170265094 | 0.194923326 | |
| 322A_00324 | 547.8119385 | 0.137438792 | 0.10076175 | 1.36399766 | 0.172568302 | 0.197417139 | |

| GeneID | Base mean | log2(FC) | StdErr | Wald-Stats | P-value | P-adj_BH | Effect of menadione on gene expression |
|---|---|---|---|---|---|---|---|
| 322A_01525 | 68.6629975 | -0.525804397 | 0.386210732 | -1.361444294 | 0.173373326 | 0.19819467 | |
| 322A_01273 | 21.7773247 | 0.519809819 | 0.383452781 | 1.355603205 | 0.175225452 | 0.200167225 | |
| 322A_01121 | 524.3458606 | -0.418050884 | 0.310578013 | -1.346041467 | 0.178289144 | 0.203519954 | |
| 322A_01430 | 4235.375293 | 0.101389379 | 0.075796747 | 1.337648149 | 0.181011161 | 0.206478099 | |
| 322A_00780 | 1335.890894 | 0.522485914 | 0.393094539 | 1.329160957 | 0.183794873 | 0.209502303 | |
| 322A_01034 | 24.19686787 | -0.653638045 | 0.493354218 | -1.324885894 | 0.185209 | 0.210962125 | |
| 322A_01216 | 1583.246658 | -0.142543311 | 0.108668839 | -1.311722039 | 0.189613938 | 0.215824072 | |
| 322A_00853 | 865.1033795 | -0.345719552 | 0.263645993 | -1.31130213 | 0.189755709 | 0.215830055 | |
| 322A_01265 | 97.04076347 | 0.508240557 | 0.387881929 | 1.310297073 | 0.190095357 | 0.216060934 | |
| 322A_00822 | 1637.065808 | -0.288958672 | 0.221099161 | -1.306918896 | 0.191240258 | 0.217206069 | |
| 322A_01346 | 96875.33321 | 0.299734564 | 0.229461312 | 1.306253159 | 0.191466481 | 0.217306896 | |
| 322A_01334 | 6.020697827 | 0.902952838 | 0.700063728 | 1.2898152 | 0.197114829 | 0.223557062 | |
| 322A_01110 | 108.2073542 | 0.261616519 | 0.203052904 | 1.288415548 | 0.197601349 | 0.223948196 | |
| 322A_01469 | 6.480257024 | -1.109012497 | 0.872892689 | -1.270502675 | 0.203905631 | 0.230927509 | |
| 322A_00922 | 11172.75838 | -0.195471158 | 0.154834033 | -1.262456026 | 0.20678474 | 0.234020525 | |
| 322A_00718 | 2835.785282 | 0.291424519 | 0.23167224 | 1.257917304 | 0.208421665 | 0.235666367 | |
| 322A_00957 | 9997.694197 | 0.12132658 | 0.096474847 | 1.257598052 | 0.208537158 | 0.235666367 | |
| 322A_00020 | 1946.690339 | -0.384325521 | 0.305862806 | -1.256529116 | 0.208924196 | 0.235850873 | |
| 322A_01097 | 3085.045112 | 0.200030423 | 0.159218908 | 1.25632329 | 0.208998781 | 0.235850873 | |
| 322A_00450 | 1629.906943 | 0.211186017 | 0.170134232 | 1.241290571 | 0.214498427 | 0.24188446 | |
| 322A_00251 | 3374.082164 | -0.160602216 | 0.131789405 | -1.218627674 | 0.222985541 | 0.251275937 | |
| 322A_00849 | 5556.731159 | 0.282506773 | 0.233371114 | 1.210547308 | 0.226068949 | 0.254569095 | |
| 322A_00515 | 3698.651163 | -0.293100564 | 0.242237437 | -1.209972198 | 0.226289561 | 0.254636154 | |
| 322A_01057 | 5352.528017 | 0.213169315 | 0.178552398 | 1.193875395 | 0.232526714 | 0.261468517 | |
| 322A_01120 | 693.9385847 | -0.245617427 | 0.206432306 | -1.189820684 | 0.234116878 | 0.263069499 | |
| 322A_00487 | 2627.996468 | -0.23717538 | 0.200197838 | -1.184705003 | 0.236134104 | 0.26514774 | |
| 322A_00427 | 4577.469894 | 0.176918647 | 0.150301727 | 1.177089916 | 0.239159619 | 0.268354406 | |
| 322A_01338 | 282.2988948 | -0.202228753 | 0.175964738 | -1.149257261 | 0.250449916 | 0.280823629 | |
| 322A_01389 | 2090.891124 | 0.157478385 | 0.137454479 | 1.145676635 | 0.251928975 | 0.282281863 | |
| 322A_00865 | 1386.333725 | -0.264179957 | 0.231091025 | -1.143185708 | 0.252961495 | 0.283238048 | |
| 322A_01214 | 2661.581722 | 0.170343543 | 0.149601357 | 1.138649721 | 0.254849282 | 0.285149833 | |
| 322A_01211 | 5.297943339 | 0.927346923 | 0.817324002 | 1.134613593 | 0.256537255 | 0.286835503 | |
| 322A_01361 | 2786.354729 | -0.234998772 | 0.211760947 | -1.109736124 | 0.267112752 | 0.298448947 | |
| 322A_00977 | 3034.078265 | 0.27449304 | 0.249388614 | 1.100663881 | 0.271042972 | 0.302626369 | |
| 322A_00356 | 699.1936532 | 0.496042304 | 0.452579132 | 1.096034415 | 0.273063714 | 0.304667418 | |
| 322A_00693 | 17545.23017 | 0.132592372 | 0.121716273 | 1.08935616 | 0.275996856 | 0.30772287 | |
| 322A_00213 | 2994.899028 | -0.144532377 | 0.133304196 | -1.08422977 | 0.278262935 | 0.310030797 | |
| 322A_01032 | 20.16435172 | -0.530711497 | 0.490176133 | -1.082695508 | 0.278943598 | 0.310570302 | |
| 322A_01542 | 47.04048484 | 0.380045466 | 0.353439256 | 1.075278028 | 0.282250271 | 0.314030737 | |
| 322A_01287 | 237.9087247 | 0.266853435 | 0.251941608 | 1.059187629 | 0.289514338 | 0.321886194 | |
| 322A_00397 | 676.7788326 | 0.28377126 | 0.269630283 | 1.052445802 | 0.292595064 | 0.325082781 | |
| 322A_00088 | 2476.062523 | -0.220314756 | 0.209934319 | -1.049446119 | 0.293972841 | 0.326384173 | |

| GeneID | Base mean | log2(FC) | StdErr | Wald-Stats | P-value | P-adj_BH | Effect of menadione on gene expression |
|---|---|---|---|---|---|---|---|
| 322A_00647 | 46.125127 | 0.728065478 | 0.694374993 | 1.048519151 | 0.294399483 | 0.326628479 | |
| 322A_00729 | 174.055085 | 0.533987202 | 0.516757844 | 1.03334126 | 0.301444225 | 0.334209902 | |
| 322A_00810 | 34250.97747 | 0.199341324 | 0.197377691 | 1.009948605 | 0.312519914 | 0.34624666 | |
| 322A_01142 | 8565.266108 | -0.319991612 | 0.318385031 | -1.005046031 | 0.314874686 | 0.348611259 | |
| 322A_01479 | 5.252128738 | -0.586524723 | 0.587842054 | -0.997759038 | 0.318396217 | 0.352263415 | |
| 322A_00659 | 7504.088501 | 0.341614248 | 0.34382707 | 0.993564142 | 0.320435109 | 0.354271264 | |
| 322A_00835 | 2512.508382 | -0.18289342 | 0.184820873 | -0.989571233 | 0.322383737 | 0.356176581 | |
| 322A_01176 | 4949.434182 | -0.199085969 | 0.202955226 | -0.980935414 | 0.326624592 | 0.360609972 | |
| 322A_01263 | 189.2761772 | -0.397725513 | 0.407668154 | -0.975610947 | 0.329257298 | 0.363262936 | |
| 322A_01082 | 605.5425857 | -0.133915508 | 0.139607232 | -0.95923045 | 0.337442664 | 0.372034067 | |
| 322A_01433 | 8340.749826 | 0.068273748 | 0.072351542 | 0.943639153 | 0.345354077 | 0.380491147 | |
| 322A_00686 | 1050.965983 | 0.076589386 | 0.081328766 | 0.941725658 | 0.346333115 | 0.381304078 | |
| 322A_00225 | 3331.443517 | 0.128790348 | 0.137629628 | 0.935774879 | 0.349389108 | 0.38440096 | |
| 322A_01033 | 60.48980516 | 0.329147626 | 0.35751755 | 0.920647463 | 0.357234514 | 0.392759225 | |
| 322A_00446 | 626.4764208 | 0.083240975 | 0.090499051 | 0.919799421 | 0.357677586 | 0.392973081 | |
| 322A_01044 | 1717.753307 | -0.113603514 | 0.12403324 | -0.915911851 | 0.359713131 | 0.394935041 | |
| 322A_01418 | 1915.794058 | 0.184896706 | 0.203386794 | 0.909089043 | 0.363303126 | 0.398599751 | |
| 322A_01272 | 3.971013154 | 0.917599334 | 1.020131377 | 0.899491334 | 0.368391009 | 0.403901655 | |
| 322A_00410 | 1962.451952 | -0.18595487 | 0.208735167 | -0.890865075 | 0.373001559 | 0.408673226 | |
| 322A_01580 | 270.4181516 | 0.244371427 | 0.275759189 | 0.886176915 | 0.375522201 | 0.41115 | |
| 322A_00198 | 25.47025807 | -0.566829879 | 0.643626739 | -0.880681061 | 0.37849047 | 0.414113103 | |
| 322A_01179 | 3953.199196 | -0.160332472 | 0.182505037 | -0.878509846 | 0.379667097 | 0.415113195 | |
| 322A_00735 | 5670.466065 | -0.143309614 | 0.165941465 | -0.863615453 | 0.387799173 | 0.423711466 | |
| 322A_00058 | 1568.146917 | -0.110221966 | 0.127888862 | -0.861857432 | 0.388765976 | 0.424474453 | |
| 322A_01471 | 18.19986676 | 0.372165454 | 0.4326547 | 0.860190481 | 0.389684051 | 0.425183219 | |
| 322A_00402 | 178.3579988 | 0.245777462 | 0.293183193 | 0.838306791 | 0.401858423 | 0.438164253 | |
| 322A_00933 | 21088.79189 | -0.104943153 | 0.126591624 | -0.828989709 | 0.40711023 | 0.443584614 | |
| 322A_00423 | 6034.084445 | -0.088500738 | 0.107239365 | -0.825263546 | 0.409221983 | 0.445578481 | |
| 322A_00547 | 556.5224828 | -0.182293596 | 0.222289554 | -0.820072708 | 0.41217466 | 0.448484609 | |
| 322A_00954 | 5.958846328 | 0.485249272 | 0.594542243 | 0.816172908 | 0.414401249 | 0.450597232 | |
| 322A_01474 | 51.69156712 | 0.179329979 | 0.220867782 | 0.811933626 | 0.416829718 | 0.452926312 | |
| 322A_01312 | 15319.6426 | 0.11318667 | 0.141897121 | 0.797667135 | 0.42506368 | 0.461556098 | |
| 322A_01540 | 2384.610843 | -0.079944832 | 0.10058118 | -0.794828935 | 0.426713017 | 0.463029018 | |
| 322A_01470 | 13.23785601 | 0.51543311 | 0.653611873 | 0.788592025 | 0.430350494 | 0.466655782 | |
| 322A_00953 | 90.01783912 | 0.147067699 | 0.186689616 | 0.787765824 | 0.430833695 | 0.466859542 | |
| 322A_01337 | 155.7218056 | -0.165969892 | 0.211414726 | -0.785044138 | 0.43242769 | 0.468265875 | |
| 322A_00852 | 3558.291594 | 0.237111322 | 0.312583852 | 0.758552689 | 0.448120183 | 0.484926769 | |
| 322A_01259 | 7.873463823 | -0.470214494 | 0.621656909 | -0.756389074 | 0.449415957 | 0.485996325 | |
| 322A_00652 | 10243.54984 | -0.087293765 | 0.117395408 | -0.743587554 | 0.457126035 | 0.493996077 | |
| 322A_01114 | 5.800115005 | -0.58677092 | 0.799273979 | -0.734129891 | 0.462869577 | 0.499861203 | |
| 322A_01286 | 5146.92719 | -0.151221603 | 0.206951523 | -0.730710269 | 0.464956142 | 0.501771781 | |
| 322A_00097 | 1.139366606 | -0.807429419 | 1.109306344 | -0.727868747 | 0.466693936 | 0.503303624 | |

| GeneID | Base mean | log2(FC) | StdErr | Wald-Stats | P-value | P-adj_BH | Effect of menadione on gene expression |
|---|---|---|---|---|---|---|---|
| 322A_00219 | 266.1120444 | 0.188291387 | 0.263762983 | 0.713865853 | 0.475310134 | 0.5122463 | |
| 322A_00204 | 5620.218279 | -0.132933062 | 0.189347257 | -0.702059611 | 0.482641989 | 0.519793586 | |
| 322A_00712 | 3481.634813 | -0.290673345 | 0.417363272 | -0.696451663 | 0.486146019 | 0.523210929 | |
| 322A_00850 | 7687.932355 | 0.181438103 | 0.261454448 | 0.693956841 | 0.487709275 | 0.524536302 | |
| 322A_00355 | 1983.068511 | 0.066705724 | 0.098048753 | 0.680332202 | 0.496294139 | 0.533406549 | |
| 322A_01290 | 1432.678956 | 0.107634371 | 0.159690031 | 0.674020602 | 0.500298216 | 0.537344755 | |
| 322A_00733 | 3994.982629 | 0.069160015 | 0.103521739 | 0.668072386 | 0.504087388 | 0.541046952 | |
| 322A_01436 | 93.18217719 | 0.101656299 | 0.156285069 | 0.650454324 | 0.515398797 | 0.552812413 | |
| 322A_01122 | 3.726251091 | 0.488353058 | 0.757347138 | 0.644820629 | 0.519043432 | 0.55634418 | |
| 322A_01257 | 2305.583848 | 0.074422084 | 0.115658243 | 0.643465455 | 0.519922123 | 0.556908453 | |
| 322A_00471 | 6408.067446 | 0.129793246 | 0.202121171 | 0.642155622 | 0.520772144 | 0.557441273 | |
| 322A_00492 | 313.9143848 | 0.245958829 | 0.384625458 | 0.639476206 | 0.522513188 | 0.558926488 | |
| 322A_01472 | 190.220344 | 0.105051864 | 0.170575651 | 0.615866703 | 0.537982499 | 0.57508474 | |
| 322A_00199 | 45.08388917 | -0.308907347 | 0.502748388 | -0.614437269 | 0.538926414 | 0.575704501 | |
| 322A_00090 | 169.6288985 | -0.126881828 | 0.210802805 | -0.601898198 | 0.547241904 | 0.584192742 | |
| 322A_00016 | 3644.621305 | -0.07792732 | 0.133569188 | -0.58342288 | 0.559608659 | 0.59681893 | |
| 322A_00959 | 2229.34966 | -0.045833363 | 0.078602616 | -0.583102261 | 0.559824462 | 0.59681893 | |
| 322A_00926 | 250.6964117 | 0.096299878 | 0.165670667 | 0.581272955 | 0.561056505 | 0.597729336 | |
| 322A_00826 | 7337.381987 | 0.142848529 | 0.247740453 | 0.576605587 | 0.564205924 | 0.600679843 | |
| 322A_00616 | 2268.077791 | -0.078535379 | 0.136482546 | -0.575424343 | 0.565004345 | 0.60112508 | |
| 322A_00938 | 2206.512355 | 0.062414792 | 0.108662879 | 0.574389273 | 0.56570441 | 0.601465147 | |
| 322A_00010 | 753.3538592 | -0.221707493 | 0.387924458 | -0.571522339 | 0.567645622 | 0.603123474 | |
| 322A_01530 | 54.94874275 | -0.132161338 | 0.234110101 | -0.564526421 | 0.572395924 | 0.607762227 | |
| 322A_00337 | 4358.707333 | 0.105799606 | 0.191767401 | 0.551707981 | 0.581148441 | 0.616641399 | |
| 322A_01118 | 83.68296728 | -0.095173268 | 0.17316185 | -0.549620304 | 0.582579829 | 0.617745614 | |
| 322A_01437 | 36.69448255 | 0.280445663 | 0.514060827 | 0.54554957 | 0.585375598 | 0.620294115 | |
| 322A_01565 | 444.8912332 | -0.126258153 | 0.232221599 | -0.543696855 | 0.586650097 | 0.621228267 | |
| 322A_01388 | 506.4151957 | 0.250462289 | 0.462048282 | 0.542069517 | 0.587770617 | 0.621998223 | |
| 322A_01482 | 16.0320472 | -0.202236004 | 0.38201623 | -0.529391131 | 0.596534149 | 0.630849826 | |
| 322A_00855 | 2228.771777 | 0.061196593 | 0.116844578 | 0.523743541 | 0.600456927 | 0.634573798 | |
| 322A_00784 | 33.13133113 | -0.161588204 | 0.314497862 | -0.513797465 | 0.6073936 | 0.641475806 | |
| 322A_01019 | 6319.600945 | 0.1705296 | 0.334600429 | 0.509651468 | 0.610295659 | 0.644110439 | |
| 322A_00511 | 726.3639349 | 0.26959504 | 0.556156288 | 0.484746906 | 0.627855889 | 0.662201575 | |
| 322A_00579 | 52.79550143 | -0.300709978 | 0.625319681 | -0.480889994 | 0.630594684 | 0.663786772 | |
| 322A_00848 | 6406.582745 | 0.157601452 | 0.327492854 | 0.481236307 | 0.630348559 | 0.663786772 | |
| 322A_01189 | 2600.29406 | -0.110727781 | 0.230555123 | -0.480265976 | 0.631038279 | 0.663786772 | |
| 322A_01320 | 4844.020432 | 0.045869207 | 0.095451353 | 0.480550618 | 0.630835919 | 0.663786772 | |
| 322A_01183 | 5295.316912 | -0.217613527 | 0.455826066 | -0.477404747 | 0.633073935 | 0.6654853 | |
| 322A_00196 | 2947.992322 | -0.070957498 | 0.149671572 | -0.474088009 | 0.635437149 | 0.66752567 | |
| 322A_01578 | 11088.65908 | 0.059715339 | 0.130068107 | 0.459108235 | 0.646156443 | 0.678335549 | |
| 322A_00183 | 1571.820316 | -0.033624546 | 0.073848358 | -0.455318799 | 0.648879904 | 0.68074262 | |
| 322A_01270 | 93.07284755 | 0.092838927 | 0.204778177 | 0.453363381 | 0.650287101 | 0.681766516 | |

| GeneID | Base mean | log2(FC) | StdErr | Wald-Stats | P-value | P-adj_BH | Effect of menadione on gene expression |
|---|---|---|---|---|---|---|---|
| 322A_00049 | 18.77567293 | -0.177127227 | 0.391861453 | -0.452014929 | 0.651258228 | 0.682332179 | |
| 322A_00597 | 4263.669955 | -0.058441764 | 0.132680796 | -0.440468894 | 0.659597536 | 0.690611725 | |
| 322A_01200 | 656.9641552 | -0.177358251 | 0.407752702 | -0.434965239 | 0.663587659 | 0.694329642 | |
| 322A_01359 | 42549.80351 | -0.074602679 | 0.172692403 | -0.431997455 | 0.665743263 | 0.696124404 | |
| 322A_01460 | 93.95875552 | -0.097857995 | 0.227212563 | -0.43068919 | 0.66669438 | 0.696658172 | |
| 322A_00166 | 5394.775834 | 0.122615746 | 0.29425998 | 0.416691886 | 0.67690379 | 0.706859242 | |
| 322A_00017 | 16185.65914 | 0.055720703 | 0.134471827 | 0.414367115 | 0.678605269 | 0.708005824 | |
| 322A_00421 | 14868.84802 | -0.040712618 | 0.098347221 | -0.41396816 | 0.678897426 | 0.708005824 | |
| 322A_01054 | 803.3863076 | 0.03855329 | 0.09472426 | 0.40700545 | 0.684003986 | 0.712861108 | |
| 322A_01260 | 92.15629961 | 0.171552007 | 0.433622796 | 0.395624974 | 0.692381711 | 0.72111692 | |
| 322A_00296 | 523.773772 | 0.056583625 | 0.148611348 | 0.380749018 | 0.703389493 | 0.732099268 | |
| 322A_01067 | 330.0288682 | 0.066338841 | 0.175429374 | 0.378151273 | 0.705318221 | 0.733623755 | |
| 322A_01016 | 4219.024869 | -0.094039243 | 0.252790584 | -0.372004533 | 0.709889475 | 0.737893004 | |
| 322A_01596 | 2673.029176 | -0.072357871 | 0.199434086 | -0.362815965 | 0.716742365 | 0.744526727 | |
| 322A_00019 | 15872.14194 | 0.067268663 | 0.187140962 | 0.359454514 | 0.719255099 | 0.746646298 | |
| 322A_00444 | 566.6517622 | -0.081490356 | 0.230501997 | -0.353534274 | 0.723687938 | 0.750755007 | |
| 322A_00697 | 6369.88786 | -0.078885653 | 0.223536768 | -0.352897886 | 0.724164996 | 0.750757284 | |
| 322A_00239 | 4870.97605 | -0.080633651 | 0.232178979 | -0.34729092 | 0.728372771 | 0.754624739 | |
| 322A_00821 | 708.7162259 | -0.137328966 | 0.401799051 | -0.341785194 | 0.732512553 | 0.757920384 | |
| 322A_01336 | 21.17891112 | 0.112851593 | 0.330059078 | 0.341913312 | 0.732416131 | 0.757920384 | |
| 322A_00854 | 2258.395544 | -0.050648185 | 0.148822515 | -0.340326089 | 0.733610972 | 0.758560462 | |
| 322A_00624 | 583.5060898 | 0.169897198 | 0.51776013 | 0.32813882 | 0.742806703 | 0.767566927 | |
| 322A_00847 | 4623.393507 | 0.088537004 | 0.277933241 | 0.318554931 | 0.750064034 | 0.77455992 | |
| 322A_00288 | 5262.234036 | -0.114652768 | 0.364520095 | -0.31453072 | 0.753117988 | 0.777205966 | |
| 322A_01303 | 0.097726444 | -0.351245359 | 1.127791191 | -0.311445382 | 0.755462058 | 0.779116447 | |
| 322A_01517 | 23252.51857 | 0.05363763 | 0.181254936 | 0.295923693 | 0.767288357 | 0.790797192 | |
| 322A_00557 | 291.1097554 | -0.095133835 | 0.324231557 | -0.29341325 | 0.769206299 | 0.79174164 | |
| 322A_01545 | 1934.075026 | -0.041024342 | 0.139787484 | -0.293476501 | 0.769157958 | 0.79174164 | |
| 322A_01006 | 5922.657464 | -0.048538126 | 0.17014361 | -0.285277393 | 0.775431631 | 0.797630065 | |
| 322A_00365 | 666.9388224 | -0.109412903 | 0.396902457 | -0.275666982 | 0.782803858 | 0.804689791 | |
| 322A_01041 | 170.9147144 | 0.065741038 | 0.246588248 | 0.266602477 | 0.789775248 | 0.811328569 | |
| 322A_00137 | 6492.074758 | 0.035280586 | 0.133953077 | 0.263380186 | 0.792257561 | 0.812295203 | |
| 322A_00927 | 778.3676334 | 0.091925113 | 0.348880862 | 0.263485686 | 0.792176255 | 0.812295203 | |
| 322A_01383 | 3666.934927 | 0.144544048 | 0.546405127 | 0.264536406 | 0.791366616 | 0.812295203 | |
| 322A_00801 | 7363.735518 | -0.106851294 | 0.426465083 | -0.250551096 | 0.802161195 | 0.821916299 | |
| 322A_00582 | 17056.77456 | 0.052879778 | 0.213458846 | 0.247728211 | 0.804344703 | 0.823619803 | |
| 322A_00112 | 10644.31746 | 0.036083541 | 0.148913158 | 0.242312642 | 0.808537919 | 0.827377638 | |
| 322A_00721 | 23382.22197 | -0.012966431 | 0.053767765 | -0.241156216 | 0.809434044 | 0.827414627 | |
| 322A_00924 | 655.6607677 | 0.107553398 | 0.44643656 | 0.2409153 | 0.809620764 | 0.827414627 | |
| 322A_00681 | 393.7705215 | 0.072548715 | 0.309588515 | 0.234339166 | 0.814721682 | 0.83208978 | |
| 322A_01528 | 67.53173898 | 0.070513719 | 0.302639704 | 0.232995598 | 0.815764824 | 0.832617293 | |
| 322A_01428 | 751.0147473 | 0.121870095 | 0.540989758 | 0.225272463 | 0.82176732 | 0.838202667 | |

| GeneID | Base mean | log2(FC) | StdErr | Wald-Stats | P-value | P-adj_BH | Effect of menadione on gene expression |
|---|---|---|---|---|---|---|---|
| 322A_00221 | 164.2949797 | 0.119326796 | 0.53290018 | 0.223919601 | 0.822819864 | 0.838735141 | |
| 322A_01416 | 1661.688291 | -0.027100692 | 0.122474273 | -0.221276608 | 0.824877063 | 0.840290359 | |
| 322A_00327 | 1424.668867 | 0.025161365 | 0.116448635 | 0.216072654 | 0.828931116 | 0.843876429 | |
| 322A_00504 | 125.4928488 | -0.086662817 | 0.419960157 | -0.206359618 | 0.836510012 | 0.85104397 | |
| 322A_00110 | 260.2725465 | 0.071757759 | 0.356510193 | 0.201278281 | 0.840480984 | 0.854534043 | |
| 322A_01341 | 3660.204529 | 0.023319637 | 0.119712456 | 0.194797077 | 0.84555181 | 0.859137154 | |
| 322A_01162 | 4750.937926 | -0.042193743 | 0.227661424 | -0.185335495 | 0.852965899 | 0.866113736 | |
| 322A_00451 | 3425.567549 | -0.135680936 | 0.76002057 | -0.178522716 | 0.858312483 | 0.870983335 | |
| 322A_01415 | 1607.102694 | -0.058855297 | 0.383566569 | -0.153442196 | 0.878049571 | 0.890440264 | |
| 322A_00871 | 1657.942025 | -0.04075415 | 0.27135536 | -0.150187379 | 0.880616783 | 0.89247124 | |
| 322A_00928 | 0.404135601 | -0.170956166 | 1.164958914 | -0.146748665 | 0.883330405 | 0.894647899 | |
| 322A_00417 | 6.306708438 | -0.087656466 | 0.628015715 | -0.139576866 | 0.88899432 | 0.899807951 | |
| 322A_01151 | 12678.26658 | -0.018512354 | 0.134501683 | -0.137636601 | 0.890527628 | 0.900783224 | |
| 322A_01599 | 397.5303186 | -0.054205528 | 0.418756603 | -0.129443997 | 0.897006336 | 0.906756405 | |
| 322A_01175 | 422.8566167 | -0.019432408 | 0.152692472 | -0.127265005 | 0.898730657 | 0.907918957 | |
| 322A_00066 | 1170.72195 | -0.014845051 | 0.120140891 | -0.123563679 | 0.901660752 | 0.910297349 | |
| 322A_01387 | 9048.931159 | -0.017251818 | 0.144702175 | -0.119222935 | 0.905098737 | 0.913185132 | |
| 322A_00443 | 40.70289699 | -0.026455555 | 0.246643357 | -0.107262387 | 0.914580824 | 0.922163445 | |
| 322A_01226 | 442.9282163 | -0.083588093 | 0.791506142 | -0.105606373 | 0.915894668 | 0.922899599 | |
| 322A_00878 | 3144.583339 | -0.008178309 | 0.083412324 | -0.098046768 | 0.921895157 | 0.928354295 | |
| 322A_01524 | 161.1659133 | 0.042967888 | 0.482744434 | 0.089007526 | 0.929075929 | 0.934989843 | |
| 322A_00851 | 952.4058358 | 0.025628478 | 0.297825473 | 0.086052003 | 0.931425078 | 0.936757665 | |
| 322A_00201 | 3.278085922 | 0.06976613 | 0.908858774 | 0.076762344 | 0.938812607 | 0.943587242 | |
| 322A_00113 | 934.4201491 | 0.009419385 | 0.138736284 | 0.06789417 | 0.945869879 | 0.950076416 | |
| 322A_00550 | 1221.890936 | 0.04705399 | 0.737853783 | 0.063771429 | 0.949152228 | 0.952768046 | |
| 322A_00737 | 8198.238908 | 0.00755928 | 0.129254513 | 0.058483684 | 0.953363358 | 0.956387988 | |
| 322A_01462 | 19.67567566 | -0.033949117 | 0.60361581 | -0.056242921 | 0.955148289 | 0.957570986 | |
| 322A_01582 | 11.82086991 | -0.026380442 | 0.531260415 | -0.049656329 | 0.960396258 | 0.962222106 | |
| 322A_00723 | 6548.770007 | -0.007453678 | 0.166120147 | -0.044869197 | 0.964211569 | 0.965432863 | |
| 322A_01579 | 376.9686647 | 0.014831541 | 0.49618121 | 0.029891381 | 0.97615368 | 0.976771499 | |
| 322A_00291 | 856.31543 | 0.002054982 | 0.090597835 | 0.022682465 | 0.981903564 | 0.981903564 | |
| 322A_00014 | 411501.684 | -2.265558319 | 0.732009378 | -3.094985374 | NA | NA | |
| 322A_00109 | 347701.9275 | -5.217006528 | 0.840971565 | -6.203546877 | NA | NA | |
| 322A_00125 | 12581.39119 | -2.473713066 | 0.415079744 | -5.959609211 | NA | NA | |
| 322A_00182 | 10351.88878 | -3.707838041 | 0.665960492 | -5.567654662 | NA | NA | |
| 322A_00197 | 217.1178962 | -2.377528651 | 0.855912054 | -2.777772132 | NA | NA | |
| 322A_00298 | 1193.93977 | -2.27801466 | 0.536778986 | -4.243859612 | NA | NA | |
| 322A_00341 | 5326.659274 | -1.506850965 | 0.411476994 | -3.662053984 | NA | NA | |
| 322A_00432 | 2179.55067 | -1.640854034 | 0.441923223 | -3.712984402 | NA | NA | |
| 322A_00461 | 98.11498789 | -1.908922565 | 1.152345816 | -1.656553561 | NA | NA | |
| 322A_00484 | 6174.923209 | -1.699694634 | 0.651597972 | -2.608502032 | NA | NA | |
| 322A_00512 | 35772.96398 | -2.682919987 | 0.768609499 | -3.490615181 | NA | NA | |

| GeneID | Base mean | log2(FC) | StdErr | Wald-Stats | P-value | P-adj_BH | Effect of menadione on gene expression |
|---|---|---|---|---|---|---|---|
| 322A_00513 | 64030.54031 | -2.797266621 | 0.756791386 | -3.696218897 | NA | NA | |
| 322A_00625 | 4342.641505 | -1.480529413 | 0.437424779 | -3.384649162 | NA | NA | |
| 322A_00674 | 87.78648641 | -1.384733279 | 0.765028689 | -1.81004098 | NA | NA | |
| 322A_00751 | 3133.176385 | -1.535527501 | 0.487816811 | -3.147754374 | NA | NA | |
| 322A_00942 | 2360.799718 | -1.124770945 | 0.736516806 | -1.527149057 | NA | NA | |
| 322A_00955 | 165.4230762 | -2.269992135 | 0.476525149 | -4.763635535 | NA | NA | |
| 322A_00971 | 1217.82273 | -1.92047157 | 0.486896584 | -3.944311036 | NA | NA | |
| 322A_00992 | 1229.775927 | -2.829502609 | 0.506593052 | -5.585356136 | NA | NA | |
| 322A_01093 | 11713.53077 | -2.13552518 | 0.455293782 | -4.69043344 | NA | NA | |
| 322A_01143 | 0 | NA | NA | NA | NA | NA | |
| 322A_01208 | 9274.442936 | -2.687189737 | 0.543972833 | -4.939933716 | NA | NA | |
| 322A_01288 | 348.0308127 | -3.073218102 | 0.828510598 | -3.709328657 | NA | NA | |
| 322A_01330 | 17.39520005 | -1.510221562 | 0.959208792 | -1.574445078 | NA | NA | |
| 322A_01394 | 499.0638386 | -2.997147657 | 0.762190804 | -3.932280001 | NA | NA | |
| 322A_01395 | 341.2478095 | -2.615150516 | 0.476170559 | -5.492045789 | NA | NA | |
| 322A_01511 | 8065.816893 | -2.063463539 | 0.414424538 | -4.979105598 | NA | NA | |
| 322A_01520 | 3369.288341 | -3.499292367 | 0.893686381 | -3.915570877 | NA | NA | |
| 322A_01521 | 18437.85682 | -3.001740361 | 0.663668342 | -4.52295246 | NA | NA | |
| 322A_01541 | 1033.091891 | -1.11367161 | 0.513615898 | -2.168296609 | NA | NA | |
| 322A_01571 | 5554.307813 | -1.378699478 | 0.525692421 | -2.622635259 | NA | NA | |
| 322A_01581 | 256.8646115 | -1.9073005 | 0.44091267 | -4.325801067 | NA | NA | |
| 322A_01583 | 1404.653633 | -2.988892818 | 0.445558356 | -6.708196081 | NA | NA | |
| 322A_01606 | 426.8773529 | -3.36415472 | 0.641078065 | -5.247652203 | NA | NA | |

Supplementary Table 3. Results from Signalling Pathway Impact Analysis of *H. pylori* 322A's significantly differentially expressed genes mapped to the genome of *H. pylori* 26695

| **Name** | **ID** | **pSize** | **NDE** | **pNDE** | **tA** | **pPERT** | **pG** | **pGFdr** | **pGFWER** | **Status** | **KEGGLINK** |
|---|---|---|---|---|---|---|---|---|---|---|---|
| Bacterial chemotaxis | 2030 | 16 | 15 | 0.143055 | -3.91292 | 0.485 | 0.254501 | 0.391228 | 0.763503 | Inhibited | http://www.genome.jp/dbget-bin/show_pathway?hpy02030+HP_0816+HP_0815+HP_0584+HP_1030+HP_1031+HP_0352+HP_1067+HP_0392+HP_0298+HP_0019+HP_0393+HP_0616+HP_0082+HP_0099+HP_0103 |
| Two-component system | 2020 | 28 | 24 | 0.320956 | -3.33419 | 0.367 | 0.369727 | 0.391228 | 1 | Inhibited | http://www.genome.jp/dbget-bin/show_pathway?hpy02020+HP_0082+HP_0099+HP_0103+HP_0714+HP_0815+HP_1032+HP_0115+HP_0601+HP_1442+HP_0690+HP_0693+HP_0512+HP_1529+HP_1067+HP_0019+HP_0393+HP_0616+HP_0392+HP_0144+HP_0145+HP_0146+HP_0147+HP_1540+HP_1539 |
| Sulfur relay system | 4122 | 8 | 7 | 0.508121 | 1.844259 | 0.252 | 0.391228 | 0.391228 | 1 | Activated | http://www.genome.jp/dbget-bin/show_pathway?hpy04122+HP_0220+HP_0013+HP_1335+HP_0800+HP_0801+HP_0798+HP_0768 |

Supplementary Table 4. Network statistics calculated for the KEGG-based analysis of the significantly differentially expressed genes

Only genes with a degree of 1 or more are shown. Genes with degree 0 are not connected to any other genes in the network.

| GeneID | Degree | Closeness | Betweenness | KO | GenBank |
|---|---|---|---|---|---|
| 322A_00555 | 28 | NA | 0 | K15842 cytotoxicity-associated immunodominant antigen | cag pathogenicity island protein (cag26) |
| 322A_01007 | 28 | 0.001119821 | 1793.859524 | K03787 5'/3'-nucleotidase [EC:3.1.3.5 3.1.3.6] | stationary-phase survival protein (surE) |
| 322A_00914 | 20 | 0.019230769 | 291.3333333 | K00626 acetyl-CoA C-acetyltransferase [EC:2.3.1.9] | acetyl coenzyme A acetyltransferase (thiolase) (fadA) |
| 322A_00615 | 20 | 0.022222222 | 14.8 | K01895 acetyl-CoA synthetase [EC:6.2.1.1] | acetyl-CoA synthetase (acoE) |
| 322A_01089 | 18 | 9.62E-04 | 296.252381 | K00940 nucleoside-diphosphate kinase [EC:2.7.4.6] | nucleoside diphosphate kinase (ndk) |
| 322A_00514 | 16 | 0.022727273 | 289.3 | K00174 2-oxoglutarate/2-oxoacid ferredoxin oxidoreductase subunit alpha [EC:1.2.7.3 1.2.7.11] | ferredoxin oxidoreductase, alpha subunit |
| 322A_01357 | 14 | 0.020833333 | 212.9166667 | K00172 pyruvate ferredoxin oxidoreductase gamma subunit [EC:1.2.7.1] | pyruvate ferredoxin oxidoreductase, gamma subunit |
| 322A_01358 | 14 | 0.020833333 | 212.9166667 | K00171 pyruvate ferredoxin oxidoreductase delta subunit [EC:1.2.7.1] | pyruvate ferredoxin oxidoreductase, delta subunit |
| 322A_01360 | 14 | 0.020833333 | 212.9166667 | K00170 pyruvate ferredoxin oxidoreductase beta subunit [EC:1.2.7.1] | pyruvate ferredoxin oxidoreductase, beta subunit |
| 322A_00015 | 13 | 0.076923077 | 146 | K00645 [acyl-carrier-protein] S-malonyltransferase [EC:2.3.1.39] | malonyl coenzyme A-acyl carrier protein transacylase (fabD) |
| 322A_01328 | 13 | 0.01754386 | 1830.8 | K01007 pyruvate, water dikinase [EC:2.7.9.2] | phosphoenolpyruvate synthase (ppsA) |
| 322A_01085 | 12 | 0.090909091 | 299.5 | K00648 3-oxoacyl-[acyl-carrier-protein] synthase III [EC:2.3.1.180] | beta-ketoacyl-acyl carrier protein synthase III (fabH) |
| 322A_00691 | 12 | 0.001038422 | 367.0214286 | K00942 guanylate kinase [EC:2.7.4.8] | 5'-guanylate kinase (gmk) |
| 322A_01432 | 12 | 0.055555556 | 139.5 | K02160 acetyl-CoA carboxylase biotin carboxyl carrier protein | biotin carboxyl carrier protein (fabE) |
| 322A_00546 | 12 | 0.055555556 | 139.5 | K01962 acetyl-CoA carboxylase carboxyl transferase subunit alpha [EC:6.4.1.2 2.1.3.15] | acetyl-coenzyme A carboxylase (accA) |
| 322A_00984 | 12 | 0.055555556 | 139.5 | K01963 acetyl-CoA carboxylase carboxyl transferase subunit beta [EC:6.4.1.2 2.1.3.15] | acetyl-CoA carboxylase beta subunit (accD) |
| 322A_00081 | 11 | 0.014925373 | 125.7333333 | K01647 citrate synthase [EC:2.3.3.1] | citrate synthase (gltA) |
| 322A_00637 | 11 | 0.125 | 36 | K03413 two-component system, chemotaxis family, chemotaxis protein CheY | chemotaxis protein (cheY) |
| 322A_01485 | 10 | 0.004098361 | 65.5 | K01624 fructose-bisphosphate aldolase, class II [EC:4.1.2.13] | fructose-bisphosphate aldolase (tsr) |
| 322A_00932 | 10 | 0.001680672 | 2667 | K00812 aspartate aminotransferase [EC:2.6.1.1] | solute-binding signature and mitochondrial signature protein (aspB) |
| 322A_00640 | 10 | 0.027777778 | 39.66666667 | K17103 CDP-diacylglycerol---serine O-phosphatidyltransferase [EC:2.7.8.8] | phosphatidylserine synthase (pssA) |
| 322A_00819 | 10 | 0.00295858 | 452.25 | K15778 phosphomannomutase / phosphoglucomutase [EC:5.4.2.8 5.4.2.2] | phosphomannomutase (algC){Pseudomonas aeruginosa} |
| 322A_00568 | 9 | 0.002079002 | 2837 | K01915 glutamine synthetase [EC:6.3.1.2] | glutamine synthetase (glnA) |
| 322A_01197 | 9 | 0.003322259 | 2862.75 | K01810 glucose-6-phosphate isomerase [EC:5.3.1.9] | glucose-6-phosphate isomerase (pgi) |
| 322A_00080 | 8 | 0.015625 | 91.66666667 | K00031 isocitrate dehydrogenase [EC:1.1.1.42] | isocitrate dehydrogenase (icd) |
| 322A_01101 | 8 | 0.037037037 | 32.5 | K00600 glycine hydroxymethyltransferase [EC:2.1.2.1] | serine hydroxymethyltransferase (glyA) |
| 322A_01441 | 8 | 9.54E-04 | 8.035714286 | K00526 ribonucleoside-diphosphate reductase beta chain [EC:1.17.4.1] | ribonucleoside diphosphate reductase, beta subunit (nrdB) |
| 322A_00527 | 8 | 0.333333333 | 35 | K01491 methylenetetrahydrofolate dehydrogenase (NADP+) / methenyltetrahydrofolate cyclohydrolase [EC:1.5.1.5 3.5.4.9] | methylene-tetrahydrofolate dehydrogenase (folD) |
| 322A_00653 | 8 | 0.001579779 | 198 | K00609 aspartate carbamoyltransferase catalytic subunit [EC:2.1.3.2] | aspartate transcarbamoylase (pyrB) |
| 322A_01161 | 7 | 0.055555556 | 0 | K03407 two-component system, chemotaxis family, sensor kinase CheA [EC:2.7.13.3] | histidine kinase (cheA) |
| 322A_00545 | 7 | 0.066666667 | 132 | K09458 3-oxoacyl-[acyl-carrier-protein] synthase II [EC:2.3.1.179] | beta ketoacyl-acyl carrier protein synthase II (fabF) |
| 322A_00440 | 7 | 0.001012146 | 0 | K00769 xanthine phosphoribosyltransferase [EC:2.4.2.22] | xanthine guanine phosphoribosyl transferase (gpt) |
| 322A_00408 | 7 | 9.56E-04 | 118 | K01139 GTP diphosphokinase / guanosine-3',5'-bis(diphosphate) 3'-diphosphatase [EC:2.7.6.5 3.1.7.2] | penta-phosphate guanosine-3'-pyrophosphohydrolase (spoT) |
| 322A_01050 | 7 | NA | 0 | K18930 D-lactate dehydrogenase | D-lactate dehydrogenase (dld) |
| 322A_01068 | 7 | NA | 0 | K06287 nucleoside triphosphate pyrophosphatase [EC:3.6.1.-] | conserved hypothetical protein |

| GeneID | Degree | Closeness | Betweenness | KO | GenBank |
|---|---|---|---|---|---|
| 322A_00879 | 7 | 0.333333333 | 57 | K00969 nicotinate-nucleotide adenylyltransferase [EC:2.7.7.18] | conserved hypothetical protein |
| 322A_00162 | 7 | 0.003676471 | 37 | K03841 fructose-1,6-bisphosphatase I [EC:3.1.3.11] | fructose-1,6-bisphosphatase |
| 322A_00246 | 7 | 0.001029866 | 192.1380952 | K00943 dTMP kinase [EC:2.7.4.9] | thymidylate kinase (tmk) |
| 322A_00023 | 6 | 0.05 | 0 | K03406 methyl-accepting chemotaxis protein | methyl-accepting chemotaxis transducer (tlpC) |
| 322A_00005 | 6 | 0.05 | 0 | K03406 methyl-accepting chemotaxis protein | methyl-accepting chemotaxis protein (tlpA) |
| 322A_00001 | 6 | 0.05 | 0 | K03406 methyl-accepting chemotaxis protein | methyl-accepting chemotaxis protein (tlpB) |
| 322A_01144 | 6 | 0.001190476 | 1408.892857 | K01951 GMP synthase (glutamine-hydrolysing) [EC:6.3.5.2] | GMP synthase (guaA) |
| 322A_00396 | 6 | 0.012048193 | 11.4 | K01682 aconitate hydratase 2 / 2-methylisocitrate dehydratase [EC:4.2.1.3 4.2.1.99] | aconitase B (acnB) |
| 322A_00346 | 6 | 0.001013171 | 0.5 | K00088 IMP dehydrogenase [EC:1.1.1.205] | inosine-5'-monophosphate dehydrogenase (guaB) |
| 322A_00335 | 6 | 0.1 | 8 | K15894 UDP-N-acetylglucosamine 4,6-dehydratase/5-epimerase [EC:4.2.1.115 5.1.3.-] | flaA1 protein |
| 322A_00321 | 6 | 0.00128866 | 1431 | K00364 GMP reductase [EC:1.7.1.7] | GMP reductase (guaC) |
| 322A_01353 | 6 | 0.004524887 | 1010 | K00134 glyceraldehyde 3-phosphate dehydrogenase (phosphorylating) [EC:1.2.1.12] | glyceraldehyde-3-phosphate dehydrogenase (gap) |
| 322A_00834 | 6 | 0.037037037 | 11.66666667 | K01696 tryptophan synthase beta chain [EC:4.2.1.20] | tryptophan synthase, beta subunit (trpB) |
| 322A_01574 | 6 | 0.004524887 | 1010 | K00134 glyceraldehyde 3-phosphate dehydrogenase (phosphorylating) [EC:1.2.1.12] | glyceraldehyde-3-phosphate dehydrogenase (gap) |
| 322A_01576 | 6 | 0.1 | 7 | K00655 1-acyl-sn-glycerol-3-phosphate acyltransferase [EC:2.3.1.51] | 1-acyl-glycerol-3-phosphate acyltransferase (plsC) |
| 322A_00086 | 5 | NA | 0 | K03415 two-component system, chemotaxis family, chemotaxis protein CheV | chemotaxis protein (cheV) |
| 322A_01558 | 5 | 0.001014199 | 0 | K01119 2',3'-cyclic-nucleotide 2'-phosphodiesterase / 3'-nucleotidase [EC:3.1.4.16 3.1.3.6] | 2',3'-cyclic-nucleotide 2'-phosphodiesterase (cpdB) |
| 322A_01173 | 5 | 0.001845018 | 2629 | K00262 glutamate dehydrogenase (NADP+) [EC:1.4.1.4] | glutamate dehydrogenase (gdhA) |
| 322A_01160 | 5 | NA | 0 | K03415 two-component system, chemotaxis family, chemotaxis protein CheV | chemotaxis protein (cheV) |
| 322A_00532 | 5 | 0.001014199 | 3.366666667 | K00759 adenine phosphoribosyltransferase [EC:2.4.2.7] | adenine phosphoribosyltransferase (apt) |
| 322A_00530 | 5 | 0.003246753 | 3.25 | K01808 ribose 5-phosphate isomerase B [EC:5.3.1.6] | galactosidase acetyltransferase (lacA) |
| 322A_00489 | 5 | NA | 0 | K03415 two-component system, chemotaxis family, chemotaxis protein CheV | chemotaxis protein (cheV) |
| 322A_00453 | 5 | 0.001315789 | 0 | K01744 aspartate ammonia-lyase [EC:4.3.1.1] | aspartate ammonia-lyase (aspA) |
| 322A_00950 | 5 | 0.028571429 | 22 | K01079 phosphoserine phosphatase [EC:3.1.3.3] | phosphoserine phosphatase (serB) |
| 322A_00919 | 5 | 0.016666667 | 0 | K04042 bifunctional UDP-N-acetylglucosamine pyrophosphorylase / glucosamine-1-phosphate N-acetyltransferase [EC:2.7.7.23 2.3.1.157] | UDP-N-acetylglucosamine pyrophosphorylase (glmU) |
| 322A_00433 | 5 | 0.003649635 | 2.25 | K00948 ribose-phosphate pyrophosphokinase [EC:2.7.6.1] | phosphoribosylpyrophosphate synthetase (prsA) |
| 322A_00401 | 5 | 0.045454545 | 10 | K03639 GTP 3',8-cyclase [EC:4.1.99.22] | molybdenum cofactor biosynthesis protein A (moaA) |
| 322A_00371 | 5 | 0.333333333 | 8 | K14652 3,4-dihydroxy 2-butanone 4-phosphate synthase / GTP cyclohydrolase II [EC:4.1.99.12 3.5.4.25] | GTP cyclohydrolase II/3,4-dihydroxy-2-butanone 4-phosphate synthase (ribA, ribB) |
| 322A_00334 | 5 | 0.142857143 | 0 | K13038 phosphopantothenoylcysteine decarboxylase / phosphopantothenate---cysteine ligase [EC:4.1.1.36 6.3.2.5] | pantothenate metabolism flavoprotein (dfp) |
| 322A_00312 | 5 | 9.51E-04 | 94.98571429 | K01520 dUTP diphosphatase [EC:3.6.1.23] | deoxyuridine 5'-triphosphate nucleotidohydrolase (dut) |
| 322A_01008 | 5 | 1 | 6 | K00795 farnesyl diphosphate synthase [EC:2.5.1.1 2.5.1.10] | geranyltranstransferase (ispA) |
| 322A_01005 | 5 | 0.166666667 | 6 | K01737 6-pyruvoyltetrahydropterin/6-carboxytetrahydropterin synthase [EC:4.2.3.12 4.1.2.50] | predicted coding region HP0933 |
| 322A_01060 | 5 | 0.333333333 | 5 | K00796 dihydropteroate synthase [EC:2.5.1.15] | dihydropteroate synthase (folP) |
| 322A_00833 | 5 | NA | 0 | K13498 indole-3-glycerol phosphate synthase / phosphoribosylanthranilate isomerase [EC:4.1.1.48 5.3.1.24] | anthranilate isomerase (trpC) |
| 322A_01573 | 5 | 0.004739336 | 1989 | K00927 phosphoglycerate kinase [EC:2.7.2.3] | phosphoglycerate kinase |
| 322A_00175 | 5 | 0.076923077 | 17 | K01476 arginase [EC:3.5.3.1] | arginase (rocF) |
| 322A_00226 | 5 | 0.111111111 | 36 | K01928 UDP-N-acetylmuramoyl-L-alanyl-D-glutamate--2,6-diaminopimelate ligase [EC:6.3.2.13] | UDP-MurNac-tripeptide synthetase (murE) |

| GeneID | Degree | Closeness | Betweenness | KO | GenBank |
|---|---|---|---|---|---|
| 322A_00184 | 5 | 0.00257732 | 2703 | K00820 glutamine---fructose-6-phosphate transaminase (isomerizing) [EC:2.6.1.16] | glucosamine fructose-6-phosphate aminotransferase (isomerizing) (glmS) |
| 322A_00103 | 4 | 1 | 1 | K01591 orotidine-5'-phosphate decarboxylase [EC:4.1.1.23] | orotidine 5'-phosphate decarboxylase (pyrF) |
| 322A_00064 | 4 | 0.5 | 74 | K01711 GDPmannose 4,6-dehydratase [EC:4.2.1.47] | GDP-D-mannose dehydratase (rfbD) |
| 322A_00052 | 4 | 0.001862197 | 63 | K13821 RHH-type transcriptional regulator, proline utilization regulon repressor / proline dehydrogenase / delta 1-pyrroline-5-carboxylate dehydrogenase [EC:1.5.5.2 1.2.1.88] | delta-1-pyrroline-5-carboxylate dehydrogenase |
| 322A_01092 | 4 | 0.047619048 | 16 | K00208 enoyl-[acyl-carrier protein] reductase I [EC:1.3.1.9 1.3.1.10] | enoyl-(acyl-carrier-protein) reductase (NADH) (fabI) |
| 322A_01072 | 4 | 1 | 1.5 | K00981 phosphatidate cytidylyltransferase [EC:2.7.7.41] | CDP-diglyceride synthetase (cdsA) |
| 322A_00759 | 4 | 0.001385042 | 192 | K04093 chorismate mutase [EC:5.4.99.5] | predicted coding region HP0291 |
| 322A_01427 | 4 | NA | 0 | K01772 protoporphyrin/coproporphyrin ferrochelatase [EC:4.98.1.1 4.99.1.9] | ferrochelatase (hemH) |
| 322A_01252 | 4 | 0.5 | 28 | K01000 phospho-N-acetylmuramoyl-pentapeptide-transferase [EC:2.7.8.13] | phospho-N-acetylmuramoyl-pentapeptide-transferase (mraY) |
| 322A_01246 | 4 | 0.019607843 | 0 | K01058 phospholipase A1/A2 [EC:3.1.1.32 3.1.1.4] | phospholipase A1 precursor (DR-phospholipase A) |
| 322A_00543 | 4 | 0.028571429 | 101 | K00059 3-oxoacyl-[acyl-carrier protein] reductase [EC:1.1.1.100] | 3-ketoacyl-acyl carrier protein reductase (fabG) |
| 322A_00520 | 4 | 0.111111111 | 3 | K02417 flagellar motor switch protein FliN | flagellar switch protein (fliN) |
| 322A_00456 | 4 | 0.002604167 | 118 | K00963 UTP--glucose-1-phosphate uridylyltransferase [EC:2.7.7.9] | UDP-glucose pyrophosphorylase (galU) |
| 322A_00939 | 4 | 0.001234568 | 0 | K01736 chorismate synthase [EC:4.2.3.5] | chorismate synthase (aroC) |
| 322A_00903 | 4 | 0.076923077 | 0 | K00901 diacylglycerol kinase (ATP) [EC:2.7.1.107] | diacylglycerol kinase (dgkA) |
| 322A_01610 | 4 | 0.001533742 | 0 | K01424 L-asparaginase [EC:3.5.1.1] | L-asparaginase II (ansB) |
| 322A_00435 | 4 | 0.166666667 | 38 | K01929 UDP-N-acetylmuramoyl-tripeptide--D-alanyl-D-alanine ligase [EC:6.3.2.10] | UDP-MurNac-pentapeptide presynthetase (murF) |
| 322A_00373 | 4 | NA | 0 | K01497 GTP cyclohydrolase II [EC:3.5.4.25] | GTP cyclohydrolase II (ribA) |
| 322A_00360 | 4 | NA | 0 | K02556 chemotaxis protein MotA | flagellar motor rotation protein (motA) |
| 322A_00359 | 4 | NA | 0 | K02557 chemotaxis protein MotB | flagellar motor rotation protein (motB) |
| 322A_00353 | 4 | 0.142857143 | 11 | K00003 homoserine dehydrogenase [EC:1.1.1.3] | homoserine dehydrogenase (metL) |
| 322A_00331 | 4 | 1 | 1 | K00941 hydroxymethylpyrimidine/phosphomethylpyrimidine kinase [EC:2.7.1.49 2.7.4.7] | thiamine biosynthesis protein (thi) |
| 322A_00319 | 4 | 0.5 | 3 | K03272 D-beta-D-heptose 7-phosphate kinase / D-beta-D-heptose 1-phosphate adenosyltransferase [EC:2.7.1.167 2.7.7.70] | ADP-heptose synthase (rfaE) |
| 322A_00315 | 4 | 0.2 | 2 | K03525 type III pantothenate kinase [EC:2.7.1.33] | predicted coding region HP0862 |
| 322A_00306 | 4 | 1 | 1.5 | K01521 CDP-diacylglycerol pyrophosphatase [EC:3.6.1.26] | CDP-diglyceride hydrolase (cdh) |
| 322A_01351 | 4 | 0.002358491 | 1394.5 | K01955 carbamoyl-phosphate synthase large subunit [EC:6.3.5.5] | carbamoyl-phosphate synthase (glutamine-hydrolysing) (pyrAb) |
| 322A_01009 | 4 | 0.026315789 | 0 | K01495 GTP cyclohydrolase IA [EC:3.5.4.16] | GTP cyclohydrolase I (folE) |
| 322A_00982 | 4 | 0.2 | 0 | K03743 nicotinamide-nucleotide amidase [EC:3.5.1.42] | conserved hypothetical integral membrane protein |
| 322A_00975 | 4 | 0.026315789 | 0 | K22391 GTP cyclohydrolase I [EC:3.5.4.16] | conserved hypothetical protein |
| 322A_00961 | 4 | 0.009259259 | 1970 | K15633 2,3-bisphosphoglycerate-independent phosphoglycerate mutase [EC:5.4.2.12] | phosphoglycerate mutase (pgm) |
| 322A_00587 | 4 | NA | 0 | K00995 CDP-diacylglycerol---glycerol-3-phosphate 3-phosphatidyltransferase [EC:2.7.8.5] | phosphatidylglycerophosphate synthase (pgsA) |
| 322A_00590 | 4 | 0.090909091 | 14 | K12506 2-C-methyl-D-erythritol 4-phosphate cytidylyltransferase / 2-C-methyl-D-erythritol 2,4-cyclodiphosphate synthase [EC:2.7.7.60 4.6.1.12] | conserved hypothetical protein |
| 322A_00600 | 4 | 0.111111111 | 3 | K02417 flagellar motor switch protein FliN | fliY protein (fliY) |
| 322A_01455 | 4 | 0.002915452 | 0 | K00845 glucokinase [EC:2.7.1.2] | glucokinase (glk) |
| 322A_01209 | 4 | 0.003558719 | 0 | K01839 phosphopentomutase [EC:5.4.2.7] | phosphopentomutase (deoB) |
| 322A_01014 | 4 | 0.1 | 5 | K00133 aspartate-semialdehyde dehydrogenase [EC:1.2.1.11] | aspartate-semialdehyde dehydrogenase (asd) |
| 322A_00868 | 4 | 0.008130081 | 0 | K01679 fumarate hydratase, class II [EC:4.2.1.2] | fumarase (fumC) |
| 322A_01423 | 4 | 0.166666667 | 4 | K00767 nicotinate-nucleotide pyrophosphorylase (carboxylating) [EC:2.4.2.19] | nicotinate-nucleotide pyrophosphorylase (nadC) |
| 322A_01402 | 4 | 0.037037037 | 8.5 | K02372 3-hydroxyacyl-[acyl-carrier-protein] dehydratase [EC:4.2.1.59] | (3R)-hydroxymyristoyl-(acyl carrier protein) dehydratase (fabZ) |
| 322A_00209 | 4 | 0.083333333 | 4 | K08591 acyl phosphate:glycerol-3-phosphate acyltransferase [EC:2.3.1.275] | conserved hypothetical integral membrane protein |

| GeneID | Degree | Closeness | Betweenness | KO | GenBank |
| --- | --- | --- | --- | --- | --- |
| 322A_00208 | 4 | NA | 0 | K01633 7,8-dihydroneopterin aldolase/epimerase/oxygenase [EC:4.1.2.25 5.1.99.8 1.13.11.81] | conserved hypothetical protein |
| 322A_00083 | 3 | 0.25 | 3 | K03760 lipid A ethanolaminephosphotransferase [EC:2.7.8.43] | conserved hypothetical integral membrane protein |
| 322A_00060 | 3 | 1 | 6 | K10536 agmatine deiminase [EC:3.5.3.12] | predicted coding region HP0049 |
| 322A_00032 | 3 | 0.052631579 | 3 | K01428 urease subunit alpha [EC:3.5.1.5] | urease beta subunit (urea amidohydrolase) (ureB) |
| 322A_00031 | 3 | 0.052631579 | 3 | K14048 urease subunit gamma/beta [EC:3.5.1.5] | urease alpha subunit (ureA) (urea amidohydrolase) |
| 322A_01556 | 3 | 0.001519757 | 630 | K01760 cysteine-S-conjugate beta-lyase [EC:4.4.1.13] | cystathionine gamma-synthase (metB) |
| 322A_01555 | 3 | 0.001517451 | 64 | K01738 cysteine synthase [EC:2.5.1.47] | cysteine synthetase (cysK) |
| 322A_01297 | 3 | 0.012345679 | 1850 | K01689 enolase 1/2/3 [EC:4.2.1.11] | enolase (eno) |
| 322A_01090 | 3 | 0.001076426 | 0 | K00789 S-adenosylmethionine synthetase [EC:2.5.1.6] | S-adenosylmethionine synthetase 2 (metX) |
| 322A_00707 | 3 | NA | 0 | K02492 glutamyl-tRNA reductase [EC:1.2.1.70] | glutamyl-tRNA reductase (hemA) |
| 322A_00744 | 3 | 1 | 0 | K01609 indole-3-glycerol phosphate synthase [EC:4.1.1.48] | predicted coding region HP0276 |
| 322A_00761 | 3 | 0.142857143 | 0 | K03342 para-aminobenzoate synthetase / 4-amino-4-deoxychorismate lyase [EC:2.6.1.85 4.1.3.38] | para-aminobenzoate synthetase (pabB) |
| 322A_00682 | 3 | 0.25 | 0 | K01916 NAD+ synthase [EC:6.3.1.5] | NH(3)-dependentAD+ synthetase (nadE) |
| 322A_00670 | 3 | 8.82E-04 | 0 | K01937 CTP synthase [EC:6.3.4.2] | CTP synthetase (pyrG) |
| 322A_00667 | 3 | 0.5 | 3 | K02410 flagellar motor switch protein FliG | flagellar motor switch protein (fliG) |
| 322A_01439 | 3 | 0.142857143 | 9 | K15895 UDP-4-amino-4,6-dideoxy-L-N-acetyl-beta-L-altrosamine transaminase [EC:2.6.1.92] | spore coat polysaccharide biosynthesis protein C |
| 322A_01431 | 3 | 8.83E-04 | 3.666666667 | K01494 dCTP deaminase [EC:3.5.4.13] | deoxycytidine triphosphate deaminase (dcd) |
| 322A_01251 | 3 | 0.071428571 | 25 | K01925 UDP-N-acetylmuramoylalanine--D-glutamate ligase [EC:6.3.2.9] | UDP-N-acetylmuramoylalanine-D-glutamate ligase (murD) |
| 322A_00454 | 3 | 0.02 | 8 | K00790 UDP-N-acetylglucosamine 1-carboxyvinyltransferase [EC:2.5.1.7] | UDP-N-acetylglucosamine enolpyruvyl transferase (murZ) |
| 322A_00948 | 3 | 0.001164144 | 69 | K18285 aminodeoxyfutalosine synthase [EC:2.5.1.120] | conserved hypothetical protein |
| 322A_00375 | 3 | 0.166666667 | 15 | K03635 molybdopterin synthase catalytic subunit [EC:2.8.1.12] | molybdopterin converting factor, subunit 2 (moaE) |
| 322A_00330 | 3 | 1 | 3 | K00878 hydroxyethylthiazole kinase [EC:2.7.1.50] | thiamin phosphate pyrophosphorylase/hyroxyethylthiazole kinase (thiM) |
| 322A_00325 | 3 | 0.333333333 | 2 | K19302 undecaprenyl-diphosphatase [EC:3.6.1.27] | conserved hypothetical integral membrane protein |
| 322A_00310 | 3 | 0.166666667 | 12 | K00748 lipid-A-disaccharide synthase [EC:2.4.1.182] | lipid A disaccharide synthetase (lpxB) |
| 322A_00581 | 3 | NA | 0 | K00254 dihydroorotate dehydrogenase [EC:1.3.5.2] | dihydroorotate dehydrogenase (pyrD) |
| 322A_00583 | 3 | 0.333333333 | 12 | K01714 4-hydroxy-tetrahydrodipicolinate synthase [EC:4.3.3.7] | dihydrodipicolinate synthetase (dapA) |
| 322A_00601 | 3 | 0.5 | 3 | K02416 flagellar motor switch protein FliM | flagellar motor switch protein (fliM) |
| 322A_00619 | 3 | 0.1 | 8 | K00872 homoserine kinase [EC:2.7.1.39] | homoserine kinase (thrB) |
| 322A_00656 | 3 | 1 | 0 | K11753 riboflavin kinase / FMN adenylyltransferase [EC:2.7.1.26 2.7.7.2] | riboflavin biosynthesis regulatory protein (ribC) |
| 322A_01454 | 3 | 0.5 | 73 | K01057 6-phosphogluconolactonase [EC:3.1.1.31] | glucose-6-phosphate 1-dehydrogenase (devB) |
| 322A_01366 | 3 | 1 | 0 | K00681 gamma-glutamyltranspeptidase / glutathione hydrolase [EC:2.3.2.2 3.4.19.13] | gamma-glutamyltranspeptidase (ggt) |
| 322A_01049 | 3 | 0.125 | 0 | K00806 undecaprenyl diphosphate synthase [EC:2.5.1.31] | conserved hypothetical protein |
| 322A_00825 | 3 | 0.25 | 3 | K03707 thiaminase (transcriptional activator TenA) [EC:3.5.99.2] | transcriptional regulator (tenA) |
| 322A_00163 | 3 | 0.003584229 | 36.5 | K01783 ribulose-phosphate 3-epimerase [EC:5.1.3.1] | D-ribulose-5-phosphate 3 epimerase (rpe) |
| 322A_01522 | 3 | NA | 0 | K01433 formyltetrahydrofolate deformylase [EC:3.5.1.10] | formyltetrahydrofolate hydrolase (purU) |
| 322A_00245 | 3 | NA | 0 | K00954 pantetheine-phosphate adenylyltransferase [EC:2.7.7.3] | lipopolysaccharide core biosynthesis protein (kdtB) |
| 322A_00211 | 3 | 0.25 | 5 | K27502 carboxyaminopropylagmatine dehydrogenase [EC:1.5.1.55] | conserved hypothetical ATP-binding protein |
| 322A_00106 | 2 | 1 | 5 | K00794 6,7-dimethyl-8-ribityllumazine synthase [EC:2.5.1.78] | riboflavin synthase beta chain (ribE) |
| 322A_00105 | 2 | 0.1 | 4 | K01627 2-dehydro-3-deoxyphosphooctonate aldolase (KDO 8-P synthase) [EC:2.5.1.55] | 3-deoxy-d-manno-octulosonic acid 8-phosphate synthetase (kdsA) |
| 322A_00102 | 2 | 1 | 2 | K01918 pantoate--beta-alanine ligase [EC:6.3.2.1] | pantoate-beta-alanine ligase (panC) |
| 322A_00078 | 2 | 0.333333333 | 4 | K01935 dethiobiotin synthetase [EC:6.3.3.3] | dethiobiotin synthetase (bioD) |
| 322A_00063 | 2 | 0.333333333 | 0 | K02377 GDP-L-fucose synthase [EC:1.1.1.271] | nodulation protein (nolK) |
| 322A_00054 | 2 | 0.001165501 | 22.66666667 | K00558 DNA (cytosine-5)-methyltransferase 1 [EC:2.1.1.37] | adenine/cytosine DNA methyltransferase |
| 322A_00033 | 2 | 0.04 | 0 | K03191 acid-activated urea channel | urease accessory protein (ureI) |

| GeneID | Degree | Closeness | Betweenness | KO | GenBank |
|---|---|---|---|---|---|
| 322A_00028 | 2 | NA | 0 | K03431 phosphoglucosamine mutase [EC:5.4.2.10] | urease protein (ureC) |
| 322A_00006 | 2 | 0.066666667 | 5 | K01733 threonine synthase [EC:4.2.3.1] | threonine synthase (thrC) |
| 322A_01557 | 2 | 0.001385042 | 576 | K07173 S-ribosylhomocysteine lyase [EC:4.4.1.21] | conserved hypothetical protein |
| 322A_01550 | 2 | 0.003436426 | 0 | K01628 L-fuculose-phosphate aldolase [EC:4.1.2.17] | predicted coding region HP0112 |
| 322A_01294 | 2 | 0.1 | 8 | K00891 shikimate kinase [EC:2.7.1.71] | shikimic acid kinase I (aroK) |
| 322A_01289 | 2 | 0.333333333 | 8 | K01698 porphobilinogen synthase [EC:4.2.1.24] | delta-aminolevulinic acid dehydratase (hemB) |
| 322A_01489 | 2 | 1 | 7 | K03750 molybdopterin molybdotransferase [EC:2.10.1.1] | molybdopterin biosynthesis protein (moeA) |
| 322A_01106 | 2 | 1 | 0 | K15898 pseudaminic acid synthase [EC:2.5.1.97] | spore coat polysaccharide biosynthesis protein E |
| 322A_01071 | 2 | 0.058823529 | 6 | K00099 1-deoxy-D-xylulose-5-phosphate reductoisomerase [EC:1.1.1.267] | conserved hypothetical protein |
| 322A_00698 | 2 | 0.333333333 | 6 | K00979 3-deoxy-manno-octulosonate cytidylyltransferase (CMP-KDO synthetase) [EC:2.7.7.38] | CTP:CMP-3-deoxy-D-manno-octulosonate-cytidylyl-transferase (kdsB) |
| 322A_00705 | 2 | 0.166666667 | 9 | K01749 hydroxymethylbilane synthase [EC:2.5.1.61] | porphobilinogen deaminase (hemC) |
| 322A_00708 | 2 | NA | 0 | K02523 octaprenyl-diphosphate synthase [EC:2.5.1.90] | octaprenyl-diphosphate synthase (ispB) |
| 322A_00734 | 2 | 1 | 27 | K01465 dihydroorotase [EC:3.5.2.3] | dihydroorotase (pyrC) |
| 322A_00746 | 2 | 8.76E-04 | 0 | K01524 exopolyphosphatase / guanosine-5'-triphosphate,3'-diphosphate pyrophosphatase [EC:3.6.1.11 3.6.1.40] | guanosine pentaphosphate phosphohydrolase (gppA) |
| 322A_00758 | 2 | 0.071428571 | 0 | K01586 diaminopimelate decarboxylase [EC:4.1.1.20] | diaminopimelate decarboxylase (dap decarboxylase) (lysA) |
| 322A_00774 | 2 | 1 | 5 | K01845 glutamate-1-semialdehyde 2,1-aminomutase [EC:5.4.3.8] | glutamate-1-semialdehyde 2,1-aminomutase (hemL) |
| 322A_00684 | 2 | 0.333333333 | 8 | K15896 UDP-4-amino-4,6-dideoxy-N-acetyl-beta-L-altrosamine N-acetyltransferase [EC:2.3.1.202] | flagellar protein G (flaG) |
| 322A_00683 | 2 | 0.333333333 | 10 | K00912 tetraacyldisaccharide 4'-kinase [EC:2.7.1.130] | conserved hypothetical protein |
| 322A_01445 | 2 | 0.002314815 | 0 | K01784 UDP-glucose 4-epimerase [EC:5.1.3.2] | UDP-glucose 4-epimerase |
| 322A_01159 | 2 | 0.1 | 0 | K03269 UDP-2,3-diacylglucosamine hydrolase [EC:3.6.1.54] | predicted coding region HP0394 |
| 322A_01156 | 2 | 0.007462687 | 0 | K00058 D-3-phosphoglycerate dehydrogenase / 2-oxoglutarate reductase [EC:1.1.1.95 1.1.1.399] | phosphoglycerate dehydrogenase (serA) |
| 322A_01153 | 2 | 0.333333333 | 10 | K03527 4-hydroxy-3-methylbut-2-en-1-yl diphosphate reductase [EC:1.17.7.4] | penicillin tolerance protein (lytB) |
| 322A_01152 | 2 | 0.066666667 | 5 | K00800 3-phosphoshikimate 1-carboxyvinyltransferase [EC:2.5.1.19] | 3-phosphoshikimate 1-carboxyvinyltransferase (aroA) |
| 322A_01237 | 2 | 1 | 7 | K00215 4-hydroxy-tetrahydrodipicolinate reductase [EC:1.17.1.8] | dihydrodipicolinate reductase (dapB) |
| 322A_00525 | 2 | 1 | 1 | K23082 3-deoxy-D-manno-octulosonic acid-hydrolase [EC:3.2.1.144] | predicted coding region HP0579 |
| 322A_00524 | 2 | 1 | 1 | K23083 3-deoxy-D-manno-octulosonic acid-hydrolase | predicted coding region HP0580 |
| 322A_00523 | 2 | 1 | 27 | K01465 dihydroorotase [EC:3.5.2.3] | dihydroorotase (pyrC) |
| 322A_00506 | 2 | 0.1 | 0 | K00652 8-amino-7-oxononanoate synthase [EC:2.3.1.47] | 8-amino-7-oxononanoate synthase (bioF) |
| 322A_00499 | 2 | 0.066666667 | 5 | K01599 uroporphyrinogen decarboxylase [EC:4.1.1.37] | uroporphyrinogen decarboxylase (hemE) |
| 322A_00481 | 2 | 0.05 | 18 | K01924 UDP-N-acetylmuramate--alanine ligase [EC:6.3.2.8] | UDP-N-acetylmuramate-alanine ligase (murC) |
| 322A_00479 | 2 | 0.166666667 | 12 | K03526 (E)-4-hydroxy-3-methylbut-2-enyl-diphosphate synthase [EC:1.17.7.1 1.17.7.3] | protein E (gcpE) |
| 322A_00465 | 2 | 1 | 4 | K06920 7-cyano-7-deazaguanine synthase [EC:6.3.4.20] | conserved hypothetical protein |
| 322A_00937 | 2 | 0.041666667 | 0 | K02495 oxygen-independent coproporphyrinogen III oxidase [EC:1.3.98.3] | oxygen-independent coproporphyrinogen III oxidase (hemN) |
| 322A_00913 | 2 | 0.013157895 | 0 | K01028 3-oxoacid CoA-transferase subunit A [EC:2.8.3.5] | 3-oxoadipate coA-transferase subunit A (yxjD) |
| 322A_00912 | 2 | 0.013157895 | 0 | K01029 3-oxoacid CoA-transferase subunit B [EC:2.8.3.5] | 3-oxoadipate coA-transferase subunit B (yxjE) |
| 322A_00437 | 2 | 0.090909091 | 5 | K01921 D-alanine-D-alanine ligase [EC:6.3.2.4] | D-alanine:D-alanine ligase A (ddlA) |
| 322A_00434 | 2 | NA | 0 | K19710 ATP adenylyltransferase [EC:2.7.7.53] | conserved hypothetical protein |
| 322A_00416 | 2 | NA | 0 | K12251 N-carbamoylputrescine amidase [EC:3.5.1.53] | beta-alanine synthetase homolog |
| 322A_00377 | 2 | 0.1 | 12 | K03637 cyclic pyranopterin monophosphate synthase [EC:4.6.1.17] | molybdenum cofactor biosynthesis protein C (moaC) |
| 322A_00367 | 2 | 0.043478261 | 10 | K00997 holo-[acyl-carrier protein] synthase [EC:2.7.8.7] | holo-acp synthase (acpS) |
| 322A_00344 | 2 | 0.028571429 | 0 | K00859 dephospho-CoA kinase [EC:2.7.1.24] | conserved hypothetical ATP binding protein |
| 322A_00343 | 2 | 0.001164144 | 0 | K00797 spermidine synthase [EC:2.5.1.16] | spermidine synthase (speE) |
| 322A_00332 | 2 | NA | 0 | K00788 thiamine-phosphate pyrophosphorylase [EC:2.5.1.3] | thiamin phosphate pyrophosphorylase/hyroxyethylthiazole kinase (thiB) |

| GeneID | Degree | Closeness | Betweenness | KO | GenBank |
|---|---|---|---|---|---|
| 322A_00317 | 2 | 0.333333333 | 0 | K03273 D-glycero-D-manno-heptose 1,7-bisphosphate phosphatase [EC:3.1.3.82 3.1.3.83] | conserved hypothetical protein |
| 322A_01004 | 2 | 0.333333333 | 6 | K10026 7-carboxy-7-deazaguanine synthase [EC:4.3.99.3] | conserved hypothetical protein |
| 322A_00606 | 2 | 1 | 0 | K00950 2-amino-4-hydroxy-6-hydroxymethyldihydropteridine diphosphokinase [EC:2.7.6.3] | 7,8-dihydro-6-hydroxymethylpterin-pyrophosphokinase (folK) |
| 322A_00608 | 2 | 0.333333333 | 8 | K03786 3-dehydroquinate dehydratase II [EC:4.2.1.10] | 3-dehydroquinase type II (aroQ) |
| 322A_00621 | 2 | 0.058823529 | 6 | K02535 UDP-3-O-[3-hydroxymyristoyl] N-acetylglucosamine deacetylase [EC:3.5.1.108] | UDP-3-0-acyl-acetylglcosamine deacetylase (envA) |
| 322A_01451 | 2 | 0.5 | 0 | K01625 2-dehydro-3-deoxyphosphogluconate aldolase / (4S)-4-hydroxy-2-oxoglutarate aldolase [EC:4.1.2.14 4.1.3.42] | 2-keto-3-deoxy-6-phosphogluconate aldolase (eda) |
| 322A_01452 | 2 | NA | 0 | K01690 phosphogluconate dehydratase [EC:4.2.1.12] | 6-phosphogluconate dehydratase |
| 322A_01369 | 2 | 0.001165501 | 22.66666667 | K00558 DNA (cytosine-5)-methyltransferase 1 [EC:2.1.1.37] | cytosine specific DNA methyltransferase (BSP6IM) |
| 322A_01039 | 2 | 0.001295337 | 0 | K00640 serine O-acetyltransferase [EC:2.3.1.30] | serine acetyltransferase (cysE) |
| 322A_01052 | 2 | 0.1 | 8 | K01719 uroporphyrinogen-III synthase [EC:4.2.1.75] | uroporphyrinogen III cosynthase (hemD) |
| 322A_01066 | 2 | 0.001841621 | 0 | K01455 formamidase [EC:3.5.1.49] | aliphatic amidase (aimE) |
| 322A_00793 | 2 | 0.166666667 | 9 | K00014 shikimate dehydrogenase [EC:1.1.1.25] | shikimate 5-dehydrogenase (aroE) |
| 322A_00831 | 2 | 0.001270648 | 0 | K01658 anthranilate synthase component II [EC:4.1.3.27] | anthranilate synthase component II (trpD) |
| 322A_00830 | 2 | 0.001270648 | 0 | K01657 anthranilate synthase component I [EC:4.1.3.27] | anthranilate synthase component I (trpE) |
| 322A_01421 | 2 | NA | 0 | K01613 phosphatidylserine decarboxylase [EC:4.1.1.65] | phosphatidylserine decarboxylase proenzyme (psd) |
| 322A_01397 | 2 | 0.001519757 | 252 | K04517 prephenate dehydrogenase [EC:1.3.1.12] | prephenate dehydrogenase (tyrA) |
| 322A_00169 | 2 | NA | 0 | K00858 NAD+ kinase [EC:2.7.1.23] | conserved hypothetical protein |
| 322A_01256 | 2 | 1 | 3 | K01012 biotin synthase [EC:2.8.1.6] | biotin synthetase (bioB) |
| 322A_01505 | 2 | 0.037037037 | 14 | K00075 UDP-N-acetylmuramate dehydrogenase [EC:1.3.1.98] | UDP-N-acetylenolpyruvoylglucosamine reductase (murB) |
| 322A_00278 | 2 | 0.066666667 | 0 | K00919 4-diphosphocytidyl-2-C-methyl-D-erythritol kinase [EC:2.7.1.148] | conserved hypothetical protein |
| 322A_00244 | 2 | 0.333333333 | 2 | K03186 flavin prenyltransferase [EC:2.5.1.129] | phenylacrylic acid decarboxylase |
| 322A_00236 | 2 | 1 | 1 | K08973 protoporphyrinogen IX oxidase [EC:1.3.99.-] | conserved hypothetical integral membrane protein |
| 322A_00151 | 2 | 0.2 | 0 | K11754 dihydrofolate synthase / folylpolyglutamate synthase [EC:6.3.2.12 6.3.2.17] | folylpolyglutamate synthase (folC) |
| 322A_00126 | 2 | 0.166666667 | 6 | K03270 3-deoxy-D-manno-octulosonate 8-phosphate phosphatase (KDO 8-P phosphatase) [EC:3.1.3.45] | conserved hypothetical protein |
| 322A_00122 | 2 | NA | 0 | K00793 riboflavin synthase [EC:2.5.1.9] | riboflavin synthase alpha subunit (ribC) |
| 322A_00117 | 2 | NA | 0 | K23159 lipid A 4'-phosphatase [EC:3.1.3.-] | predicted coding region HP1580 |
| 322A_00085 | 1 | NA | 0 | K27684 carboxyaminopropylagmatine decarboxylase [EC:4.1.1.127] | carboxynorspermidine decarboxylase (nspC) |
| 322A_00084 | 1 | 0.125 | 0 | K12977 lipid A 1-phosphatase [EC:3.1.3.-] | predicted coding region HP0021 |
| 322A_00073 | 1 | NA | 0 | K01579 aspartate 1-decarboxylase [EC:4.1.1.11] | aspartate 1-decarboxylase (panD) |
| 322A_01318 | 1 | NA | 0 | K01626 3-deoxy-7-phosphoheptulonate synthase [EC:2.5.1.54] | 3-deoxy-D-arabino-heptulosonate 7-phosphate synthase (dhs1) |
| 322A_01299 | 1 | NA | 0 | K11785 5,8-dihydroxy-2-naphthoate synthase [EC:4.1.99.29] | predicted coding region HP0152 |
| 322A_01096 | 1 | NA | 0 | K25995 succinate dehydrogenase iron-sulfur subunit [EC:1.3.5.1 7.1.1.12] | fumarate reductase, iron-sulfur subunit (frdB) |
| 322A_01095 | 1 | NA | 0 | K00233 succinate dehydrogenase flavoprotein subunit [EC:1.3.5.1 7.1.1.12] | fumarate reductase, flavoprotein subunit (frdA) |
| 322A_01094 | 1 | NA | 0 | K25996 succinate dehydrogenase subunit C | fumarate reductase, cytochrome b subunit (frdC) |
| 322A_01086 | 1 | NA | 0 | K03621 phosphate acyltransferase [EC:2.3.1.274] | fatty acid/phospholipid synthesis protein (plsX) |
| 322A_01075 | 1 | 0.045454545 | 0 | K01439 succinyl-diaminopimelate desuccinylase [EC:3.5.1.18] | succinyl-diaminopimelate desuccinylase (dapE) |
| 322A_00748 | 1 | NA | 0 | K02517 Kdo2-lipid IVA lauroyltransferase/acyltransferase [EC:2.3.1.241 2.3.1.-] | heat shock protein B (ibpB) |
| 322A_00694 | 1 | 1 | 0 | K07226 heme oxygenase (biliverdin-IX-beta and delta-forming) [EC:1.14.99.58] | conserved hypothetical protein |
| 322A_00665 | 1 | 0.041666667 | 0 | K01662 1-deoxy-D-xylulose-5-phosphate synthase [EC:2.2.1.7] | deoxyxylulose-5-phosphate synthase, putative (dxs) |
| 322A_01157 | 1 | NA | 0 | K03182 4-hydroxy-3-polyprenylbenzoate decarboxylase [EC:4.1.1.98] | conserved hypothetical protein |
| 322A_01135 | 1 | 1 | 0 | K01874 methionyl-tRNA synthetase [EC:6.1.1.10] | methionyl-tRNA synthetase (metS) |

| GeneID | Degree | Closeness | Betweenness | KO | GenBank |
|---|---|---|---|---|---|
| 322A_01544 | 1 | 1 | 0 | K01885 glutamyl-tRNA synthetase [EC:6.1.1.17] | glutamyl-tRNA synthetase (gltX) |
| 322A_01238 | 1 | NA | 0 | K00104 glycolate dehydrogenase FAD-linked subunit [EC:1.1.99.14] | glycolate oxidase subunit (glcD) |
| 322A_00570 | 1 | 1 | 0 | K12086 cag pathogenicity island protein 1 | cag pathogenicity island protein (cag1) |
| 322A_00572 | 1 | 1 | 0 | K12088 cag pathogenicity island protein 3 | cag pathogenicity island protein (cag3) |
| 322A_00573 | 1 | 1 | 0 | K12089 cag pathogenicity island protein 4 | cag pathogenicity island protein (cag4) |
| 322A_00575 | 1 | 1 | 0 | K12090 cag pathogenicity island protein 5 | cag pathogenicity island protein (cag5) |
| 322A_00576 | 1 | 1 | 0 | K03196 type IV secretion system protein VirB11 [EC:7.4.2.8] | virB11 homolog |
| 322A_00577 | 1 | 1 | 0 | K12091 cag pathogenicity island protein 6 | cag pathogenicity island protein (cag6) |
| 322A_00578 | 1 | 1 | 0 | K12092 cag pathogenicity island protein 7 | cag pathogenicity island protein (cag7) |
| 322A_01219 | 1 | 1 | 0 | K12093 cag pathogenicity island protein 8 | cag pathogenicity island protein (cag8) |
| 322A_01220 | 1 | 1 | 0 | K12094 cag pathogenicity island protein 9 | cag pathogenicity island protein (cag9) |
| 322A_01221 | 1 | 1 | 0 | K12095 cag pathogenicity island protein 10 | cag pathogenicity island protein (cag10) |
| 322A_01222 | 1 | 1 | 0 | K12096 cag pathogenicity island protein 11 | cag pathogenicity island protein (cag11) |
| 322A_01223 | 1 | 1 | 0 | K12097 cag pathogenicity island protein 12 | cag pathogenicity island protein (cag12) |
| 322A_01224 | 1 | 1 | 0 | K12098 cag pathogenicity island protein 13 | cag pathogenicity island protein (cag13) |
| 322A_01225 | 1 | 1 | 0 | K12099 cag pathogenicity island protein 14 | cag pathogenicity island protein (cag14) |
| 322A_01227 | 1 | 1 | 0 | K12101 cag pathogenicity island protein 16 | cag pathogenicity island protein (cag16) |
| 322A_01228 | 1 | 1 | 0 | K12102 cag pathogenicity island protein 17 | cag pathogenicity island protein (cag17) |
| 322A_01229 | 1 | 1 | 0 | K12103 cag pathogenicity island protein 18 | cag pathogenicity island protein (cag18) |
| 322A_01230 | 1 | 1 | 0 | K12104 cag pathogenicity island protein 19 | cag pathogenicity island protein (cag19) |
| 322A_01231 | 1 | 1 | 0 | K12105 cag pathogenicity island protein 20 | cag pathogenicity island protein (cag20) |
| 322A_01232 | 1 | 1 | 0 | K12106 cag pathogenicity island protein 21 | cag pathogenicity island protein (cag21) |
| 322A_01233 | 1 | 1 | 0 | K12107 cag pathogenicity island protein 22 | cag pathogenicity island protein (cag22) |
| 322A_01234 | 1 | 1 | 0 | K12108 cag pathogenicity island protein 23 [EC:7.4.2.8] | cag pathogenicity island protein (cag23) |
| 322A_01235 | 1 | 1 | 0 | K12109 cag pathogenicity island protein 24 | cag pathogenicity island protein (cag24) |
| 322A_01236 | 1 | 1 | 0 | K12110 cag pathogenicity island protein 25 | cag pathogenicity island protein (cag25) |
| 322A_00554 | 1 | 0.05 | 0 | K01776 glutamate racemase [EC:5.1.1.3] | glutamate racemase (glr) |
| 322A_00534 | 1 | NA | 0 | K01255 leucyl aminopeptidase [EC:3.4.11.1] | aminopeptidase a/i (pepA) |
| 322A_00478 | 1 | NA | 0 | K00674 2,3,4,5-tetrahydropyridine-2,6-dicarboxylate N-succinyltransferase [EC:2.3.1.117] | tetrahydrodipicolinate-succinyltransferase (dapD) |
| 322A_00459 | 1 | 1 | 0 | K01885 glutamyl-tRNA synthetase [EC:6.1.1.17] | glutamyl-tRNA synthetase (gltX) |
| 322A_00438 | 1 | 1 | 0 | K01095 phosphatidylglycerophosphatase A [EC:3.1.3.27] | conserved hypothetical integral membrane protein |
| 322A_00374 | 1 | 0.1 | 0 | K03636 sulfur-carrier protein | molybdopterin converting factor, subunit 1 (moaD) |
| 322A_00320 | 1 | 0.2 | 0 | K03271 D-sedoheptulose 7-phosphate isomerase [EC:5.3.1.28] | phosphoheptose isomerase (gmhA) |
| 322A_00318 | 1 | NA | 0 | K03274 ADP-L-glycero-D-manno-heptose 6-epimerase [EC:5.1.3.20] | ADP-L-glycero-D-mannoheptose-6-epimerase (rfaD) |
| 322A_00996 | 1 | 0.058823529 | 0 | K01775 alanine racemase [EC:5.1.1.1] | alanine racemase, biosynthetic (alr) |
| 322A_00994 | 1 | NA | 0 | K00285 D-amino-acid dehydrogenase [EC:1.4.5.1] | D-amino acid dehydrogenase (dadA) |
| 322A_00973 | 1 | NA | 0 | K00057 glycerol-3-phosphate dehydrogenase (NAD(P)+) [EC:1.1.1.94] | glycerol-3-phosphate dehydrogenase,AD(P)+ dependent |
| 322A_00580 | 1 | NA | 0 | K00937 polyphosphate kinase [EC:2.7.4.1] | polyphosphate kinase (ppk) |
| 322A_00627 | 1 | 1 | 0 | K00606 3-methyl-2-oxobutanoate hydroxymethyltransferase [EC:2.1.2.11] | 3-methyl-2-oxobutanoate hydroxymethyltransferase (panB) |
| 322A_01379 | 1 | 0.333333333 | 0 | K02114 F-type H+-transporting ATPase subunit epsilon | ATP synthase F1, subunit epsilon (atpC) |
| 322A_01380 | 1 | 0.333333333 | 0 | K02112 F-type H+/Na+-transporting ATPase subunit beta [EC:7.1.2.2 7.2.2.1] | ATP synthase F1, subunit beta (atpD) |
| 322A_01381 | 1 | 0.333333333 | 0 | K02115 F-type H+-transporting ATPase subunit gamma | ATP synthase F1, subunit gamma (atpG) |
| 322A_01382 | 1 | 0.333333333 | 0 | K02111 F-type H+/Na+-transporting ATPase subunit alpha [EC:7.1.2.2 7.2.2.1] | ATP synthase F1, subunit alpha (atpA) |
| 322A_01384 | 1 | 0.333333333 | 0 | K02109 F-type H+-transporting ATPase subunit b | ATP synthase F0, subunit b (atpF) |
| 322A_01385 | 1 | 0.333333333 | 0 | K02109 F-type H+-transporting ATPase subunit b | ATP synthase F0, subunit b' (atpF') |
| 322A_01184 | 1 | NA | 0 | K02563 UDP-N-acetylglucosamine--N-acetylmuramyl-(pentapeptide) pyrophosphoryl-undecaprenol N-acetylglucosamine transferase [EC:2.4.1.227] | transferase, peptidoglycan synthesis (murG) |

| GeneID | Degree | Closeness | Betweenness | KO | GenBank |
|---|---|---|---|---|---|
| 322A_01187 | 1 | 0.001663894 | 0 | K00286 pyrroline-5-carboxylate reductase [EC:1.5.1.2] | pyrroline-5-carboxylate reductase (proC) |
| 322A_01042 | 1 | 0.333333333 | 0 | K02110 F-type H+-transporting ATPase subunit c | ATP synthase F0, subunit c (atpE) |
| 322A_00798 | 1 | 0.04 | 0 | K02169 malonyl-CoA O-methyltransferase [EC:2.1.1.197] | biotin synthesis protein (bioC) |
| 322A_01422 | 1 | 0.090909091 | 0 | K03517 quinolinate synthase [EC:2.5.1.72] | quinolinate synthetase A (nadA) |
| 322A_01403 | 1 | 0.041666667 | 0 | K00677 UDP-N-acetylglucosamine acyltransferase [EC:2.3.1.129] | UDP-N-acetylglucosamine acyltransferase (lpxA) |
| 322A_01499 | 1 | NA | 0 | K09457 7-cyano-7-deazaguanine reductase [EC:1.7.1.13] | conserved hypothetical protein |
| 322A_01508 | 1 | 1 | 0 | K03196 type IV secretion system protein VirB11 [EC:7.4.2.8] | conjugative transfer regulon protein (trbB) |
| 322A_01516 | 1 | 0.066666667 | 0 | K06041 arabinose-5-phosphate isomerase [EC:5.3.1.13] | polysialic acid capsule expression protein (kpsF) |
| 322A_00253 | 1 | NA | 0 | K00826 branched-chain amino acid aminotransferase [EC:2.6.1.42] | branched-chain-amino-acid aminotransferase (ilvE) |
| 322A_00240 | 1 | 1 | 0 | K01875 seryl-tRNA synthetase [EC:6.1.1.11] | seryl-tRNA synthetase (serS) |
| 322A_00205 | 1 | NA | 0 | K01042 L-seryl-tRNA(Ser) seleniumtransferase [EC:2.9.1.1] | selenocysteine synthase SelA, putative |
| 322A_00114 | 1 | 0.047619048 | 0 | K00097 4-hydroxythreonine-4-phosphate dehydrogenase [EC:1.1.1.262] | pyridoxal phosphate biosynthetic protein A (pdxA) |

Supplementary Table 5. Over-representation analysis of significantly differentially expressed genes associated with KEGG metabolic pathways

| hpy KEGG pathway | No. of genes in pathway | P value | Adjusted P value | 322A genes in pathway |
|---|---|---|---|---|
| 01240 Biosynthesis of cofactors | 73/88 | 6.828E-27 | 6.691E-25 | 322A_00106 322A_00103 322A_00102 322A_00078 322A_00073 322A_00016 322A_01299 322A_01289 322A_01489 322A_01101 322A_01092 322A_01090 322A_01089 322A_01595 322A_00705 322A_00707 322A_00723 322A_00734 322A_00735 322A_00761 322A_00774 322A_00682 322A_00670 322A_01427 322A_01157 322A_01544 322A_00545 322A_00543 322A_00527 322A_00523 322A_00506 322A_00499 322A_00487 322A_00459 322A_00456 322A_00948 322A_00946 322A_00937 322A_00401 322A_00410 322A_00397 322A_00377 322A_00376 322A_00375 322A_00373 322A_00371 322A_00344 322A_00334 322A_00332 322A_00331 322A_00330 322A_00315 322A_01351 322A_01009 322A_01005 322A_00975 322A_00959 322A_00581 322A_00606 322A_00627 322A_00653 322A_00656 322A_01361 322A_01052 322A_01060 322A_01065 322A_00798 322A_00801 322A_00825 322A_00821 322A_00879 322A_01423 322A_01422 322A_01418 322A_01402 322A_00169 322A_01256 322A_00253 322A_00245 322A_00244 322A_00237 322A_00236 322A_00213 322A_00208 322A_00151 322A_00122 322A_00115 322A_00114 |
| 01230 Biosynthesis of amino acids | 49/57 | 8.218E-20 | 4.027E-18 | 322A_00081 322A_00080 322A_00006 322A_01557 322A_01556 322A_01555 322A_01320 322A_01318 322A_01297 322A_01294 322A_01485 322A_01101 322A_01093 322A_01090 322A_01075 322A_00744 322A_00751 322A_00758 322A_00759 322A_00680 322A_01156 322A_01152 322A_01237 322A_00568 322A_00538 322A_00530 322A_00478 322A_00950 322A_00939 322A_00932 322A_00433 322A_00396 322A_00353 322A_01353 322A_00961 322A_00584 322A_00583 322A_00608 322A_00619 322A_00657 322A_01187 322A_01014 322A_01039 322A_01057 322A_00793 322A_00835 322A_00834 322A_00833 322A_00831 322A_00830 322A_01573 322A_01574 322A_01397 322A_00163 322A_00175 322A_00253 322A_00225 |
| 05120 Epithelial cell signaling in Helicobacter pylori infection | 35/39 | 9.048E-16 | 2.956E-14 | 322A_00033 322A_00032 322A_00031 322A_01492 322A_01142 322A_01253 322A_00570 322A_00572 322A_00573 322A_00575 322A_00576 322A_00577 322A_00578 322A_01219 322A_01220 322A_01221 322A_01222 322A_01223 322A_01224 322A_01225 322A_01226 322A_01227 322A_01228 322A_01229 322A_01230 322A_01231 322A_01232 322A_01233 322A_01234 322A_01235 322A_01236 322A_00555 322A_00378 322A_00292 322A_01346 322A_01347 322A_01615 322A_00282 322A_01508 |
| 01200 Carbon metabolism | 38/49 | 7.869E-13 | 1.928E-11 | 322A_00081 322A_00080 322A_00019 322A_01555 322A_01328 322A_01320 322A_01297 322A_01485 322A_01101 322A_01093 322A_01433 322A_01432 322A_01156 322A_01563 322A_00546 322A_00530 322A_00527 322A_00515 322A_00514 322A_00513 322A_00512 322A_00950 322A_00914 322A_00433 322A_00396 322A_00302 322A_01353 322A_00984 322A_00961 322A_00615 322A_00657 322A_01451 322A_01452 322A_01453 322A_01454 322A_01455 322A_01357 322A_01358 322A_01359 322A_01360 322A_01197 322A_01039 322A_01066 322A_00868 322A_01573 322A_01574 322A_00162 322A_00163 322A_00225 |
| 02040 Flagellar assembly | 31/37 | 2.333E-12 | 4.573E-11 | 322A_00017 322A_01547 322A_01488 322A_00713 322A_00714 322A_00763 322A_00687 322A_00668 322A_00667 322A_00666 322A_00520 322A_00502 322A_00890 322A_00421 322A_00420 322A_00403 322A_00366 322A_00360 322A_00359 322A_00307 322A_01340 322A_01341 322A_01342 322A_00997 322A_00600 322A_00601 322A_00602 322A_00611 322A_00661 322A_01367 322A_01506 322A_01507 322A_00243 322A_00139 322A_00138 322A_00137 322A_00112 |
| 01250 Biosynthesis of nucleotide sugars | 25/28 | 1.807E-11 | 2.951E-10 | 322A_00105 322A_00065 322A_00064 322A_00063 322A_00028 322A_01106 322A_00698 322A_00686 322A_00685 322A_00684 322A_01445 322A_01439 322A_00456 322A_00454 322A_00923 322A_00919 322A_00335 322A_00320 322A_00319 322A_00318 322A_00317 322A_01455 322A_01197 322A_00819 322A_01505 322A_01516 322A_00184 322A_00126 |
| 02020 Two-component system | 26/31 | 1.409E-10 | 1.972E-09 | 322A_00086 322A_00023 322A_00005 322A_00001 322A_01547 322A_01308 322A_01307 322A_01306 322A_01305 322A_00712 322A_01162 322A_01161 322A_01160 322A_00568 322A_00502 322A_00489 322A_00471 322A_00914 322A_00911 322A_00910 322A_00890 322A_00360 322A_00589 322A_00602 322A_00637 322A_01055 322A_00872 322A_00279 322A_00187 322A_00157 322A_00156 |
| 00540 Lipopolysaccharide biosynthesis | 23/26 | 1.857E-10 | 2.275E-09 | 322A_00105 322A_00084 322A_00083 322A_01292 322A_01091 322A_00698 322A_00747 322A_00748 322A_00683 322A_01159 322A_00525 322A_00524 322A_00320 322A_00319 322A_00318 322A_00317 322A_00310 322A_00977 322A_00609 322A_00621 322A_01016 322A_01403 322A_01502 322A_01516 322A_00126 322A_00117 |
| 00190 Oxidative phosphorylation | 28/35 | 2.323E-10 | 2.530E-09 | 322A_01308 322A_01307 322A_01306 322A_01305 322A_01096 322A_01095 322A_01094 322A_00484 322A_00347 322A_00580 322A_01379 322A_01380 322A_01381 322A_01382 322A_01383 322A_01384 322A_01385 322A_01042 322A_01055 322A_00804 322A_00805 322A_00806 322A_00807 322A_00808 322A_00809 322A_00810 322A_00811 322A_00812 322A_00813 322A_00814 322A_00815 322A_00816 322A_00817 322A_00157 322A_00156 |
| 03010 Ribosome | 36/54 | 3.925E-09 | 3.846E-08 | 322A_00027 322A_00022 322A_00021 322A_01325 322A_01324 322A_01087 322A_00764 322A_00765 322A_01154 322A_01254 322A_00566 322A_00552 322A_00542 322A_00610 322A_01176 322A_01180 322A_01019 322A_01020 322A_01022 322A_01023 322A_01024 322A_01025 322A_01029 322A_00788 322A_00790 322A_00837 322A_00839 322A_00840 322A_00841 322A_00845 322A_00846 322A_00847 322A_00848 322A_00849 NA 322A_00850 322A_00851 322A_00852 322A_00853 322A_00854 322A_00855 322A_00856 322A_00857 322A_00858 322A_00859 322A_00860 322A_00861 322A_00862 322A_00863 NA 322A_00224 322A_00142 NA |
| 00970 Aminoacyl-tRNA biosynthesis | 32/46 | 5.671E-09 | 5.052E-08 | 322A_01327 322A_01102 322A_00706 322A_00693 322A_01151 322A_01150 322A_01135 322A_01544 322A_00488 322A_00459 322A_00944 322A_00407 322A_00345 322A_00293 322A_00974 322A_00963 322A_00960 322A_01389 322A_01182 322A_01015 322A_01069 322A_00797 322A_01509 322A_00240 322A_00205 322A_00149 322A_00100 322A_00099 322A_00696 322A_00720 |

| hpy KEGG pathway | No. of genes in pathway | P value | Adjusted P value | 322A genes in pathway |
|---|---|---|---|---|
| | | | | 322A_01447 322A_01446 322A_01440 NA 322A_01138 322A_01330 322A_00992 322A_00990 322A_00989 322A_01211 322A_01028 322A_01032 322A_01033 322A_01034 322A_00189 |
| 02010 ABC transporters | 23/29 | 1.355E-08 | 1.107E-07 | 322A_00718 322A_00719 322A_00766 322A_00767 322A_00768 322A_00769 322A_00770 322A_01443 322A_01540 322A_01541 322A_01542 322A_01543 322A_00889 322A_00425 322A_00424 322A_00357 322A_00356 322A_00998 322A_00997 322A_00651 322A_00795 322A_00796 322A_00257 322A_00256 322A_00255 322A_00222 322A_00132 322A_00120 322A_00119 |
| 00550 Peptidoglycan biosynthesis | 14/14 | 1.770E-08 | 1.157E-07 | 322A_01252 322A_01251 322A_00507 322A_00481 322A_00454 322A_00437 322A_00435 322A_00325 322A_01184 322A_01049 322A_01505 322A_00226 322A_00140 322A_00131 |
| 03060 Protein export | 14/14 | 1.770E-08 | 1.157E-07 | 322A_00029 322A_00692 322A_00528 322A_00411 322A_00388 322A_00629 322A_00630 322A_01181 322A_00799 322A_00844 322A_00272 322A_00147 322A_00146 322A_00145 |
| 03430 Mismatch repair | 14/14 | 1.770E-08 | 1.157E-07 | 322A_00727 322A_00671 322A_01245 322A_00490 322A_00483 322A_00887 322A_01345 322A_01059 322A_00789 322A_00791 322A_00164 322A_00261 322A_00242 322A_00238 |
| 00790 Folate biosynthesis | 17/19 | 3.584E-08 | 2.195E-07 | 322A_01489 322A_00761 322A_00465 322A_00401 322A_00402 322A_00377 322A_00376 322A_00375 322A_00373 322A_00371 322A_01009 322A_01005 322A_01004 322A_00975 322A_00606 322A_01060 322A_01499 322A_00208 322A_00151 |
| 00010 Glycolysis / Gluconeogenesis | 18/21 | 5.727E-08 | 3.185E-07 | 322A_01328 322A_01297 322A_01485 322A_01093 322A_00514 322A_00513 322A_01353 322A_00961 322A_00615 322A_01455 322A_01456 322A_00173 322A_01357 322A_01358 322A_01359 322A_01360 322A_01197 322A_00819 322A_01573 322A_01574 322A_00162 |
| 02030 Bacterial chemotaxis | 15/16 | 5.850E-08 | 3.185E-07 | 322A_00086 322A_00023 322A_00005 322A_00001 322A_00766 322A_00667 322A_01162 322A_01161 322A_01160 322A_00520 322A_00489 322A_00360 322A_00359 322A_00600 322A_00601 322A_00637 |
| 00520 Amino sugar and nucleotide sugar metabolism | 17/20 | 1.766E-07 | 9.110E-07 | 322A_00065 322A_00064 322A_00063 322A_00028 322A_01106 322A_00686 322A_00685 322A_00684 322A_01445 322A_01439 322A_00456 322A_00454 322A_00923 322A_00919 322A_00335 322A_01455 322A_01197 322A_00819 322A_01505 322A_00184 |
| 03440 Homologous recombination | 14/15 | 1.969E-07 | 9.648E-07 | 322A_01298 322A_00671 322A_01166 322A_01245 322A_00887 322A_00300 322A_00296 322A_01012 322A_00628 322A_01059 322A_00789 322A_00791 322A_00164 322A_00261 322A_00193 |
| 00620 Pyruvate metabolism | 18/22 | 2.327E-07 | 9.916E-07 | 322A_00019 322A_01328 322A_01096 322A_01095 322A_01094 322A_01433 322A_01432 322A_00546 322A_00514 322A_00513 322A_00914 322A_00362 322A_00984 322A_00615 322A_01456 322A_00173 322A_01357 322A_01358 322A_01359 322A_01360 322A_01050 322A_00868 |
| 00630 Glyoxylate and dicarboxylate metabolism | 12/12 | 2.274E-07 | 9.916E-07 | 322A_00081 322A_01101 322A_01563 322A_01238 322A_00568 322A_00914 322A_00396 322A_00302 322A_00615 322A_01451 322A_01066 322A_01522 |
| 00860 Porphyrin metabolism | 12/12 | 2.274E-07 | 9.916E-07 | 322A_01289 322A_00705 322A_00707 322A_00774 322A_00694 322A_01427 322A_01544 322A_00499 322A_00459 322A_00937 322A_01052 322A_00236 |
| 03070 Bacterial secretion system | 15/17 | 3.683E-07 | 1.504E-06 | 322A_00692 322A_01265 322A_00576 322A_00411 322A_00388 322A_00292 322A_01274 322A_00629 322A_00630 322A_01181 322A_00799 322A_00844 322A_01508 322A_00272 322A_00147 322A_00146 322A_00145 |
| 00680 Methane metabolism | 13/14 | 6.598E-07 | 2.586E-06 | 322A_01328 322A_01297 322A_01485 322A_01101 322A_01156 322A_01146 322A_00950 322A_00961 322A_00615 322A_01357 322A_01358 322A_01359 322A_01360 322A_00162 |
| 00564 Glycerophospholipid metabolism | 11/11 | 8.149E-07 | 2.958E-06 | 322A_01072 322A_01246 322A_00903 322A_00438 322A_00306 322A_00973 322A_00587 322A_00640 322A_01576 322A_01421 322A_00209 |
| 00900 Terpenoid backbone biosynthesis | 11/11 | 8.149E-07 | 2.958E-06 | 322A_01071 322A_00708 322A_00665 322A_01153 322A_00479 322A_00914 322A_01008 322A_00590 322A_01049 322A_00278 322A_00244 |
| 00400 Phenylalanine, tyrosine and tryptophan biosynthesis | 14/16 | 1.168E-06 | 4.089E-06 | 322A_01318 322A_01294 322A_00744 322A_00751 322A_00759 322A_01152 322A_00939 322A_00932 322A_00608 322A_00793 322A_00835 322A_00834 322A_00833 322A_00831 322A_00830 322A_01397 |
| 00720 Other carbon fixation pathways | 17/22 | 2.232E-06 | 7.291E-06 | 322A_00080 322A_01328 322A_01096 322A_01095 322A_01094 322A_01433 322A_01432 322A_00546 322A_00527 322A_00515 322A_00514 322A_00513 322A_00512 322A_00914 322A_00396 322A_00984 322A_00615 322A_01357 322A_01358 322A_01359 322A_01360 322A_00868 |
| 02024 Quorum sensing | 12/13 | 2.200E-06 | 7.291E-06 | 322A_01557 322A_01318 322A_00411 322A_00388 322A_00373 322A_01181 322A_00799 322A_00831 322A_00830 322A_00844 322A_00272 322A_00213 322A_00145 |
| 00230 Purine metabolism | 18/24 | 2.339E-06 | 7.394E-06 | 322A_00032 322A_00031 322A_01558 322A_01089 322A_00723 322A_00746 322A_00691 322A_01441 322A_01144 322A_00532 322A_00487 322A_00922 322A_00440 322A_00434 322A_00433 322A_00408 322A_00346 322A_00321 322A_01007 322A_01361 322A_01208 322A_01209 322A_01047 322A_00819 |
| 00770 Pantothenate and CoA biosynthesis | 10/10 | 2.919E-06 | 8.939E-06 | 322A_00102 322A_00073 322A_00680 322A_00367 322A_00344 322A_00334 322A_00315 322A_00627 322A_00253 322A_00245 |
| 00270 Cysteine and methionine metabolism | 15/19 | 5.766E-06 | 1.712E-05 | 322A_00058 322A_00054 322A_00055 322A_00016 322A_01557 322A_01556 322A_01555 322A_01320 322A_01090 322A_01156 322A_00932 322A_00353 322A_00343 322A_00619 322A_01369 322A_01014 322A_01039 322A_01057 322A_00253 |

| hpy KEGG pathway | No. of genes in pathway | P value | Adjusted P value | 322A genes in pathway |
|---|---|---|---|---|
| 03030 DNA replication | 11/12 | 7.290E-06 | 2.101E-05 | 322A_00093 322A_01245 322A_00490 322A_00941 322A_00887 322A_01059 322A_00789 322A_00791 322A_00866 322A_01416 322A_00164 322A_00261 |
| 00330 Arginine and proline metabolism | 10/11 | 2.400E-05 | 6.533E-05 | 322A_00085 322A_00060 322A_00052 322A_00762 322A_01130 322A_00932 322A_00416 322A_00343 322A_01187 322A_00175 322A_00211 |
| 01212 Fatty acid metabolism | 10/11 | 2.400E-05 | 6.533E-05 | 322A_00015 322A_01092 322A_01085 322A_01433 322A_01432 322A_00546 322A_00545 322A_00543 322A_00914 322A_00984 322A_01402 |
| 00240 Pyrimidine metabolism | 15/21 | 4.441E-05 | 1.176E-04 | 322A_00103 322A_01558 322A_01089 322A_00734 322A_00670 322A_01449 322A_01441 322A_01431 322A_00523 322A_00922 322A_00410 322A_00312 322A_01351 322A_01007 322A_00581 322A_00653 322A_01065 322A_01068 322A_00801 322A_00246 322A_00183 |
| 00061 Fatty acid biosynthesis | 9/10 | 7.836E-05 | 2.021E-04 | 322A_00015 322A_01092 322A_01085 322A_01433 322A_01432 322A_00546 322A_00545 322A_00543 322A_00984 322A_01402 |
| 00300 Lysine biosynthesis | 10/12 | 1.077E-04 | 2.639E-04 | 322A_01075 322A_00758 322A_01237 322A_00538 322A_00478 322A_00435 322A_00353 322A_00584 322A_00583 322A_01014 322A_01057 322A_00226 |
| 00650 Butanoate metabolism | 10/12 | 1.077E-04 | 2.639E-04 | 322A_01096 322A_01095 322A_01094 322A_00514 322A_00513 322A_00914 322A_00913 322A_00912 322A_01357 322A_01358 322A_01359 322A_01360 |
| 00030 Pentose phosphate pathway | 11/14 | 1.232E-04 | 2.944E-04 | 322A_01485 322A_00530 322A_00433 322A_00657 322A_01451 322A_01452 322A_01453 322A_01454 322A_01197 322A_01209 322A_00819 322A_00162 322A_00163 322A_00225 |
| 03410 Base excision repair | 7/7 | 1.339E-04 | 3.052E-04 | 322A_01310 322A_00671 322A_00519 322A_00490 322A_00452 322A_01575 322A_00190 |
| 03420 Nucleotide excision repair | 7/7 | 1.339E-04 | 3.052E-04 | 322A_00490 322A_00898 322A_00354 322A_01345 322A_01363 322A_00242 322A_00155 |
| 00760 Nicotinate and nicotinamide metabolism | 8/9 | 2.535E-04 | 5.573E-04 | 322A_00682 322A_01007 322A_00982 322A_01208 322A_00803 322A_00879 322A_01423 322A_01422 322A_00169 |
| 01232 Nucleotide metabolism | 14/21 | 2.559E-04 | 5.573E-04 | 322A_01089 322A_00723 322A_00691 322A_00670 322A_01441 322A_01431 322A_01144 322A_00532 322A_00487 322A_00922 322A_00440 322A_00410 322A_00346 322A_00321 322A_00312 322A_01007 322A_01361 322A_01208 322A_01068 322A_00246 322A_00183 |
| 00250 Alanine, aspartate and glutamate metabolism | 10/13 | 3.494E-04 | 7.286E-04 | 322A_00052 322A_00723 322A_01173 322A_00568 322A_00453 322A_00932 322A_01610 322A_01351 322A_00653 322A_01361 322A_01065 322A_00174 322A_00184 |
| 00541 O-Antigen nucleotide sugar biosynthesis | 10/13 | 3.494E-04 | 7.286E-04 | 322A_00065 322A_00064 322A_00063 322A_01106 322A_00686 322A_00685 322A_00684 322A_01445 322A_01439 322A_00456 322A_00923 322A_00919 322A_00335 |
| 00561 Glycerolipid metabolism | 6/6 | 4.791E-04 | 9.583E-04 | 322A_01086 322A_00903 322A_01456 322A_00173 322A_01576 322A_00209 |
| 00910 Nitrogen metabolism | 6/6 | 4.791E-04 | 9.583E-04 | 322A_00104 322A_01173 322A_00568 322A_00406 322A_01217 322A_01066 |
| 00020 Citrate cycle (TCA cycle) | 11/16 | 8.255E-04 | 1.498E-03 | 322A_00081 322A_00080 322A_00019 322A_01096 322A_01095 322A_01094 322A_00515 322A_00514 322A_00513 322A_00512 322A_00396 322A_01357 322A_01358 322A_01359 322A_01360 322A_00868 |
| 00220 Arginine biosynthesis | 7/8 | 8.103E-04 | 1.498E-03 | 322A_00032 322A_00031 322A_01173 322A_00568 322A_00932 322A_01351 322A_01065 322A_00175 |
| 00740 Riboflavin metabolism | 7/8 | 8.103E-04 | 1.498E-03 | 322A_00106 322A_00373 322A_00371 322A_00330 322A_00656 322A_00244 322A_00213 322A_00122 |
| 01210 2-Oxocarboxylic acid metabolism | 11/16 | 8.255E-04 | 1.498E-03 | 322A_00081 322A_00080 322A_00680 322A_00515 322A_00514 322A_00513 322A_00512 322A_00932 322A_00396 322A_01357 322A_01358 322A_01359 322A_01360 322A_01014 322A_01057 322A_00253 |
| 04122 Sulfur relay system | 7/8 | 8.103E-04 | 1.498E-03 | 322A_00092 322A_01595 322A_00401 322A_00377 322A_00376 322A_00375 322A_00374 322A_00877 |
| 00260 Glycine, serine and threonine metabolism | 10/14 | 9.165E-04 | 1.633E-03 | 322A_00006 322A_01320 322A_01101 322A_01449 322A_01156 322A_00950 322A_00353 322A_00961 322A_00619 322A_00640 322A_01014 322A_01057 322A_00835 322A_00834 |
| 00640 Propanoate metabolism | 8/10 | 9.551E-04 | 1.642E-03 | 322A_01433 322A_01432 322A_00546 322A_00396 322A_00984 322A_00615 322A_01357 322A_01358 322A_01359 322A_01360 |
| 00780 Biotin metabolism | 8/10 | 9.551E-04 | 1.642E-03 | 322A_00078 322A_01092 322A_00545 322A_00543 322A_00506 322A_00959 322A_01388 322A_00798 322A_01402 322A_01256 |
| 00450 Selenocompound metabolism | 5/5 | 1.714E-03 | 2.847E-03 | 322A_01556 322A_01135 322A_00350 322A_01195 322A_00205 |
| 01502 Vancomycin resistance | 5/5 | 1.714E-03 | 2.847E-03 | 322A_01252 322A_00437 322A_00435 322A_00996 322A_01184 |
| 00470 D-Amino acid metabolism | 6/7 | 2.553E-03 | 4.170E-03 | 322A_00758 322A_01251 322A_00554 322A_00538 322A_00437 322A_00996 322A_00994 |
| 00051 Fructose and mannose metabolism | 7/9 | 2.762E-03 | 4.438E-03 | 322A_00065 322A_00064 322A_00063 322A_01550 322A_01485 322A_01093 322A_00530 322A_00819 322A_00162 |

| hpy KEGG pathway | No. of genes in pathway | P value | Adjusted P value | 322A genes in pathway |
|---|---|---|---|---|
| 00052 Galactose metabolism | 4/4 | 6.129E-03 | 8.834E-03 | 322A_01445 322A_00456 322A_01455 322A_00819 |
| 00280 Valine, leucine and isoleucine degradation | 4/4 | 6.129E-03 | 8.834E-03 | 322A_00914 322A_00913 322A_00912 322A_00253 |
| 00380 Tryptophan metabolism | 4/4 | 6.129E-03 | 8.834E-03 | 322A_00762 322A_01563 322A_00914 322A_00302 |
| 00460 Cyanoamino acid metabolism | 4/4 | 6.129E-03 | 8.834E-03 | 322A_01101 322A_01610 322A_01366 322A_01066 |
| 00500 Starch and sucrose metabolism | 4/4 | 6.129E-03 | 8.834E-03 | 322A_00456 322A_01455 322A_01197 322A_00819 |
| 01503 Cationic antimicrobial peptide (CAMP) resistance | 4/4 | 6.129E-03 | 8.834E-03 | 322A_00083 322A_00405 322A_00589 322A_01403 |
| 03018 RNA degradation | 8/12 | 5.990E-03 | 8.834E-03 | 322A_00095 322A_01553 322A_01297 322A_00715 322A_00553 322A_00464 322A_00413 322A_00580 322A_01043 322A_01056 322A_00792 322A_01517 |
| 00710 Carbon fixation by Calvin cycle | 7/10 | 6.986E-03 | 9.923E-03 | 322A_01485 322A_01093 322A_00530 322A_01353 322A_00657 322A_01573 322A_01574 322A_00162 322A_00163 322A_00225 |
| 00730 Thiamine metabolism | 6/8 | 7.788E-03 | 1.090E-02 | 322A_01595 322A_00665 322A_00487 322A_00332 322A_00331 322A_00330 322A_00825 322A_00821 |
| 00040 Pentose and glucuronate interconversions | 3/3 | 2.191E-02 | 2.863E-02 | 322A_01550 322A_00456 322A_00163 |
| 00360 Phenylalanine metabolism | 3/3 | 2.191E-02 | 2.863E-02 | 322A_00762 322A_00932 322A_00994 |
| 00750 Vitamin B6 metabolism | 3/3 | 2.191E-02 | 2.863E-02 | 322A_00006 322A_00115 322A_00114 |
| 01501 beta-Lactam resistance | 3/3 | 2.191E-02 | 2.863E-02 | 322A_00507 322A_00140 322A_00131 |
| 03020 RNA polymerase | 3/3 | 2.191E-02 | 2.863E-02 | 322A_00409 322A_01021 322A_00838 |
| 00261 Monobactam biosynthesis | 4/5 | 2.381E-02 | 3.030E-02 | 322A_01237 322A_00584 322A_00583 322A_01014 322A_01057 |
| 00480 Glutathione metabolism | 4/5 | 2.381E-02 | 3.030E-02 | 322A_00080 322A_00534 322A_00343 322A_01453 322A_01366 |
| 00074 Mycolic acid biosynthesis | 2/2 | 7.833E-02 | 8.625E-02 | 322A_00015 322A_00367 |
| 00290 Valine, leucine and isoleucine biosynthesis | 2/2 | 7.833E-02 | 8.625E-02 | 322A_00680 322A_00253 |
| 00362 Benzoate degradation | 2/2 | 7.833E-02 | 8.625E-02 | 322A_00914 322A_01013 |
| 00401 Novobiocin biosynthesis | 2/2 | 7.833E-02 | 8.625E-02 | 322A_00932 322A_01397 |
| 00410 beta-Alanine metabolism | 2/2 | 7.833E-02 | 8.625E-02 | 322A_00102 322A_00073 |
| 00430 Taurine and hypotaurine metabolism | 2/2 | 7.833E-02 | 8.625E-02 | 322A_01366 322A_00174 |
| 00521 Streptomycin biosynthesis | 2/2 | 7.833E-02 | 8.625E-02 | 322A_01455 322A_00819 |
| 00627 Aminobenzoate degradation | 2/2 | 7.833E-02 | 8.625E-02 | 322A_00762 322A_00244 |
| 00920 Sulfur metabolism | 2/2 | 7.833E-02 | 8.625E-02 | 322A_01555 322A_01039 |
| 00999 Biosynthesis of various plant secondary metabolites | 2/2 | 7.833E-02 | 8.625E-02 | 322A_01090 322A_00793 |
| 01220 Degradation of aromatic compounds | 2/2 | 7.833E-02 | 8.625E-02 | 322A_01013 322A_00244 |
| 04148 Efferocytosis | 2/2 | 7.833E-02 | 8.625E-02 | 322A_00175 322A_00156 |
| 00130 Ubiquinone and other terpenoid-quinone biosynthesis | 5/10 | 1.182E-01 | 1.287E-01 | 322A_00016 322A_01299 322A_00735 322A_01157 322A_00948 322A_00946 322A_00397 322A_01418 322A_00244 322A_00237 |
| 00670 One carbon pool by folate | 3/6 | 2.196E-01 | 2.365E-01 | 322A_01101 322A_00527 322A_01389 322A_01522 322A_00183 |

| hpy KEGG pathway | No. of genes in pathway | P value | Adjusted P value | 322A genes in pathway |
|---|---|---|---|---|
| 00071 Fatty acid degradation | 1/1 | 2.799E-01 | 2.828E-01 | 322A_00914 |
| 00121 Secondary bile acid biosynthesis | 1/1 | 2.799E-01 | 2.828E-01 | 322A_00585 |
| 00552 Teichoic acid biosynthesis | 1/1 | 2.799E-01 | 2.828E-01 | 322A_00325 |
| 00565 Ether lipid metabolism | 1/1 | 2.799E-01 | 2.828E-01 | 322A_01246 |
| 00592 alpha-Linolenic acid metabolism | 1/1 | 2.799E-01 | 2.828E-01 | 322A_01246 |
| 00643 Styrene degradation | 1/1 | 2.799E-01 | 2.828E-01 | 322A_00762 |
| 00543 Exopolysaccharide biosynthesis | 1/2 | 4.816E-01 | 4.816E-01 | 322A_01039 |
